# Outcomes of ICU patients with and without perceptions of excessive care: a comparison between cancer and non-cancer patients

**DOI:** 10.1186/s13613-021-00895-5

**Published:** 2021-07-31

**Authors:** Dominique D. Benoit, Esther N. van der Zee, Michael Darmon, An K. L. Reyners, Victoria Metaxa, Djamel Mokart, Alexander Wilmer, Pieter Depuydt, Andreas Hvarfner, Katerina Rusinova, Jan G. Zijlstra, François Vincent, Dimitrios Lathyris, Anne-Pascale Meert, Jacques Devriendt, Emma Uyttersprot, Erwin J. O. Kompanje, Ruth Piers, Elie Azoulay

**Affiliations:** 1 Department of Intensive Care Medicine, Ghent University Hospital, C. Heymanslaan 10, 9000 Ghent, Belgium; 2 Department of Intensive Care Medicine, Erasmus MC University Medical Center Rotterdam, Rotterdam, The Netherlands; 3 Hôpital Saint-Louis and University, Paris, France; 4 Department of Medical Oncology, University Medical Center Groningen, Groningen, The Netherlands; 5 Department of Critical Care, King’s College Hospital, London, UK; 6 Critical Care and Anesthesiology, Institut Paoli Calmettes, Marseille, France; 7 Department of General Internal Medicine, University Hospital Louvain, Louvain, Belgium; 8 Department of Perioperative Medicine and Intensive Care, Karolinska University Hospital, Stockholm, Sweden; 9 Department of Palliative Care, 1St Faculty of Medicine, Charles University of Medicine, Prague, Czech Republic; 10 Department of Critical Care, University Medical Center Groningen, University of Groningen, Groningen, Netherlands; 11 Medical Surgical ICU, GHIC Le Raincy-Montfermeil, Montfermeil, France; 12 Department of Intensive Care Medicine, General Hospital G. Gennimatas, Thessaloniki, Greece; 13 Service de Médecine Interne, Université Libre de Bruxelles (ULB), soins intensifs et urgences oncologiques, Institut Jules Bordet, Brussels, Belgium; 14 Intensive Care Unit, Brugmann University Hospital, Brussels, Belgium; 15 Department of Applied Mathematics and Computer Sciences, Ghent University, Ghent, Belgium; 16 Department of Geriatric Medicine, Ghent University Hospital, Ghent, Belgium

**Keywords:** Cancer, Critical care, ICU, Bias, Perception of care, Prognostication, Treatment limitation

## Abstract

**Background:**

Whether Intensive Care Unit (ICU) clinicians display unconscious bias towards cancer patients is unknown. The aim of this study was to compare the outcomes of critically ill patients with and without perceptions of excessive care (PECs) by ICU clinicians in patients with and without cancer.

**Methods:**

This study is a sub-analysis of the large multicentre DISPROPRICUS study. Clinicians of 56 ICUs in Europe and the United States completed a daily questionnaire about the appropriateness of care during a 28-day period. We compared the cumulative incidence of patients with concordant PECs, treatment limitation decisions (TLDs) and death between patients with uncontrolled and controlled cancer, and patients without cancer.

**Results:**

Of the 1641 patients, 117 (7.1%) had uncontrolled cancer and 270 (16.4%) had controlled cancer. The cumulative incidence of concordant PECs in patients with uncontrolled and controlled cancer versus patients without cancer was 20.5%, 8.1%, and 9.1% (*p* < 0.001 and *p* = 0.62, respectively). In patients with concordant PECs, we found no evidence for a difference in time from admission until death (HR 1.02, 95% CI 0.60–1.72 and HR 0.87, 95% CI 0.49–1.54) and TLDs (HR 0.81, 95% CI 0.33–1.99 and HR 0.70, 95% CI 0.27–1.81) across subgroups. In patients without concordant PECs, we found differences between the time from admission until death (HR 2.23, 95% CI 1.58–3.15 and 1.66, 95% CI 1.28–2.15), without a corresponding increase in time until TLDs (NA, *p* = 0.3 and 0.7) across subgroups.

**Conclusions:**

The absence of a difference in time from admission until TLDs and death in patients with concordant PECs makes bias by ICU clinicians towards cancer patients unlikely. However, the differences between the time from admission until death, without a corresponding increase in time until TLDs, suggest prognostic unawareness, uncertainty or optimism in ICU clinicians who did not provide PECs, more specifically in patients with uncontrolled cancer. This study highlights the need to improve intra- and interdisciplinary ethical reflection and subsequent decision-making at the ICU.

**Supplementary Information:**

The online version contains supplementary material available at 10.1186/s13613-021-00895-5.

## Background

Over the last four decades, long-term survival in cancer patients has increased considerably thanks to improvement in diagnostics and new therapeutic advances [1–3]. The incidence of cancer patients requiring intensive care unit (ICU) treatment, due to serious infectious or chemotherapy-related events has been increasing as well [1]. Recent data show that 5–6.5% of patients with solid cancer and up to 10% of patients with a hematological malignancy are admitted to ICU during the course of their disease [1–4].

This results in an ICU bed occupancy by cancer patients of 15–20% [5–7]. Although these numbers suggest that the initial reluctance to admit cancer patients to the ICU has probably been decreasing over the past decades, unconscious bias towards this patient population during ICU stay remains a matter of concern for hematologists and oncologists in daily practice [1, 8]. According to hematologists and oncologists, ICU clinicians are often too pessimistic regarding short-term and long-term prognosis of cancer patients, while hematologists and oncologists are often too optimistic according to the ICU clinicians [8]. This may result in overt conflicts or in more subtle chronic conflicts such as animosity, distrust or communication gaps, neither of which benefit the patients and their families.

The large multicentre DISPROPRICUS study provides a unique opportunity to explore the potential issue of unconscious bias towards critically ill patient subgroups [9]. In this 28-day study, clinicians were asked to provide daily perceptions of disproportionate care (either “excessive care” or “not enough care”) in patients for whom they were directly in charge. Subsequently, these patients were followed until one year after ICU admission. The probability of being alive at home with a good quality of life one year after ICU admission in patients who were perceived as receiving excessive care by at least 2 different ICU clinicians was 7.3%. This probability was significantly higher (45.4%) in patients who were not perceived as receiving excessive care [9]. A different relationship between written treatment-limitation decisions (TLDs) and outcome across different subgroups of patients, such as cancer patients, may be indicative of unconscious bias by ICU clinicians towards these subgroups.

The aim of this sub-analysis was to compare the following three objectives: (1) time until written TLDs, (2) time until death and (3) the combined endpoint (death, poor quality of life or not being at home) between patients with and without concordant perceptions of excessive care (PECs), across the subgroups of patients with uncontrolled cancer, controlled cancer and no cancer. We hypothesized that, relative to patients without cancer, ICU clinicians do not discriminate against patients with uncontrolled or controlled cancer.

## Methods

### Study design and data collection

This study is part of the large multicentre DISPROPRICUS study [9]. The aim of the DISPROPRICUS study was to assess whether the quality of the ethical climate in an ICU is associated with the predictive value of PECs, regarding patients’ one-year outcomes, as well as the time from PECs until TLDs and time until death. We refer to previous publications for the detailed protocol and study design [9, 10]. In brief, doctors and nurses working in 68 ICUs in 12 European countries and the United States were invited to complete the ethical decision-making climate questionnaire (EDMCQ), in order to assess the ethical climate prevailing in their ICU. The EDMCQ has been subjectively validated in 2992 ICU clinicians in 68 ICUs in Europe and the US by Van den buckle et al. [10] and objectively validated at the patients’ level by Benoit et al. [9]. During the 28-day study period (between May 4 and July 4, 2014), the clinicians anonymously completed a questionnaire about their perceptions of disproportionate care for each of their patients every day.

Disproportionate care was defined as care that is no longer consistent with the expected survival or quality of life (either “too much” or “not enough” care), or that is provided against the patient’s or family’s wishes. Patient characteristics and outcomes were prospectively collected in patients admitted for reasons other than monitoring only. This included demographic data (age, gender), substance abuse (alcohol, active smoking), the Eastern Cooperative Oncology Group (ECOG) performance status 14 days prior to the ICU admission, underlying comorbidities, main admission reasons, vital organ support and written TLDs on ICU admission and during ICU stay. Patients with uncontrolled cancer were defined as ‘patients with disease progression under therapy or relapse’ and patients with controlled cancer as ‘patients with complete remission or stable partial remission’. We refer to Additional file 1: Table S1 for the definition of all other collected data. Patients who were discharged alive from the ICU or hospital were contacted one year after ICU admission. The interviewer collected vital status, place of residence and health-related quality of life, using the EuroQol-5D questionnaire. Similar to the DISPROPRICUS study, the combined patient outcome at one year was defined as dead, not at home or a utility score < 0.5 [9].

Using factor [10] and cluster analysis [9], four different mutually exclusive ethical climates were identified in the DISPROPRICUS study: good, average with (+) and without (−) nurses’ involvement at end-of-life, and poor. A significant difference in patient population between the ICUs in the average (-) ethical climate and the other climates was seen in the DISPROPRICUS study, based on ICU mortality and length of stay [9]. In the main DISPROPRICUS study, this was not an issue, because of an a priori decision that only the ‘good’ and the ‘poor’ climate were compared. However, including the units of the average (−) ethical climate in the current analysis would have potentially biased our results. Therefore, data from 12 of the 68 participating ICUs were excluded from this sub-analysis.

Congruent with the main analysis, only perceptions of excessive (“too much”) care were included in this sub- analysis because (1) “not enough care” represented only 8% of the perceptions and (2) including “too much “ and “not enough” perceptions by different clinicians for a same patient would have considerably increased the complexity of the analysis. For practical reasons, “PECs by at least two different clinicians” is referred to as “concordant PECs” throughout the entire manuscript.

The study was approved by the ethics committees of all participating centers and the Danish National Health Authority. Informed consent was required in all countries to collect the one-year outcomes.

### Data analysis

We compared outcomes between cancer subgroups in patients with PECs and without PECs by at least two different clinicians, since previous publications have highlighted the importance of concordance between two clinicians [9, 11–14].

Pearson’s Chi-square tests were used for comparing categorical variables between subgroups and Kruskal–Wallis tests (or Anova tests) for comparing continued variables. Fisher’s exact tests were used to compare differences between two groups. Results were expressed as number and percentage or median and 25–75th percentiles.

Time from admission until concordant PECs, and from concordant PECs until written TLD or death were compared using (cause-specific) hazard ratios, obtained via Cox regression (accounting for competing risk) [9]. The cause-specific hazard of an event expresses the instantaneous risk of that event at a given time for patients who are still alive at the ICU at that time and have not previously experienced that event [9].

To adjust for differential case-mix, ICU ethical decision-making climate, hospital and country characteristics between the subgroup of patients with uncontrolled cancer, controlled cancer and no cancer, we used inverse probability weighting based on propensity scores [15]. Here, the propensity score is the probability of being categorized according to one of the subgroups, as obtained using a multinomial model based on patient, ICU ethical decision-making climate, hospital and country characteristics. Included patient characteristics were age, admission reason, surgery before admission, comorbidities, alcohol problems, the patient competence, TLD before admission, cancer status and ECOG performance status. ICU and hospital characteristics were number of ICU beds, patient–nurse ratio, patient–junior physician ratio and number of hospital beds. Adjustment based on propensity scores has the advantage, relative to other adjustment methods, of preventing model extrapolation, when subgroups are very different in terms of these characteristics [9]. These are expressed as proportions and (cause-specific) hazard ratios (HR) along with 95% confidence intervals (95% CI). The reference value in all analyses was the subgroup ‘patients without cancer’. Two-sided *p* values were considered significant at the 0.05 level. We refer to Additional file 2: the master dissertation of Uyttersprot, for a more detailed methodology.

We may assume that the relationship between time until death and time until written TLD reflects prognostic estimation, and thus treatment decisions of ICU clinicians. Relative increases of hazard of death versus TLD across subgroups may thus reflect prognostic (over-)optimism across these subgroups, relative decreases may reflect (over-)pessimism. Similarly, a divergence between PECs and the risk of reaching the combined endpoint of death, poor quality-of-life or not being at home at one year may reflect over-optimistic or over-pessimistic prognostication by ICU clinicians.

Such divergences could be considered as a surrogate marker of unconscious bias, provided that important confounders (like disease severity) are adjusted for. Therefore, we considered the weighted analyses as our principal results. Unweighted results are provided in the Additional file 3: Fig. S1, Additional file 4: Fig. S2, Additional file 5: Table S3.

## Results

**Fig. 1:**
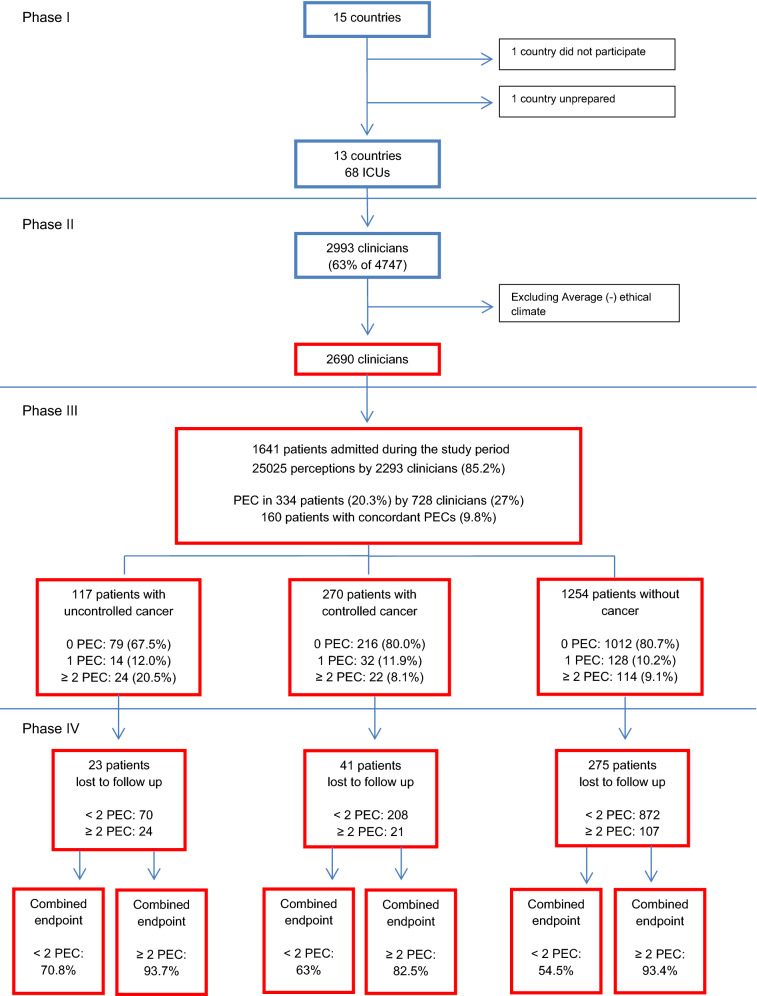
Flowchart study: number of ICU’s, clinicians, perceptions and patients. *PEC* perceptions of excessive care. Combined endpoint: death, poor quality of life or not being at home

During the study period, 1641 patients were admitted for more than monitoring to the 56 ICUs included in this sub-analysis, of which 117 patients (7.1%) had uncontrolled and 270 (16.4%) controlled cancer (Table 1). The different types of cancer are reported in Table 2. Of the 2690 participating clinicians, 2293 (85.2%) completed the questionnaire about perception of disproportionate care. In total, 25,025 perceptions were collected, of which 2279 (9.1%) PECs. PECs were given by 728 clinicians (27%) in 334 patients (20.3%). Of these 334 patients, 160 (9.8%) had concordant PECs (Fig. 1); 53 (33.1%) concordant PECs by at least two doctors, 52 (32.5%) by at least one nurse and one doctor, and 55 (34.3%) by at least two nurses. We found no evidence for a difference in the combination of clinicians who provided concordant PECs across subgroups (*p* = 0.51).

**Table 1:** Country, hospital, ICU and patient characteristics across subgroups

	Uncontrolled cancer (*n* = 117)	Controlled cancer (*n* = 270)	Without cancer (*n* = 1254)	*P*-value
Country characteristics				
Number of ICU beds/100.000 inhabitants	13.8 (11.6–15.9)	11.6 (6.4–15.9)	11.6 (6.4–15.9)	0.001
Geographical region				< 0.001
Central Europe	15 (12.8%)	40 (14.8%)	147 (11.7%)	
Northern Europe	7 (6.0%)	27 (10.0%)	257 (20.5%)	
Southern Europe	7 (6.0%)	32 (11.9%)	98 (7.8%)	
Western Europe/USA	88 (75.2%)	171 (63.3%)	752 (60.0%)	
Hospital characteristics				
Hospital type				0.002
Public	20 (17.1%)	47 (17.4%)	318 (25.4%)	
Private	10 (8.5%)	7 (2.6%)	77 (6.1%)	
University-affiliated	28 (23.9%)	43 (15.9%)	178 (14.2%)	
University	59 (50.4%)	173 (64.1%)	681 (54.3%)	
Total beds in hospital				< 0.001
< 250	20 (17.1%)	17 (6.3%)	85 (6.8%)	
250–499	33 (28.2%)	58 (21.5%)	271 (21.6%)	
500–749	15 (12.8%)	70 (25.9%)	336 (26.8%)	
> 750	49 (41.9%)	125 (46.3%)	562 (44.8%)	
ICU characteristics				
Ethical climate				0.13
Good	26 (22.2%)	54 (20.0%)	241 (19.2%)	
Average +	48 (41.0%)	97 (35.9%)	552 (44.0%)	
Poor	43 (36.8%)	119 (44.1%)	461 (36.8%)	
Number of beds per ICU	12 (8–22)	12.5 (10–25)	14 (10–24)	0.02
Patient-to-nurse ratio	2 (2–2)	2 (1.4–3)	2 (1.3–3)	0.04
Patient-to-junior physician ratio	5 (3–7)	4 (3–6)	4 (3–6)	0.09
Patient-to-senior physician ratio	7 (6–8)	7 (5–8)	7 (4–8)	0.08
Percentage of population > 65 year in ICU^a^	18 (18–18)	18 (18–18)	18 (18–18)	< 0.001
Patient characteristics				
Age	66 (58–73)	67 (57–75)	63 (50–74)	0.002
Gender	63 (53.8)%	171 (63.3)%	736 (58.7)%	0.18
ECOG performance status				< 0.001
Grade 0	20 (17.1%)	78 (28.9%)	475 (37.9%)	
Grade 1	30 (25.6%)	89 (33.0%)	285 (22.7%)	
Grade 2	26 (22.2%)	45 (16.7%)	169 (13.5%)	
Grade 3	21 (17.9%)	27 (10.0%)	156 (12.4%)	
Grade 4	9 (7.7%)	16 (5.9%)	57 (4.5%)	
Unknown	11 (9.4%)	15 (5.6%)	112 (8.9%)	
Nursing home resident	3 (2.6%)	6 (2.2%)	69 (5.5%)	0.03
Moderate-to-severe comorbidities				< 0.001
0	0 (0.0%)	0 (0.0%)	790 (63.0%)	
1	90 (76.9%)	197 (73.0%)	379 (30.2%)	
≥ 2	27 (23.1%)	73 (23.0%)	85 (6.8%)	
Type comorbidity				
Heart failure (NYHA III or IV)	5 (4.3%)	21 (7.8%)	166 (13.2%)	< 0.001
COPD (Gold III or IV or equivalent)	12 (10.3%)	35 (13.0%)	143 (11.4%)	0.69
Neurological (excluding dementia)	7 (6.0%)	7 (2.6%)	89 (7.1%)	0.02
Liver cirrhosis (Child–Pugh B or C)	0 (0.0%)	10 (3.7%)	72 (5.7%)	< 0.001
Chronic renal failure requiring dialysis	4 (3.4%)	4 (1.5%)	46 (3.7%)	0.16
Dementia (moderate or severe)	1 (0.9%)	6 (2.2%)	32 (2.6%)	0.67
AIDS	0 (0.0%)	1 (0.4%)	13 (1.0%)	< 0.001
Abuse				
Alcohol	1 (0.9%)	14 (5.2%)	166 (13.2%)	< 0.001
Smoking	10 (8.5%)	39 (14.4%)	241 (19.2%)	0.005
Main admission reason				
Respiratory failure	36 (30.8%)	65 (24.1%)	289 (23.0%)	0.17
Sepsis/severe sepsis/septic shock	34 (29.1%)	67 (24.8%)	222 (17.7%)	< 0.001
Heart failure/cardiogenic shock	11 (9.4%)	24 (8.9%)	244 (19.5%)	< 0.001
Neurologic pathology/stroke/ICB	8 (6.8%)	17 (6.3%)	157 (12.5%)	0.002
Gastro-intestinal pathology/liver failure	10 (8.5%)	26 (9.6%)	133 (10.6%)	0.72
Metabolic/renal	14 (12.0%)	22 (8.1%)	108 (8.6%)	0.44
Multiple trauma	0 (0.0%)	4 (1.5%)	92 (7.3%)	< 0.001
Head trauma	1 (0.9%)	2 (0.7%)	55 (4.4%)	0.003
Surgery within 48 h	38 (32.5%)	122 (45.2%)	100 (31.9%)	< 0.001
Surgery				< 0.001
No surgery	79 (67.5%)	141 (52.2%)	850 (67.8%)	
Scheduled surgery	19 (16.2%)	77 (28.5%)	111 (8.9%)	
Unscheduled surgery	19 (16.2%)	52 (19.3%)	293 (23.4%)	
Do-not-resuscitate order before ICU admission				< 0.001
Full code	87 (74.4%)	224 (90.4%)	1132 (90.3%)	
Unknown	8 (6.8%)	10 (3.7%)	57 (4.5%)	
No CPR	14 (12.0%)	13 (4.8%)	34 (2.7%)	
Withholding therapy	8 (6.8%)	3 (1.1%)	31 (2.5%)	
Severity of illness < 24 h after admission				
Invasive mechanical ventilation	42 (35.9%)	126 (46.7%)	616 (49.1%)	0.02
Vasopressor need	34 (29.1%)	106 (39.3%)	443 (35.3%)	0.15
Dialysis	3 (2.6%)	5 (1.9%)	47 (3.7%)	0.31
Written withholding/withdrawing order < 24 h	11 (9.4%)	6 (2.2%)	50 (4.0%)	0.008
Characteristics during ICU stay				
Non-missing patients	n = 93	n = 253	n = 1165	0.20
Invasive mechanical ventilation	46 (49.5%)	143 (56.5%)	684 (58.7%)	
Duration of invasive ventilation	3 (1–7)	2 (1–6)	2 (1–6)	0.36
Vasopressor need	42 (45.2%)	130 (51.4%)	544 (46.7%)	
Duration of vasopressors	3 (1–5)	2 (1–4)	2 (1–4)	0.67
Dialysis	5 (5.4%)	21 (8.3%)	97 (8.3%)	
Duration of dialysis	3 (3–10)	5 (3–8)	3 (1–7)	0.26
Length of ICU stay	3.6 (1.6–7.7)	3.8 (1.9–8.0)	3.2 (1.6–7.9)	

A p-value of < 0.05 was considered statistically significant

a. Variable considered as categorical because of the limited number of unique values

**Table 2:** Cancer types

	Uncontrolled cancer (*n* = 117)	Controlled cancer (*n* = 270)
Solid malignancy		
Breast	9 (7.7%)	13 (4.8%)
Colon	11 (9.4%)	41 (15.2%)
Head and neck	8 (6.8%)	27 (10%)
Lung	11 (9.4%)	18 (6.7%)
Esophagus and stomach	13 (11.1%)	28 (10.4%)
Pancreas	1 (0.9%)	16 (5.9%)
Other	32 (27.4%)	76 (28.1%)
Hematological malignancy		
Acute leukemia	11 (9.4%)	21 (7.8%)
Lymphoma (Hodgkin, non-Hodgkin)	10 (8.6%)	18 (6.7%)
Other	15 (12.8%)	17 (6.3%)

6 patients had both acute leukemia and a solid malignancy (breast cancer, colon cancer and ‘other cancer’), 3 patients had both lymphoma and a solid malignancy (breast cancer, head and neck cancer and ‘other cancer’)

The difference in country, hospital, ICU and patient characteristics across subgroups are reported in Table 1. Patients with cancer (uncontrolled and controlled) were older than the patients without cancer (*p* = 0.002) and a significant difference in performance status was seen between cancer patients and patients without cancer (*p* < 0.001). Whereas cancer patients were more often admitted with sepsis or septic shock (*p* < 0.001), patients without cancer were more often admitted with heart failure (*p* < 0.001), neurologic pathology (*p* = 0.002) and trauma (multiple trauma *p* < 0.001, head trauma *p* = 0.003). A treatment limitation decision (i.e., no cardiopulmonary resuscitation or withholding other life-saving treatments) before ICU admission was more often observed in uncontrolled cancer patients than in controlled cancer patients and patients without cancer (18.8%, 5.9% and 5.2%, respectively; *p* < 0.001).

### Differences in outcomes across subgroups after adjustment for the case-mix, ICU, hospital and country characteristics

**Fig. 2:**
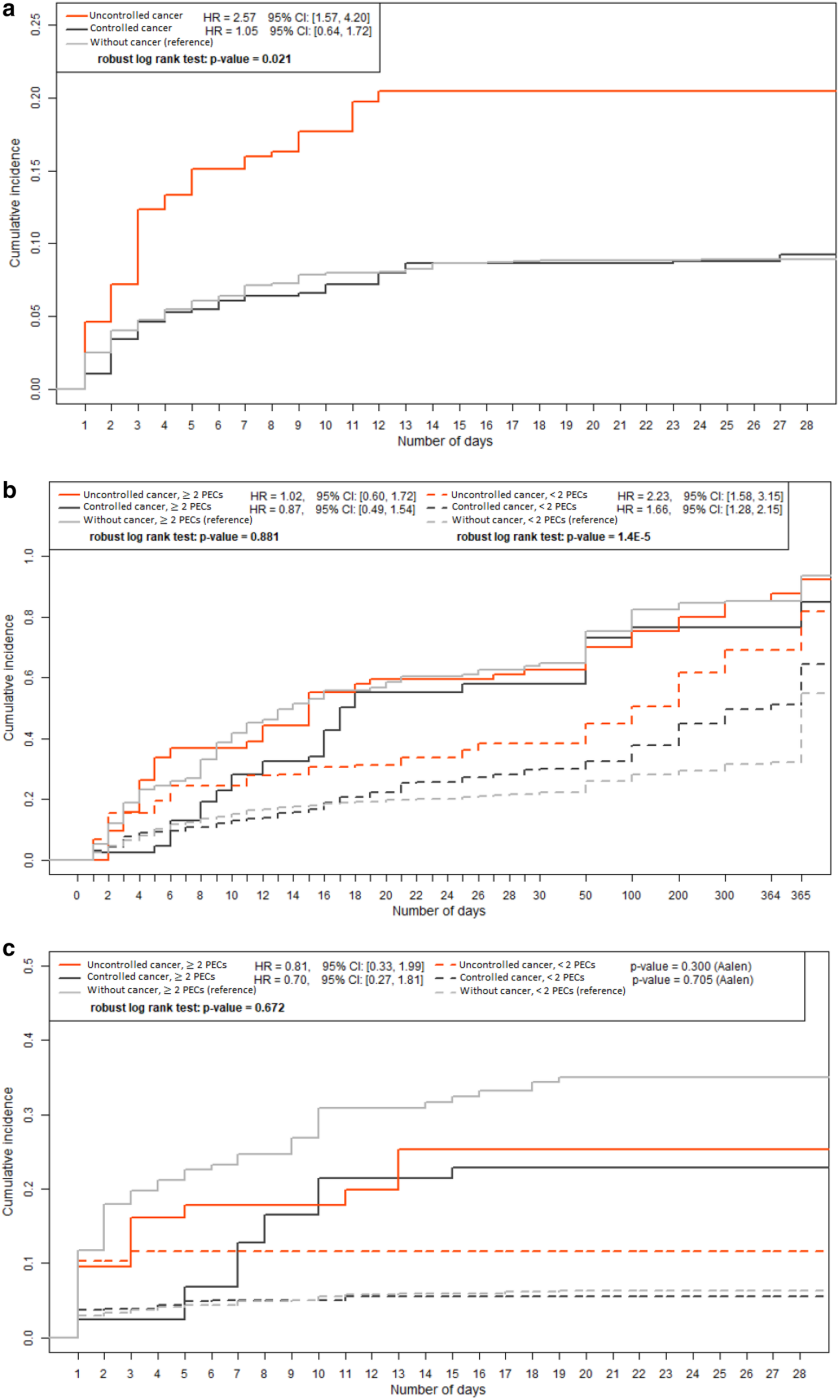
**a** Time from ICU admission until at least 2 PECs during ICU stay (weighted). **b** Time from ICU admission until death (weighted). **c** Time from ICU admission until TLD during ICU stay (weighted). *TLD* treatment limitation decision, *PEC* perceptions of excessive care

We found statistical evidence for a difference in cumulative incidence of concordant PECs between patients with uncontrolled cancer and patients without cancer (20.5% vs. 9.1%; *p* < 0.001). We found no evidence for such a difference between patients with controlled cancer and patients without cancer (8.1% vs. 9.1%; *p* = 0.62; Fig. 2a).

In patients with concordant PECs, we found no statistical evidence for a difference in time from admission until death between cancer patients and patients without cancer (uncontrolled cancer: HR 1.02, 95% CI 0.60–1.72 and controlled cancer HR 0.87, 95% CI 0.49–1.54; reference value: without cancer; Fig. 2b). Likewise, we found no evidence for a difference in TLDs. The HRs were 0.81 (95% CI 0.33–1.99) and 0.70 (95% CI 0.27–1.81; Fig. 2c).

In patients without concordant PECs, we found statistical evidence for a difference in time from admission until death between cancer patients and patients without cancer (uncontrolled cancer: HR 2.23, 95% CI 1.58–3.15 and controlled cancer HR 1.66, 95% CI 1.28–2.15; reference value: without cancer; Fig. 2b). We found no statistical evidence for a difference in TLDs (*p* = 0.71 and *p* = 0.3; Fig. 2c). This finding suggests prognostic optimism.

The risk of reaching the combined endpoint at 1 year in patients with concordant PECs was 93.7% in patients uncontrolled cancer, 82.5% in patients with controlled cancer, compared to 93.4% in patients without cancer (*p* = 0.99 and *p* ≤ 0.001, respectively), suggesting pessimistic prognostication in patients with controlled cancer by clinicians who provided PECs. The risk of reaching the combined endpoint at one year in patients without concordant PECs was 70.8% and 63.0% compared to 54.5% (*p* = 0.003 and *p* = 0.02, respectively; Table 3).

**Table 3:** Mortality and TLDs across subgroups (weighed results)

	Uncontrolled cancer (*n* = 117), %	Controlled cancer(*n* = 270), %	Without cancer (*n* = 1254), %	*p*-value
28-day mortality				
< 2 PECS	24.8	22.6	17	0.02
≥ 2 PECs	63	57.4	59.6	0.60
Treatment limitation decisions				
< 2 PECS	10.5	5.2	6.0	0.12
≥ 2 PECs	28.7	27.7	34.7	0.09
1-year mortality				
< 2 PECS	58.4	45.8	30.4	< 0.001
≥ 2 PECs	87.0	80.2	83.2	0.29
Combined endpoint				
< 2 PECS	70.8	63.0	54.5	0.001
≥ 2 PECs	93.7	82.5	93.4	< 0.001

*PEC* perceptions of excessive care, *TLD* treatment limitation decisions

Combined endpoint: death, poor quality of life or not being at home

Patients who were lost to follow-up are not included in this table

A *p*-value of < 0.05 was considered statistically significant

## Discussion

By combining subjective impressions of excessive care and objective patient data from the large multicentre DISPROPRICUS database, this is the first study, to our knowledge, which explores in more detail the issue of unconscious bias towards critically ill cancer patients by ICU clinicians. We found evidence of a difference in time from admission until death without a difference in time until written TLDs in patients without concordant PECs across subgroups. In patients with concordant PECs, only a difference in combined outcomes at one year was found.

As expected, patients with uncontrolled cancer had a higher risk of being identified by ICU clinicians as receiving excessive care than patients with controlled cancer and patients without cancer, even after adjusting for confounders. Although short-term outcomes after ICU admission in patients with uncontrolled cancer has been improving [1, 2, 17, 18], the presence of an underlying malignancy is independently associated with lower long-term survival [5, 19], justifying the higher incidence of PECs by ICU clinicians in this subgroup.

We found no evidence of a difference in time until death and written TLDs in patients with concordant PECs across subgroups in our study, suggesting that ICU clinicians who provided PECs do not discriminate between patient subgroups, once they have been identified as receiving excessive care. This observation, together with the higher incidence of concordant PECs in uncontrolled cancer patients, underpins the professionalism and moral engagement of these clinicians towards patients, relatives and society. However, patients with controlled cancer had a statistically significant lower risk of attaining the combined endpoint (death, poor quality of life or not being at home) at one year compared to the two other subgroups. This suggests some degree of prognostic pessimism, and as such the possibility of unconscious discrimination towards patients with controlled cancer, by ICU clinicians who provided PECs. Nevertheless, the overall poor outcomes at one year indicate that concordant PECs by ICU clinicians should be taken seriously by hematologists and oncologists and should be used to trigger multidisciplinary ethical reflection.

In contrast to patients with concordant PECs, we found evidence of differences between the time from admission until death at short-term and mid-term across subgroups in patients without concordant PECs, even after adjustment for baseline characteristics. This was also confirmed at long-term with the combined endpoint; patients with cancer were significantly more at risk to achieve the combined endpoint of death, not being at home or having a poor quality of life one year after ICU admission (Table 3). However, we found no evidence of a difference in written TLDs across these subgroups. Although we cannot completely rule out that this finding reflects the patients or family’s wishes, which were not take into account in this study, we would still have expected that clinicians would have provided PECs in such circumstances, since PECs were collected anonymously. Therefore, this finding suggests unduly prognostic optimism, uncertainty or unawareness about prognosis by ICU clinicians who did not provide PECs, more specifically in patients with uncontrolled cancer. Similarly to ICU clinicians, hematologists and oncologists may feel uncertain about the reliability of their prognostic estimation [20–24], perceive “death as a treatment failure” [20] or may feel guilty by having the impression that they are “giving a death sentence” by not starting or continuing advanced life-supporting therapy at the ICU [25]. Moreover, ICU clinicians are regularly confronted with patients and relatives who have unduly optimistic expectations [20], who are not always well informed by their hematologist or oncologist or who do not want to be fully informed [20], making shared decision-making about treatment options difficult. Fear of litigation and the huge availability of medical resources 24/7 in Western countries further increase the pressure to comply with the patient’s or family’s wishes in these circumstances [25]. This might explain why only 22% of ICU clinicians would refuse a patient’s request for non-beneficial treatment and that only 13% would not offer futile treatments [25].

Altogether, our results highlight the need to foster intradisciplinary and interdisciplinary ethical reflection to improve prognostication and subsequent decision-making for the benefit of the critically ill cancer patient. Besides reducing prognostic uncertainty [10], sharing knowledge, experience and values within a safe ethical climate may reduce the risk of discrimination of patients with controlled cancer and improve end-of-life decision-making in patients with uncontrolled cancer. Education about recent advances in cancer for ICU clinicians and about the limits and consequences of advanced ICU care for hematologists and oncologists could help in increasing mutual trust and quality of care. Formal training in ethics and palliative care has also been found to reduce uncertainty [26]. Furthermore, our results highlight the need for closed-loops systems in which both ICU clinicians and hematologists or oncologists learn from the results of ICU referrals. This can be obtained by organizing debriefing of complex patient cases, or by benchmarking patient outcomes and ethical decision-making climates across units between hospital [9, 10].

Our study has several limitations. First, the participating ICUs were not selected at random. This may have affected the external validity of our results. Second, inclusion of patients was left at the discretion of the attending doctors. However, we tried to reduce the risk of selection bias across ICUs by excluding the ethical climate with indication of selection bias in our main analysis [9] and by adjusting for country, hospital, ICUs and patients characteristics via propensity score weighing. Third, we cannot completely exclude a bias due to self-fulfilling prophecy. Self-fulfilling prophecy is a phenomenon in which predictions regarding prognosis by health care providers (unconsciously) change ICU treatment and therefore outcome of patients. By adjusting for the quality of the ethical decision-making climate at the ICU, we have tried to address the issue of self-fulfilling prophecy as much as possible by averaging patient outcomes between ICUs with a “good” climate (in which self-fulfilling prophecy could neither be excluded nor confirmed in the main analysis) and ICUs with a “poor climate” (in which decision-paralysis was observed in the main analysis [9]). Moreover, we found no evidence for a difference in concordant PECs by different combinations of clinicians with (doctor–doctor, doctor–nurse) and without decision-making power (nurse–nurse) across subgroups in this sub-analysis. Fourth, we used time until death and TLDs as surrogate markers of withholding or withdrawal of ICU treatment. We did not measure actual withholding or withdrawal of these treatments. Fifth, we did not use classical severity-of-illness scores in our analyses, as these scoring systems have not been validated for predicting long-term outcomes. We preferred to include short-term and long-term prognostic factors that are commonly used by clinicians during decision-making in daily practice [9]. Sixth, one has to keep in mind that the incidence of patients with concordant PECs is probably underestimated, as patients admitted prior to the study period were excluded from the analysis [9]. Finally, in the current study, no distinction was made between patients with a solid malignancy and patients with a hematological malignancy. However, this would not have altered our conclusion (see Additional file 2: master dissertation of E. Uyttersprot 16.

## Conclusions

The absence of a difference in time from admission until TLDs and death in patients with concordant PECs makes bias by ICU clinicians towards cancer patients unlikely. However, the differences between the time from admission until death, without a corresponding increase in time until TLDs, suggests prognostic unawareness, uncertainty or optimism in ICU clinicians who did not provide PECs, more specifically in patients with uncontrolled cancer. This study highlights the need to improve intra- and interdisciplinary ethical reflection and subsequent decision-making at the ICU.

## Supplementary Information

**Additional file 1.** Definitions of collected data.

**Additional file 2.** Master dissertation of E. Uyttersprot.

**Additional file 3: Fig. S1.** Flowchart study unweighted results: number of ICU’s, clinicians, perceptions and patients. PEC: Perceptions of Excessive Care. Combined endpoint: death, poor quality of life or not being at home.

**Additional file 4: Fig. S2.** a Time from ICU admission until at least 2 PECs during ICU stay (unweighted). b Time from ICU admission until death (unweighted). C Time from ICU admission until TLD during ICU stay (unweighted). TLD: treatment limitation decision, PEC: Perceptions of Excessive Care.

**Additional file 5: Table S3.** Mortality and TLDs across subgroups (unweighted results)

## Data Availability

The data that support the findings of this study are available from the corresponding author (DB) upon reasonable request.

## Abbreviations

ECOG performance status: Eastern Cooperative Oncology Group performance status
EDMCQ: Ethical Decision-Making Climate Questionnaire
HR: Hazard ratios
ICU: Intensive Care Unit
PEC: Perception of excessive care
TLD: Treatment limitation decisions
95% CI: 95% Confidence intervals

## References

[1] Azoulay E, Schellongowski P, Darmon M, Bauer PR, Benoit D, Depuydt P (2017). The Intensive Care Medicine research agenda on critically ill oncology and hematology patients. Intensive Care Med.

[2] Bos MM, Verburg IW, Dumaij I, Stouthard J, Nortier JW, Richel D (2015). Intensive care admission of cancer patients: a comparative analysis. Cancer Med.

[3] Puxty K, McLoone P, Quasim T, Sloan B, Kinsella J, Morrison DS (2015). Risk of Critical Illness Among Patients With Solid Cancers: A Population-Based Observational Study. JAMA Oncol.

[4] Jacobson D, Chiu N, Cheung MC, Fowler R, Buckstein R (2016). Predictors for ICU admission and clinical outcomes of malignant hematology patients admitted to critical care units. Blood..

[5] Puxty K, McLoone P, Quasim T, Sloan B, Kinsella J, Morrison DS (2018). Characteristics and outcomes of surgical patients with solid cancers admitted to the intensive care unit. JAMA Surg.

[6] Soares M, Caruso P, Silva E, Teles JM, Lobo SM, Friedman G (2010). Characteristics and outcomes of patients with cancer requiring admission to intensive care units: a prospective multicenter study. Crit Care Med.

[7] Taccone FS, Artigas AA, Sprung CL, Moreno R, Sakr Y, Vincent JL (2009). Characteristics and outcomes of cancer patients in European ICUs. Crit Care.

[8] Benoit DD, Soares M, Azoulay E (2014). Has survival increased in cancer patients admitted to the ICU? We are not sure. Intensive Care Med.

[9] Benoit DD, Jensen HI, Malmgren J, Metaxa V, Reyners AK, Darmon M (2018). Outcome in patients perceived as receiving excessive care across different ethical climates: a prospective study in 68 intensive care units in Europe and the USA. Intensive Care Med.

[10] Van den Bulcke B, Piers R, Jensen HI, Malmgren J, Metaxa V, Reyners AK (2018). Ethical decision-making climate in the ICU: theoretical framework and validation of a self-assessment tool. BMJ Qual Saf.

[11] Detsky ME, Harhay MO, Bayard DF, Delman AM, Buehler AE, Kent SA (2017). Discriminative accuracy of physician and nurse predictions for survival and functional outcomes 6 months after an ICU admission. JAMA.

[12] Meadow W, Pohlman A, Reynolds D, Rand L, Correia C, Christoph E (2014). Power and limitations of daily prognostications of death in the medical ICU for outcomes in the following 6 months. Crit Care Med.

[13] Neville TH, Wiley JF, Yamamoto MC, Flitcraft M, Anderson B, Curtis JR (2015). Concordance of nurses and physicians on whether critical care patients are receiving futile treatment. Am J Crit Care.

[14] Singal RK, Sibbald R, Morgan B, Quinlan M, Parry N, Radford M (2014). A prospective determination of the incidence of perceived inappropriate care in critically ill patients. Can Respir J.

[15] Rubin DB (1997). Estimating causal effects from large data sets using propensity scores. Ann Intern Med.

[16] Uyttersprot E. Incidence and predictive value of clinicians’ perceptions of excessive care in different ICU subpopulations. Do ICU-clinicians stigmatize specific patient populations? 2018–2019.

[17] Darmon M, Bourmaud A, Georges Q, Soares M, Jeon K, Oeyen S (2019). Changes in critically ill cancer patients' short-term outcome over the last decades: results of systematic review with meta-analysis on individual data. Intensive Care Med.

[18] Ostermann M, Ferrando-Vivas P, Gore C, Power S, Harrison D (2017). Characteristics and outcome of cancer patients admitted to the ICU in England, Wales, and Northern Ireland and National Trends between 1997 and 2013. Crit Care Med.

[19] Sauer CM, Dong J, Celi LA, Ramazzotti D (2019). Improved survival of cancer patients admitted to the intensive care unit between 2002 and 2011 at a US Teaching Hospital. Cancer Res Treat..

[20] Back AL, Anderson WG, Bunch L, Marr LA, Wallace JA, Yang HB (2008). Communication about cancer near the end of life. Cancer.

[21] Lamont EB, Christakis NA (2001). Prognostic disclosure to patients with cancer near the end of life. Ann Intern Med.

[22] Kimbell B, Murray SA, Macpherson S, Boyd K (2016). Embracing inherent uncertainty in advanced illness. BMJ..

[23] Palda VA, Bowman KW, McLean RF, Chapman MG (2005). "Futile" care: do we provide it? Why? A semistructured, Canada-wide survey of intensive care unit doctors and nurses. J Crit Care.

[24] Piers RD, Azoulay E, Ricou B, DeKeyser GF, Max A, Michalsen A (2014). Inappropriate care in European ICUs: confronting views from nurses and junior and senior physicians. Chest.

[25] Fetters MD, Churchill L, Danis M (2001). Conflict resolution at the end of life. Crit Care Med.

[26] Darmon M, Ducos G, Coquet I, Resche-Rigon M, Pochard F, Paries M (2016). Formal academic training on ethics may address junior physicians' needs. Chest.

