# Supplementary material for: Outcomes of ICU patients with and without perceptions of excessive care: a comparison between cancer and non-cancer patients
Source: Ann Intensive Care. 2021 Jul 31;11:120. doi: 10.1186/s13613-021-00895-5 (PMC8325749; doi:10.1186/s13613-021-00895-5)
Supplement: Supplementary file 1 — Additional file 1. Definitions of collected data. [file 13613_2021_895_MOESM1_ESM.docx]

Table S 1 : Definitions of collected data

| **Country Characteristics** | **Definition** |
| --- | --- |
| Number of ICU beds / 100.000 inhabitants | The number of ICU beds per 100.000 inhabitants |
| Geographical region  Central Europe  Northern Europe  Southern Europe  Western Europe / USA | The number of patients admitted to an ICU in a country in Central Europe, Northern Europe, Southern Europe and Western Europe / USA |
| **Hospital characteristics** |  |
| Hospital type  Public  Private  University-affiliated  University | The number of patients admitted to an ICU in a public hospital, in a private hospital, in a university-affiliated hospital or a university hospital. |
| Total beds in hospital  < 250  250-499  500-749  > 750 | The number of patients admitted to an ICU in a relatively small hospital, in a medium small hospital, in a medium large hospital and a large hospital |
| **ICU characteristics** |  |
| Ethical climate  Good  Average +  Poor | The number of patients admitted to an ICU with a good ethical climate, an average ethical climate or a poor ethical climate |
| Number of beds per ICU | Number of ICU beds per ICU |
| Percentage of population > 65 year in ICU | Number of patients older than 65 year admitted to the ICU |
| Patient to nurse ratio | The number of patients per nurse |
| Patient to junior physician ratio | The number of patients per junior physician |
| Patient to senior physician ratio | The number of patients per senior physician |
| **Patient characteristics** |  |
| Age | Age in years (continuous) and patients 75 year or older (categorical) |
| Gender | Male |
| ECOG performance status  Grade 0  Grade 1  Grade 2  Grade 3  Grade 4  Unknown | Eastern Cooperative Oncology Group Performance Status 14 days before ICU admission  Fully active, able to carry on all pre-disease performance without restriction  Restricted in physically strenuous activity but ambulatory and able to carry out work of a light or sedentary nature, e.g., light house work, office work  Ambulatory and capable of all self-care but unable to carry out any work activities; up and about more than 50% of waking hours  Capable of only limited self-care; confined to bed or chair more than 50% of waking hours  Completely disabled; cannot carry on any self-care; totally confined to bed or chair  Unknown ECOG performance status |
| Nursing home resident | Number of patients living in a nursing home |
| Moderate to severe comorbidities  0  1  ≥ 2 | Number of patients without comorbidity (as defined below), with one comorbidity (as defined below) or with two or more comorbidities (as defined below) |
| Type comorbidity  Heart failure (NYHA III or IV)  COPD^a^ (Gold III or IV or equivalent)  Neurological (excluding dementia)  Liver cirrhosis (Child Pugh B or C)  Chronic renal failure requiring dialysis  Dementia (moderate or severe) ^b^  AIDS | Number of patients with heart failure, COPD, neurological comorbidity, liver cirrhosis, renal failure requiring dialysis, dementia and AIDS. |
| Abuse  Alcohol^c^  Smoking | Number of patients who drink or smoke |
| Main admission reason  Respiratory failure  Sepsis/severe sepsis/ septic shock  Heart failure / cardiogenic shock  Neurologic pathology / stroke/ ICB  Gastro-intestinal pathology / liver failure  Metabolic / renal  Multiple trauma  Head trauma | Number of patients with respiratory failure, sepsis, heart failure, neurologic pathology, gastro-intestinal pathology, metabolic or renal disease, multiple trauma or head trauma as admission reason |
| Surgery within 48 hrs | Number of patients with surgery within 48 hrs of ICU admission |
| Surgery  No surgery  Scheduled surgery  Unscheduled surgery | Number of patients with no surgery, scheduled surgery and unscheduled surgery |
| Do-not-resuscitate order before ICU admission  Full code  Unknown  No CPR  Withholding therapy | Number of patients with a do-not-resuscitate order before ICU admission |
| Severity of illness < 24 hrs after admission  Invasive mechanical ventilation  Vasopressor need  Dialysis | Number of patients in need of invasive mechanical ventilation, vasopressors or dialysis |
| Written withholding / withdrawing order < 24 h | Number of patients with a written treatment limitation decision within 24 hrs of ICU admission |
| Characteristics during ICU stay  Invasive mechanical ventilation  Duration of invasive ventilation  Vasopressor need  Duration of vasopressors  Dialysis  Duration of dialysis | Number of patients in need of invasive mechanical ventilation, vasopressors or dialysis and duration in days of these treatments |

NYHA : New York Heart Association.

COPD : Chronic Obstructive Pulmonary Diseases.

AIDS : Acute Immune Deficiency Syndrome.

ICB : Intra-Cranial Bleeding.

CPR : Cardio-Pulmonary Resuscitation.

a Similar stage according to other definitions in absence of pulmonary function test or chronic oxygen therapy.

b Global Deterioration Scale 6 (largely unaware of recent experiences and events in their lives. require assistance with basic ADL’s. behavioral and psychological symptoms of dementia are common) or 7 (verbal abilities will be lost over the course of this stage, incontinent, needs assistance with feeding, lose ability to walk).

c More than 4 drinks a day for male, more than 3 drinks a day for female
