# Supplementary material for: Outcomes of ICU patients with and without perceptions of excessive care: a comparison between cancer and non-cancer patients
Source: Ann Intensive Care. 2021 Jul 31;11:120. doi: 10.1186/s13613-021-00895-5 (PMC8325749; doi:10.1186/s13613-021-00895-5)
Supplement: Supplementary file 2 — Additional file 2. Master dissertation of E. Uyttersprot. [file 13613_2021_895_MOESM2_ESM.pdf]

GHENT UNIVERSITY

FACULTY OF SCIENCES

Incidence and predictive value of clinicians' perceptions of excessive care in different ICU subpopulations.

Do ICU-clinicians stigmatize specific patient populations?

Emma Uyttersprot

Master dissertation submitted to obtain the degree of Master of Statistical Data Analysis

Promoter: Prof. Dr. Stijn Vansteelandt

Co-promoters: Prof. Dr. Dominique Benoit

Prof. Dr. Ruth Piers

Department of Applied Mathematics, Computer Science and Statistics

Academic year 2018 - 2019

GHENT UNIVERSITY

FACULTY OF SCIENCES

Incidence and predictive value of clinicians' perceptions of excessive care in different ICU subpopulations.

Do ICU-clinicians stigmatize specific patient populations?

Emma Uyttersprot

Master dissertation submitted to obtain the degree of Master of Statistical Data Analysis

Promoter: Prof. Dr. Stijn Vansteelandt

Co-promoters: Prof. Dr. Dominique Benoit

Prof. Dr. Ruth Piers

Department of Applied Mathematics, Computer Science and Statistics

Academic year 2018 - 2019

The author and the promoter give permission to consult this master dissertation and to copy it or parts of it for personal use. Each other use falls under the restrictions of the copyright, in particular concerning the obligation to mention explicitly the source when using results of this master dissertation.

Author,	Promotor,	Co-promotor,	Co-promotor,
E. Uyttersprot	S. Vansteelandt	D. Benoit	R. Piers

Foreword

I am very proud to present to you my thesis as the final step in the MaStat program. The last year and a half was very challenging, but I have learned so much. Not only were the courses very interesting, but the teachers were really motivated and always open to discussion and questions as well. It was also very enriching to meet so many students with a different cultural and/or educational background.

I would like to thank my thesis promotor Stijn Vansteelandt for providing me this thesis topic, for taking the time to answer all my questions and for giving feedback. I would also like to express my gratitude to my co-promotors Ruth Piers and Dominique Benoit from UZGent for the pleasant collaboration and for giving me more insight into the medical part of this thesis topic. Lastly, a very big thank you to my family and friends for supporting me throughout this entire "MaStat-journey".

Table of contents

Foreword **I**

Abstract **V**

1 Introduction **1**

2 Description of the dataset **3**

2.1 How was the dataset obtained? 3

2.2 Some important variables 4

3 Methods **5**

3.1 Survival analysis 5

3.1.1 Time, event, censoring and censoring assumptions 5

3.1.2 Survivor function and hazard function 6

3.1.3 Cox proportional hazard model 7

3.1.4 Dealing with competing risks 12

3.1.5 Log rank test 14

3.1.6 Application in study 15

3.2 Inverse propensity score weighting 17

3.2.1 Experimental data versus observational data 17

3.2.2 Propensity score 17

3.2.3 Inverse propensity score weighting 18

3.2.4 Disadvantages of inverse propensity score weighting 19

4 Discrimination **20**

4.1 Indications of discrimination 20

4.2 How to detect indications of discrimination? 21

5 Univariate description: differences between subgroups **23**

5.1 Studied subgroups 23

5.2 Differences between age subgroups 23

5.3 Differences between cancer type subgroups 24

5.4 Differences between cancer status subgroups 25

5.5 Differences between surgery subgroups 26

6 Risk of death within 28 days, death within one year, CEP and TLD 27
6.1 Goal and method 27
6.2 Risk of death within 28 days 27
6.3 Risk of death within 1 year 28
6.4 Risk of combined endpoint 29
6.5 Risk of TLD 29
6.6 Remark about patients without concordant PECs 30
7 Unweighted cumulative incidence curves 31
7.1 Age subgroups 31
7.2 Cancer type subgroups 32
7.3 Cancer status subgroups 33
7.4 Surgery subgroups 35
7.5 First indication of discrimination? 36
7.6 Difference in mortality rate between subgroups 38
7.6.1 Goal and method 38
7.6.2 Age subgroups 38
7.6.3 Cancer type subgroups 39
7.6.4 Cancer status subgroups 40
7.6.5 Surgery subgroups 40
8 Inverse propensity score weighting 42
8.1 Goal and method 42
8.2 Variables related with CEP 42
8.3 Variables related with age 43
8.4 Propensity scores and weights for age 44
8.5 Propensity scores and weights for cancer type, cancer status and surgery 45
9 Weighted cumulative incidence curves 46
9.1 Calculation of cumulative incidence 46
9.2 Age subgroups 46
9.3 Cancer type subgroups 47
9.4 Cancer status subgroups 49

9.5 Surgery subgroups 50

9.6 First indication of discrimination? 51

9.7 Difference in mortality rate between subgroups 52

9.7.1 Age subgroups 52

9.7.2 Cancer type subgroups 52

9.7.3 Cancer status subgroups 53

9.7.4 Surgery subgroups 54

10 Conclusion 55

11 References 58

A Appendix

Abstract

Life supporting therapy should only be provided to patients in an intensive care unit (ICU) if the patients and their relatives are well informed about the treatment and associated risks and if the treatment intensity is proportional to the expected result. However, Teno J.M. et al. (2013) [1] showed that 1 in 3 patients die shortly after treatment at ICU, which may be an indication that the level of care is not appropriate. A possible measure of overtreatment is the number of perceptions of excessive care (PECs) that patients receive from clinicians.

For this thesis, data from the 28-day observational study (multicenter DISPROPRICUS study) has been used. The dataset contains patient, ICU, country, clinician and hospital characteristics. Different subgroups of patients have been studied: age subgroups (< 75 year old, ≥ 75 year old), cancer type subgroups (no cancer, hematological cancer, solid tumor), cancer status subgroups (no cancer, active cancer, not active cancer) and surgery subgroups (no surgery, scheduled surgery, unscheduled surgery). Univariate analysis showed that the proportion of patients with concordant PECs is significantly higher for older patients, for patients with hematological cancer and for patients with active cancer in comparison with the other subgroups of patients.

The main objective of this thesis was to study whether it is possible that clinicians discriminate certain subgroups of patients. Discrimination is possible if 1) there is a significant difference in the proportion of patients with concordant PECs or in the rate of receiving those concordant PECs and 2) if there is a significant difference in the proportion of patients with a treatment limitation decision (TLD) registration or in the rate of TLD registration between the different subgroups.

In order to evaluate whether discrimination of patients may occur, cumulative incidence curves have been constructed for all subgroups for 1) the time from admission until receiving the 2nd PEC and 2) the time from receiving the 2nd PEC until TLD registration. By fitting cause-specific hazard models, hazard rates for different subgroups could be compared. Cumulative incidence curves for the time from receiving the 2nd PEC until death or combined endpoint have been studied as well (although no extra information about possible discrimination can be obtained from these). To adjust for background characteristics, inverse propensity score weighting has been applied and weighted cumulative incidence curves were constructed. The propensity score is defined as the estimated conditional probability for a patient to belong to his own subgroup given the patient's characteristics.

The results based on the unweighted and weighted cumulative incidence curves are almost identical. No significant difference in cause-specific hazard rate of TLD registration was detected between any of the subgroups. The cause-specific hazard rate of receiving concordant PECs was significantly higher for older patients and for patients with hematological cancer in comparison with the patients in the other subgroups. No significant difference in cause-specific hazard rate of receiving concordant PECs between the surgery subgroups was detected. Based on the unweighted cumulative incidence curves, the cause-specific hazard rate of receiving concordant PECs was significantly higher for patients with active cancer than for patients with not active cancer or patients without cancer. However, this difference was not detected based on the weighted cumulative incidence curves. Overall, it could be concluded that discrimination of patients based on age, cancer type, cancer status or surgery type by clinicians doesn't seem plausible.

1 Introduction

An intensive care unit (ICU) is a department in a hospital that is specialised in treating patients with life-threatening conditions. These are, for example, patients that went through major surgery, had a severe accident, have cardiac problems, severe pneumonia or sepsis, are in a coma, are paralyzed, etc. In order to be able to treat these patients, well-educated and trained doctors and nurses are needed.

Life-supporting therapy should only be provided to a patient when the following two conditions are met. Firstly, the patient and his relatives should be well informed about the treatment and the associated risks and the level of care must be in accordance with the patient's wishes and preferences. Secondly, the treatment intensity should remain proportional to the expected result. However, Teno J.M. et al. (2013) [1] showed that 1 in 3 patients die shortly after ICU treatment, which raises the question if the level of care given to patients is too high (overtreatment) or too low (undertreatment).

In this study, the focus lies on possible overtreatment of patients. Several risks are associated with overtreatment. Firstly, if too much care is given, it is possible that unnecessary suffering is added to the patient and the family members. Secondly, healthcare providers also want the level of care they are providing to be appropriate. If they see that their patients die during the ICU-stay or shortly after, they may feel like they have failed and their motivation may decrease which may eventually lead to burn-outs. Thirdly, as ICUs are costly because of high technology and highly specialized personnel, it is important that the units work efficiently. Deciding how much money should be invested in the last years of life, knowing that the costs increase as the duration of care increases, is a difficult ethical issue.

An indication of possible overtreatment of patients, is the number of perceptions of excessive care (PECs) they receive from the clinicians. The multicenter study by D.D. Benoit et al. (2018) showed that the probability that a patient is dead, not at home or has a poor quality of life, one year after ICU admission, is much higher when the patient received a PEC by two or more clinicians independent from each other (concordant PECs) in comparison with a patient who didn't receive two or more PECs [2].

For this study, several subgroups of patients are considered: age subgroups (< 75 year old, ≥ 75 year old), cancer type subgroups (no cancer, hematological cancer, solid tumor), cancer status subgroups (no cancer, active cancer, not active cancer) and surgery subgroups (no surgery, scheduled surgery, unscheduled surgery). The goal of this study is to look for hints of discrimination of the patients based on age, cancer status, cancer type or surgical status by the clinicians. For example: do clinicians give up on older patients or patients with cancer more quickly than on younger patients or patients without cancer, when other factors related to a bad outcome are equal? Is there a difference in time until treatment limitation decision (TLD) registration?

In order to be able to answer all these questions, the following items were studied for all subgroups: 1) the number of received PECs, 2) the predictive value of PECs with regard to the patient's condition after one year, 3) cumulative incidence curves (for the time from admission until the 2nd PEC, for the time between the 2nd PEC and death or combined endpoint and for the time between the 2nd PEC and TLD) and 4) the (cause-specific) hazard rates of receiving concordant PECs, of TLD registration and of dying. To adjust for background characteristics, inverse propensity score weighting was applied and weighted cumulative incidence curves could be constructed. The propensity score is defined as the estimated conditional probability for a patient to belong to his own subgroup given the patient's characteristics.

This thesis has been split up in 8 parts. A description of the dataset is given in chapter 2. The theoretical background of the applied methods has been discussed in chapter 3. Chapter 4 explains how discrimination is defined and how it can be evaluated based on the available data. A univariate description of the data has been presented in chapter 5. The risk of death within 28 days, the risk of death within 1 year, the risk of reaching the combined endpoint and the risk of TLD for the different subgroups has been discussed in chapter 6. Finally, the cumulative incidence curves, the weighting process and the weighted cumulative incidence curves have been discussed in chapters 7, 8 and 9.

Figures and tables shown in the thesis, are referred to as "Figure x" and "Table y". If the tables and figures are shown in the appendix, they are referred to as "App Figure x" and "App Table y". All analyses in this thesis have been done using R version 3.5.0.

2 Description of the dataset

2.1 How was the dataset obtained?

A 28-day observational study was executed in 68 ICUs spread across the USA and 12 European countries (Belgium, Czech Republic, Denmark, France, Germany, Greece, Hungary, Italy, Portugal, Sweden, The Netherlands, United Kingdom). During those 28 days, clinicians working at the ICUs completed a daily anonymous questionnaire about their perception of disproportionate care for each of their patients. Disproportionate care was defined as care that is provided against the patient's wishes or that is no longer consistent with the expected survival or the expected quality of life. Many other patient characteristics were registered as well: age, gender, ECOG performance status, number and type of comorbidities, main admission reasons, ICU mortality, length of stay at the ICU, if the patient had a drinking or drug problem, presence of a do-not-resuscitate (DNR) order before admission and if a TLD was officialized or not.

One year after the ICU stay, an interviewer collected information about the vital status, place of residence and quality of life of the patients. If a patient was either dead, didn't live at home anymore or had a poor quality of life after one year, then it was said that the patient has reached the combined endpoint (CEP). If the patient was still alive, at home and had a good quality of life, the combined endpoint wasn't reached. This variable, the combined endpoint, is the primary outcome variable for this study.

Besides patient characteristics, the dataset also contains ICU, hospital, clinician and country characteristics. One of the ICU characteristics is the ethical work climate in the ICU. There are four types of ethical climates: a good climate, an average$^{(+)}$ climate (with involvement of clinicians at the end of life), an average$^{(-)}$ climate (without involvement of clinicians at the end of life) and a poor climate. The better the ethical climate, the more comfortable the clinicians feel about giving their opinion to colleagues and superiors and the more their opinion is valued and also integrated into decision making processes.

The multicenter study by D.D. Benoit et al. (2018) [2], which was based on the same data as the current study, showed that the ICU mortality and length of stay in the average$^{(-)}$ climate differs from the other climates. It was concluded that the attending physicians in average$^{(-)}$ climates included patients in the study in a dissimilar way to physicians in good, average$^{(+)}$ and poor climates and that selection bias was therefore present (see App Table 1). In order to avoid problems due to this selection bias, it was decided for the current study to exclude all patients that were admitted at an ICU with an average$^{(-)}$ ethical climate (120) and to do the study based on the available information of the remaining 1641 patients.

2.2 Some important variables

In order to get a better feel of the data, some important variables will be discussed here. A first series of important variables are the ones that are responsible for dividing the 1641 patients in different subgroups:

- a variable with age group indication: < 75 year, ≥ 75 year
- a variable with cancer type group indication: no cancer, solid tumor, hematological cancer
- a variable with cancer status indication: no cancer, active cancer, not active cancer
- a variable with surgery type indication: no surgery, scheduled surgery, unscheduled surgery

When a patient is in a really bad condition, it is possible that doctors decide to stop or to limit treatment. The dataset contains a binary variable with value 1 if a TLD has been registered and 0 otherwise.

In this study, a distinction was made between patients who did or did not receive a PEC by two or more clinicians independently from each other (concordant PECs). Therefore a binary variable has been created with value 1 if the patient has received concordant PECs and 0 otherwise.

A final series of variables indicate a time period: time from admission until receiving the 2nd PEC, time between receiving the 2nd PEC and death or CEP and time between receiving the 2nd PEC and TLD. Those variables will be used to construct cumulative incidence curves. The dataset contains many more variables, but they will not be discussed in further detail here.

3 Methods

3.1 Survival analysis

3.1.1 Time, event, censoring and censoring assumptions

Survival analysis is the analysis of data for which the outcome variable of interest is the time until a certain event occurs (also called "the survival time"). Very often, survival analysis has to take censoring into account. Censoring occurs when there is some information about an individual's survival time, but the exact survival time is unknown. In most cases, right-censoring will be present: the observed survival time is shorter than the true survival time (see Figure 1) . There are generally three reasons why right-censoring occur:

- The individual does not experience the event before the end of the study period.
- An individual is lost to follow up during the study period.
- An individual withdraws from the study before the end of the study period.

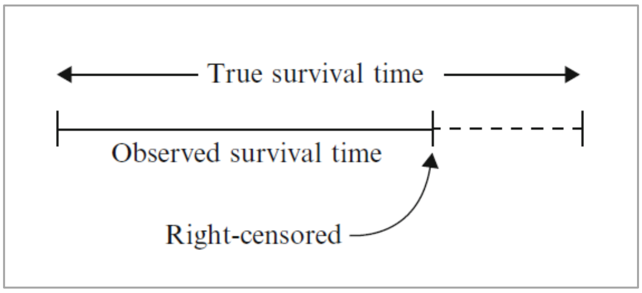

Figure 1: Schematic presentation of right censoring [3].

Often occurring assumptions about censoring are random censoring and independent censoring:

- Random censoring means that the individuals who are censored at time t are representative for the individuals who remained at risk at time t with respect to their survival time. Subjects at risk are subjects who have not experienced an event (yet) and who are not censored (yet). Assuming random censoring therefore means assuming that the event rate at time t is equal to the event rate at time t given that censoring has not occurred yet [4].

- Independent censoring means that, within any subgroup of interest, the individuals who are censored at time t are representative for the individuals in that subgroup who remained at risk at time t with respect to their survival time. Independent censoring is actually equal to random censoring within any subgroup of interest [3].

3.1.2 Survivor function and hazard function

The random variable for an individual's time-to-event is denoted by T. An observed value or a value of interest for the time-to-event is denoted by t. Two possible ways to describe time-to-event data are the survivor function S(t), with focus on the event not happening and the hazard function h(t), with focus on the event happening.

The survivor function gives the probability that an individual doesn't experience the event before a specific time t and is described as:

$$S(t) = P(T > t)$$

In this study however, the focus lies on the hazard function which is described as:

$$h(t) = \lim_{\Delta t \to 0} \left(\frac{P\left[t \le T \le t + \Delta t \mid T \ge t \right]}{\Delta t} \right)$$

The hazard function, also called the conditional event rate, gives the instantaneous probability for the event occurring at time t, per time unit, given that the event didn't occur before time t [3].

3.1.3 Cox proportional hazard model

a) <u>The model</u>

The hazard function can also be described by making use of the Cox proportional hazard model:

$$h(t|\,x) = h_0(t)\,.\,\exp\left(\beta_1\,.\,x_1 + \beta_2\,.\,x_2 + \ldots + \beta_p\,.\,x_p\right) = h_0(t)\,.\,\exp\left(\sum_{j=1}^{p}\beta_j\,.\,x_j\right)$$

with:	t	=	time
	h(t)	=	hazard function
	$h_0(t)$	=	baseline hazard
	x_j	=	covariate
	β_j	=	regression coefficient (measures the impact of covariate j)
	p	=	total number of covariates in the model

If all x's are zero (or if no x's are present), the hazard rate equals the baseline hazard: $h(t|\,x) = h_0(t)\,.\,e^0 = h_0(t)$. This baseline hazard is a function of the time t. The Cox proportional hazard model is a semi-parametric model as it doesn't make any assumptions about the form of the baseline hazard function but it does assume a parametric form for the effect of the predictors on the hazard (they enter the model linearly at the logscale and the coefficients are independent from the time). The Cox proportional hazard model can be fitted to the data and coefficient estimates can be obtained by maximizing the partial likelihood function. The partial likelihood function is described as follows:

$$L = \prod_{j=1}^{k} L_j$$

with:	L	=	likelihood function
	L_j	=	likelihood of experiencing the event at the jth event time given the event didn't occur before
	k	=	total number of observed event times

This partial likelihood function only takes the likelihoods for the observed event times into consideration. These depend however on the number of subjects at risk, which will decrease as time goes by (subjects who experience the event or subjects who are censored are no longer at risk).

The advantage of the semi-parametric model is that no (possibly wrong) assumption about the form of the baseline hazard needs to be made. The disadvantage however is that the resulting estimates may not be as efficient as the maximum likelihood estimates when a complete parametric model would be used [3].

b) Hazard ratio and proportional hazard assumption

In order to compare the hazard rate or conditional event rate between two subjects A and B with $x^A = \left(x_1^A, x_2^A, \ldots, x_p^A\right)$ and $x^B = \left(x_1^B, x_2^B, \ldots, x_p^B\right)$ respectively as sets of predictors, the hazard ratio between these two subjects can be estimated as follows:

$$\hat{HR}_{A/B} = \frac{\hat{h}\left(t \mid x^A\right)}{\hat{h}\left(t \mid x^B\right)} = \frac{h_0(t)\,.\,\exp\left(\sum_{j=1}^{p} \hat{\beta}_j\,.\,x_j^A\right)}{h_0(t)\,.\,\exp\left(\sum_{j=1}^{p} \hat{\beta}_j\,.\,x_j^B\right)} = \exp\left(\sum_{j=1}^{p} \hat{\beta}_j\,.\left(x_j^A - x_j^B\right)\right)$$

As can be seen in the formula above, the baseline hazard is cancelled out and the estimated hazard ratio $\hat{HR}_{A/B}$ is not a function of the time t. The hazard rate for subject A is a constant multiple of the hazard rate for subject B.

If $\hat{HR}_{A/B} > 1$: hazard rate for subject A is larger than for subject B.

If $\hat{HR}_{A/B} = 1$: hazard rate for both subjects is the same.

If $\hat{HR}_{A/B} < 1$: hazard rate for subject B is larger than for subject A.

The hazard rates for both subjects can change as times goes by, but the ratio is constant. Using the Cox model therefore always implies the assumption of proportional hazards.

Imagine only one exposure variable (with value 0 for no exposure and value 1 for exposure) is present in the Cox model. The hazard ratio between an exposed subject A and an unexposed subject B can then be estimated as follows:

$$\hat{HR}_{A/B} = \frac{\hat{h}\left(t \mid x^A\right)}{\hat{h}\left(t \mid x^B\right)} = \frac{h_0(t) \, . \, \exp\left(\hat{\beta}_1 . 1\right)}{h_0(t) \, . \, \exp\left(\hat{\beta}_1 . 0\right)} = \exp\left(\hat{\beta}_1\right)$$

The hazard ratio is then the exponential of the estimated regression coefficient $\hat{\beta}_1$. To test whether there is a significant difference in hazard rates between the two individuals, a Wald test can be executed:

$$H_0: \quad \beta_1 = 0 \quad \left(equivalent \ to \ HR = 1\right)$$
$$H_a: \quad \beta_1 \neq 0 \quad \left(equivalent \ to \ HR \neq 1\right)$$

$$Test \ statistic: \qquad z_W = \frac{\hat{\beta}_1}{SE\left(\hat{\beta}_1\right)}$$

If the null hypothesis can be rejected, it can be concluded that β_1 is significantly different from 0, the hazard ratio is significantly different from 1 and that there is a significant difference between the hazard rates of the two subjects. A log rank test can also be used to compare hazard rates (see section 3.1.5).

A Cox proportional hazard model can be fitted to the data using the function *"coxph()"* from the package "*Survival*" in R. With this function, the coefficient estimates, their standard errors, the results of the Wald tests and the result of the log rank test can be obtained [3] [5] [6].

c) <u>Assessing proportional hazard assumption – scaled Schoenfeld residuals</u>

If the proportional hazard assumption for the Cox model is not met, the coefficient estimates may be biased. Therefore, it is important to check whether the assumption is reasonable before using the model. This can be done by analysing the scaled Schoenfeld residuals for each covariate. The scaled Schoenfeld residuals at time t for covariate j can be calculated as follows:

1. Calculate for each individual i the difference between the covariate value x_{ij} and the weighted mean of the covariate values for all individuals at risk at time t. Individuals at risk at time t are the individuals who have not experienced the event yet and who are not censored (yet) at time t.

2. The Schoenfeld residuals, for each covariate j, are obtained by summing these differences over the subjects who experience the event at time t

3. The scaled Schoenfeld residuals s_j are obtained by dividing the Schoenfeld residuals by the variance of the estimated regression coefficient β_j.

These scaled Schoenfeld residuals are calculated for every coefficient β_j at every time t. Grambsch and Therneau (1994) showed that $E(s_j) + \hat{\beta}_j \approx \beta_j(t)$. The proportional hazard assumption implies that the regression coefficients β_j don't vary in function of the time. This implies on its turn that $\hat{\beta}_j = \beta_j(t)$ and that the expected value of the scaled Schoenfeld residuals $E(s_j)$ equals to zero. This is the case if there is no significant relationship between the scaled Schoenfeld residuals and time.

Assessment of the proportional hazard assumption can therefore be done by testing if there is a significant relationship between the scaled Schoenfeld residuals and time or not. When plotting the scaled Schoenfeld residuals in function of the time, no trend should be observed. A non-zero slope of the smoothing spline fit indicates violation of the assumption [6] [7] [8].

d) <u>Aalen's additive hazard model</u>

If the proportional hazard assumption doesn't hold and the Cox proportional hazard model can't be used, Aalen's additive hazard model may be a good alternative. The hazard function here is described as:

$$h(t|x) = \beta_0(t) + \beta_1(t).\, x_1 + \ldots + \beta_p(t).\, x_p = \beta_0(t) + \sum_{j=1}^{p} \beta_j(t).\, x_j$$

With:			
	t	=	time
	h(t)	=	hazard function
	$\beta_0(t)$	=	baseline hazard function
	x_j	=	covariate
	$\beta_j(t)$	=	regression coefficient ($j \neq 0$)
	p	=	total number of covariates in the model

One of the advantages of this model is that it allows for the regression coefficients $\beta_j(t)$ to change over time. As no assumption has been made about the functional forms of these regression functions $\beta_j(t)$, Aalen's additive model is a non-parametric model.

In order to interpret $\beta_1(t)$, the hazard rate has been described for two individuals who have the same values for all covariates except for covariate x_1 (a one unit difference):

Individual 1: $$h_1(t|x) = \beta_0(t) + \beta_1(t).\, x_1 + \ldots + \beta_p(t).\, x_p$$

Individual 2: $$h_2(t|x) = \beta_0(t) + \beta_1(t).\, [x_1 + 1] + \ldots + \beta_p(t).\, x_p$$
$$= \beta_0(t) + \beta_1(t).\, x_1 + \beta_1(t) + \ldots + \beta_p(t).\, x_p$$

Difference in hazard rate: $$\Delta h(t|x) = h_2(t|x) - h_1(t|x) = \beta_1(t)$$

The coefficient $\beta_1(t)$ can be interpreted as the change in hazard rate if there is a unit increase in covariate x_1 when all other covariates are kept constant.

Imagine now that only one covariate x_A is present in the model. x_A has a value 1 or 0 if the individual belongs to group A or B respectively. The hazard functions for both individuals are described as:

$$\textit{individual in group } A: \quad h(t|x_i) = \beta_0(t) + \beta_A(t)$$
$$\textit{individual in group } B: \quad h(t|x_i) = \beta_0(t)$$

The hazard ratio between these two is then:

$$HR_{B/A} = \frac{\beta_0(t) + \beta_A(t)}{\beta_0(t)}$$

Testing if $\beta_A(t) = 0$ is equivalent to testing if the hazard ratio is equal to one (the same test as was done with the Cox proportional hazard model) [9].

Aalen's additive hazard model can be fitted to the data using the function "*aalen()*" from the package "*timereg*" in R.

3.1.4 Dealing with competing risks

a) <u>Cause-specific hazard function</u>

In previous sections, it was always assumed that there was only one event that could occur. However, this is not always the case. When several different events are possible, but only one of these events can occur for a subject, then these events are called "competing events". A general approach for analysing competing risk data, is to estimate hazard rates and hazard ratios for the event of interest and to treat the competing events as censored. The cause-specific hazard function is here defined as:

$$h_c(t) = \lim_{\Delta t \to 0}\left(\frac{P\left[\, t \le T_c \le t + \Delta t \;\mid\; T \ge t \,\right]}{\Delta t}\right)$$

T_c is a random variable and denotes the time to event c. The cause-specific hazard function $h_c(t)$ gives the instantaneous rate of event c at time t, given no event occurred before time t. The Cox cause-specific proportional hazard model is described as:

$$h_c(t|\, x) = h_{0c}(t) \,.\, \exp\left(\sum_{j=1}^{p} \beta_{jc} \,.\, x_j\right)$$

It needs to be noticed here that the regression coefficient for the jth predictor is also subscribed by c, indicating that the effect of the predictors may be different for different events [3] [10].

b) Independence assumption

When, within any subgroup of interest, individuals who are censored at time t are representative of all individuals in that subgroup who remained at risk, then censoring is independent. A complication when studying competing risk data is that there are different types of censoring. First, there is the usual censoring in case the event didn't occur before the end of the study, if a subject withdraws from the study or if a subject is lost to follow up. In addition, when there are competing events, only one event at the time can be studied and the competing events are also considered as censoring. However the subjects who experienced a competing event are censored, no independent censoring assumption is needed as all the necessary information is available: it is known that the subject experienced a competing event and the time at which the event occurred is also known [3].

c) Cumulative incidence

A good approach for analysing competing risk data is to calculate cumulative incidences and to construct cumulative incidence curves. The incidence of an event at time t is an estimate for the marginal probability of that event occurring at time t when competing events are present. The incidence can be estimated as follows:

1. Estimate the hazard at time t for the event of interest $\hat{h}_c(t)$ (= the number of events of interest occurring at time t divided by the number of subjects at risk at time t).
2. A subject can only experience the event of interest at time t if the subject didn't experience any other event (event of interest or competing event) at the previous time t' = t-1 or wasn't censored at the previous time t'. The estimated probability of "surviving" the previous time t' is denoted by $\hat{S}(t')$. (Survival refers to overall survival instead of cause-specific survival.)
3. The estimated marginal probability or incidence of the event of interest occurring at time t is the product of $\hat{h}_c(t)$ and $\hat{S}(t')$.

If there are only competing events (no recurring events and no left-censoring), then the cumulative incidence of the event of interest at time t can be estimated by the cumulative percentage (= the cumulative number of events of interest until time t, divided by the total sample size) [3] [11].

3.1.5 Log rank test

In order to compare the hazard functions between two or more groups of subjects, the non-parametric log rank test can be executed. The null hypothesis and alternative hypothesis are:

$$H_0:\ h_1(t) = h_2(t) = ... = h_p(t)$$
$$H_a:\ at\ least\ two\ h(t)\ are\ different$$

First, consider the following situation at time t (see also Table 1): there are only two groups A and B, the number of subjects at risk in each group are N_A and N_B, the total number of subjects at risk is $N = N_A + N_B$, the number of subjects in each group experiencing the event of interest are O_A and O_B and the total number of subjects experiencing the event is $O = O_A + O_B$.

Table 1: Number of subjects at risk and number of subjects experiencing the event of interest for groups A and B.

	Number of subjects experiencing the event	Number of subjects at risk
Group A	O_A	N_A
Group B	O_B	N_B
Total	O	N

Under the null hypothesis, O_A follows a hypergeometric distribution:

$$P(O_A = x) = \frac{\binom{O}{x} \cdot \binom{N-O}{N_A - O_A}}{\binom{N}{O}}$$

and

$$E(O_A) = \frac{O}{N}.N_A$$

$$Var(O_A) = \frac{N_A . N_B . N . (N-O)}{N^2 .(N-1)}$$

The log rank test statistic is then defined as: $z_{\log rank} = \frac{\sum_{t=t_{start}}^{t_{end}} \left[O_A - E(O_A)\right]}{\sqrt{\sum_{t=t_{start}}^{t_{end}} Var(O_A)}}$

Under the null hypothesis $z_{\log rank} \sim N(0,1)$ or $z^2_{\log rank} \sim \chi^2_1$. When the observed test statistic is larger than the cut-off value, the null hypothesis can be rejected and the hazard functions for both groups are not the same.

When more than two groups are compared, the test is based on a multivariate version of the hypergeometric distribution. The test statistic $z^2_{\log rank}$ then follows a χ^2-distribution with the number of degrees of freedom equal to the number of considered groups minus one [12] [13].

3.1.6 Application in study

For this study, three events were of interest: receiving concordant PECs, TLD registration after receiving concordant PECs and reaching the combined endpoint after receiving concordant PECs. When analysing the time to event, censoring and possible competing risks were taken into account.

For the time from admission until receiving concordant PECs:

- Right censoring occurs when a patient didn't receive concordant PECs before the end of the study (after 28 days).
- There are two competing risks:
  1) The patient can be discharged from the ICU before he received concordant PECs.
  2) The patient may have died before he received concordant PECs.
- For 42 patients who did not receive concordant PECs, the discharge day or time of death was unknown. These patients were considered as censored at the first day of the study.

For the time from receiving concordant PECs until TLD registration:

- Right censoring occurs when a patient didn't have a TLD registration before the end of the study.
- There are two competing risks:
  1) The patient can be discharged from the ICU before a TLD was registered (the discharge day for all patients with concordant PECs was known).
  2) The patient may have died before a TLD was registered.

For the time from receiving concordant PECs until reaching the combined endpoint:

- Right censoring occurs when a patient didn't reach the combined endpoint one year after his ICU stay.
- There are 8 patients who are lost to follow up. It is unknown whether those patients have reached the combined endpoint or not. These patients are censored at time = discharge day – day of receiving concordant PECs.
- There are no competing events.

In order to compare the hazard rates of the events between different subgroups Cox (cause-specific) proportional hazard models were fitted to the data. Only one predictor was included in the models: the group indicator variable (= the variable responsible for the division of the patients in the age, cancer type, cancer status and surgery subgroups).

3.2 Inverse propensity score weighting

3.2.1 Experimental data versus observational data

A study can be based on experimental data from a randomised trial. The individuals participating in the study are randomly divided over two or more groups and a certain treatment is assigned to each group. Because of the randomization, there are no confounding issues. Randomized trials can however not always be carried out due to practical or ethical reasons. A lot of studies are therefore based on observational data, as was the case for this study. Information about patients, hospitals and clinicians was obtained during a certain time span. Based on patient characteristics, different subgroups were formed. The goal was to study the link between group membership and the outcome variable CEP. However, because no randomization process is involved, there may be confounding issues. For example, if there are more male patients in the first group than there are in the second group, then the association between group membership and CEP may be influenced by gender. In order to correct for systematic differences in background characteristics between the groups (confounders), inverse propensity score weighting was applied.

3.2.2 Propensity score

The basic idea of propensity score methods is to replace, for each patient in the study, all confounding background characteristics by one summarizing characteristic, called the propensity score. The propensity score for a patient is the probability that that patient belongs to a certain group given his set of covariates:

$$PS_A(x_i) = P(group = A \mid X = x_i)$$

The first step towards estimating the propensity scores is to find out which of the observed variables may be confounders. In this study, confounding variables are variables that are associated with the outcome variable CEP and with group membership. As CEP is a binary variable, logistic regression can be used to find variables that are associated with CEP. Next, multinomial logistic regression can be used to find out which of those variables are also associated with group membership. Finally, a multinomial logistic regression model, with the confounding variables as predictors, is used to estimate the propensity score [14] [15].

3.2.3 Inverse propensity score weighting

The weight for an individual is obtained by taking the inverse of the propensity score for the group to which the individual belongs:

$$i \in group\ A: \qquad W_i = \frac{1}{PS_A(x_i)} = \frac{1}{P(group = A \mid X = x_i)}$$

By applying inverse propensity score weighting a pseudo-population is created in which the covariates and the exposure variable are independent and therefore no confounding is present (a property that is also expected under randomization).

The reason for choosing the inverse of the propensity score as weight for each patients, it that the inverse probability weighted mean equals the counterfactual mean, assuming conditional exchangeability holds [16].

This equality can be described as follows:

$$E\left[\frac{I(group = g).Y}{P(group = g \mid X = x)}\right] = E\left[Y_g\right]$$

$I(group = g)$ has value 1 if a subject belongs to group g and has value 0 otherwise, Y is the binary outcome variable, $P(group = g \mid X = x)$ gives the probability for a subject to belong to group g given his set of covariates x (= the propensity score) and finally Y_g is the counterfactual outcome variable Y (outcome that would have been observed if the subject would have belonged to group g). Assuming conditional exchangeability means in the current study that, given the same set of covariates, patients in different subgroups are exchangeable (the same outcome is expected if group membership should be different). Because of the equality described above, it is possible after weighting to estimate the average group effect as if all subjects belonged to group A or B or etc. Specifically for this study it is for example possible to estimate the proportion of patients who should have received concordant PECs by the end of the study if every patient had active cancer (as if patients without cancer and patients with not active cancer would be replaced by patients with active cancer who have the same characteristics) [15] [16] [17] [18].

3.2.4 Disadvantages of inverse propensity score weighting

The use of propensity scores also has some limitations or disadvantages. Firstly, it can only be used to adjust for the observed confounding variables, this in contrast to randomization which tends to balance out the distribution of all observed and unobserved variables. Secondly, if the (multinomial) logistic regression model used to estimate the propensity scores is not completely correct, the results may be biased. Lastly, in some cases very large weights are obtained which can cause an imbalance of the covariates between different groups again. A possible ad hoc solution is trimming the extremely high weights at the 95th percentile of the weights [19].

4 Discrimination

4.1 Indications of discrimination

The original meaning of the word "discrimination" is "making a distinction". Over the course of the years however, the word got a more negative connotation in social and legal context. The meaning of the word changed into "making an unjustified distinction" (i.e. discrimination based on race or religion). In this study, the word "discrimination" is used in its original meaning.

Based on the available information for this study, it is impossible to conclude if clinicians do or do not discriminate between patients based on their age, cancer type, cancer status or surgery type. Additional information is needed (interviews with clinicians, the opinion of anthropologists, …). Based on the available dataset, it is only possible to look for some trends that would be expected if there was discrimination.

Imagine that a subgroup of patients was discriminated (concerning the level of care) by clinicians, then the following two trends should be observed:

<u>Trend A:</u> The proportion of patients who received concordant PECs or the rate of receiving those concordant PECs is significantly higher/lower in the discriminated subgroup than in the other subgroups of patients.

<u>Trend B:</u> After receiving concordant PECs, the patients in the discriminated subgroup are treated differently than the patients in the other subgroups. The proportion of patients with a TLD registration or the rate at which TLDs were registered is significantly higher/lower for the discriminated subgroup of patients than for the other subgroups of patients.

So, if there is discrimination concerning the level of care, trend A and trend B should be observed. However, it is not because trend A and trend B are observed that it can be concluded that there is discrimination. Observing both trends only means that discrimination may be possible.

4.2 How to detect indications of discrimination?

In order to check if trend A was observed, the following things were studied:

- Differences between subgroups in proportion of patients who received concordant PECs (see univariate analysis – section 5)
- Unweighted and weighted cumulative incidence curves for the time from admission until receiving concordant PECs (see sections 7 and 9).

In order to check if trend B was observed, the following things were studied:

- Differences in risk of TLD registration by the end of the study between subgroups (see section 6.5)
- Unweighted and weighted cumulative incidence curves for the time from receiving concordant PECs until TLD registration (see sections 7 and 9).

The cumulative percentage of patients receiving concordant PECs was calculated relative to the total number of patients in the subgroup. The cumulative percentage of patients with a TLD registration was calculated relative to the number of patients with concordant PECs in that subgroup.

As the point of interest lies in the possible difference in care after receiving concordant PECs, patients with a TLD registration before receiving concordant PECs were not considered for the cumulative incidence curves to check for the presence of trend B.

Hazard rates and cumulative incidences for different subgroups were compared by:

- visually analysing the cumulative incidence curves,
- estimating the cause-specific hazard ratios (based on the Cox proportional hazard model which only included one predictor: the group-indicator variable) and the associated 95 % confidence intervals,
- executing a log rank test.

Cause-specific hazard ratios are used to compare two groups at the time. Therefore, a reference group within each type of subgroups has been chosen:

- for age subgroups: < 75 year
- for cancer type subgroups: no cancer
- for cancer status subgroups: not active cancer
- for surgery subgroups: scheduled surgery

The remaining groups are always compared to the reference group (the group with the lowest incidence for receiving concordant PECs).

5 Univariate description: differences between subgroups

5.1 Studied subgroups

The 1641 patients in the study were divided into four types of subgroups (based on age, cancer type, cancer status or surgery type). An overview of the number of patients in each subgroup has been presented in Table 2. The first step was to do a univariate analysis, to check whether there are significant differences between the subgroups when only one variable at the time is considered. When this variable is categorical, a Pearson's X^2-tests or Fisher's exact tests was executed. When the variable is continuous, a Kruskal-Wallis test or Wilcoxon rank sum test was used.

Table 2: Overview of number of patients in each subgroup.

Type of subgroups	Number of patients in each subgroup		
Age	< 75 year old: 1236	≥ 75 year old: 405	
Cancer type	No cancer: 1254	Solid tumor: 299	Hematological cancer: 88
Cancer status	No cancer: 1254	Active cancer: 117	Not active cancer: 270
Surgery	No surgery: 1070	Scheduled surgery: 207	Unscheduled surgery: 364

5.2 Differences between age subgroups

The results of all statistical tests that compare the age subgroups (< 75 year old and ≥ 75 year old) have been presented in App Table 2 and App Table 3. In what follows, a selection of the differences will be discussed.

There are significantly more males present in the group with younger patients than in the group with older patients (60.6 % vs. 54.6 %). Older patients tend to have significantly more moderate to severe comorbidities (17.5 % vs. 9.2 % with two or more comorbidities). When looking at the type of comorbidities, it could be concluded that significantly more old patients have heart failure (20.5 % vs. 8.8%), COPD (14.6 % vs. 10.6%) and dementia (5.7 % vs. 1.3 %) and that significantly more young patients have hematological malignancy (6.5 % vs. 3.0 %) and liver cirrhosis (6.2 % vs. 1.2 %). Active smoking and drinking tend to occur more with younger than with older patients (21.4 % vs. 6.4% for smoking and 13.7 % vs. 3.0 % for drinking).

The results in App Table 10 showed that the proportion of patients who received concordant PECs is significantly higher for older patients than for younger patients (13.6 % vs. 8.5 %, p-value = 0.0038). The estimated difference in risk of receiving concordant PECs is 5.1 % with associated 95 % confidence interval [1.2 %, 8.9 %].

5.3 Differences between cancer type subgroups

The results of all statistical tests that compare the cancer type subgroups (no cancer, hematological cancer, solid tumor) have been presented in App Table 4 and App Table 5. In what follows, a selection of the differences will be discussed.

It appears that patients with hematological cancer are treated more often (39.8 %) in an ICU with a good ethical climate than patients without cancer (19.2 %) or patients with a solid tumor (15.1 %). As expected, there is also a significant difference in main admission reasons. Patients without cancer are for example more often admitted due to heart failure (19.5 % vs. 8.7 % and 10.2 %), neurological issues (12.5 % vs. 6.0 % and 8.0 %), multiple trauma (7.3 % vs. 1.3 % and 0.0 %) or head trauma (4.4 % vs. 1.0 % and 0.0 %). Patients with hematological cancer on the other hand are more often admitted because of sepsis (51.1 % vs. 17.7 % and 18.0 %). Another remarkable difference is that there are far fewer smokers among the patients with hematological cancer (1.1 %) than among the patients without cancer (19.2 %) or among the patients with a solid tumor (16.1 %). Alcohol problems seem to occur more often with patients without cancer (13.2 % vs. 4.3 % and 3.2 %). It could also be concluded that there is a significant difference between the three subgroups in ECOG performance status and in surgery category (no surgery, scheduled or unscheduled surgery).

The results in App Table 11 showed that 13.6 % of the patients with hematological cancer, 11.4 % of the patients with a solid tumor and 9.1 % of the patients without cancer received concordant PECs. Based on this data, it could not be concluded that there is a significant difference in the proportion of patients with concordant PECs between the cancer type subgroups (p-value = 0.2206). This was also confirmed by the results of the pairwise comparisons (see App Table 11). Every 95 % confidence interval for the estimated difference in risk of concordant PECs contained the value 0, indicating that no significant difference in risk was detected.

5.4 Differences between cancer status subgroups

The results of all statistical tests that compare the cancer status subgroups (no cancer, active cancer and not active cancer) have been presented in App Table 6 and App Table 7. In what follows, a selection of the differences will be discussed.

As expected, there is a significant difference in main admission reasons between the cancer status subgroups. There are for example more patients without cancer that are admitted due to heart failure (19.5 % vs. 9.4 % and 8.9 %), neurological issues (12.5 % vs. 6.8 % and 6.3 %), multiple trauma (7.3 % vs. 0.0 % and 1.5 %) or head trauma (4.4 % vs. 0.9 % and 0.7 %) and there are more patients with active or not active cancer that are admitted because of sepsis (29.1 % and 24.8 % vs. 17.7 %). There are also more patients without cancer that have alcohol (13.2 % vs. 0.9 % and 5.2 %) or smoking problems (19.2 % vs. 8.5 and 14.4 %).

A remarkable result is that significantly less patients with active cancer have a DNR-order before admission (74.4 % vs. 90.3 % for patients without cancer and 90.4 % for patients with not active cancer). On the other hand, it was also noticed that the proportion of patients who have a written withholding or withdrawing order within 24 hours after admission is larger in the group of patients with active cancer (9.4 % vs. 4.0 % for patients without cancer and 2.2 % for patients with not active cancer). There is also a significant difference between the three subgroups in ECOG performance and in type of surgery (no surgery, scheduled or unscheduled surgery).

The results in App Table 13 show that 9.1 % of the patients without cancer, 8.1 % of the patients with not active cancer and 20.5 % of the patients with active cancer have received concordant PECs and that these differences between the three groups were significant (p-value = 0.0002). The results of the pairwise comparisons have been presented in App Table 14. It could be concluded that there is a significant difference in the proportion of patients with concordant PECs between the group of patients without cancer and active cancer (p-value = 0.0002) and between the group of patients with active cancer and with not active cancer (p-value = 0.0010). The estimated risk of receiving concordant PECs is 11.4 % [3.5 %, 19.4 %] larger for patients with active cancer than for patients without cancer and 12.4 % [3.7 %, 21.0 %] larger for patients with active cancer than for patients with not active cancer. No evidence for a significant difference in risk of receiving concordant PECs between patients without cancer and with not active cancer was found (p-value = 0.7075).

5.5 Differences between surgery subgroups

The results of all statistical tests that compare the surgery subgroups (no surgery, scheduled surgery and unscheduled surgery) have been presented in App Table 8 and App Table 9. In what follows, a selection of the differences will be discussed.

For all groups, it appears that patients go to a university hospital rather than to a public hospital. This contrast is the largest for patients with a scheduled surgery: 77.3 % are treated in a university hospital and only 11.1 % are treated in a public hospital. This difference is less pronounced in the other subgroups (50.3 % vs. 25.5 % for patients without surgery and 59.1 % vs. 24.5 % for patients with unscheduled surgery). It also appears that more patients with a scheduled surgery are treated in large hospitals with more than 750 available beds (61.4 % vs. 40.9 % and 47.0 %) and that the ethical climate of the ICU is rather poor (60.9 % vs. 30.7 % and 46.2 %).

There were also some dissimilarities in patient characteristics. When looking at the type of comorbidities, it appeared that significantly more patients with scheduled surgery have a solid tumor (44.9 % vs. 13.6 % and 18.1 %) and that significantly more patients without surgery have a hematological malignancy (7.8 % vs. 1.9 % and 1.4 %). Another observed trend is that patients without surgery have less need of invasive mechanical ventilation or a vasopressor (50.0 % vs. 68.8 % and 73.4 %) during their ICU stay. There is also a significant difference between the three surgery groups in age distribution, in ECOG performance status and in reasons of admission at the ICU.

The results in App Table 15 show that 10.6 % of the patients without surgery, 4.8 % of the patients with scheduled surgery and 10.2 % of the patients with unscheduled surgery have received concordant PECs. These differences appeared to be significant (p-value = 0.0376). The results of the pairwise comparisons have been presented in App Table 16. The estimated risk of receiving concordant PECs is 5.8 % [2.0 %, 9.5 %] larger for patients without surgery than for patients with scheduled surgery and is 5.4 % [0.7 %, 10.0 %] larger for patients with unscheduled surgery than for patients with scheduled surgery. It could not be concluded that there was a significant difference in risk of receiving concordant PECs between the group of patients without surgery and with unscheduled surgery (p-value = 0.9092).

6 Risk of death within 28 days, death within one year, CEP and TLD

6.1 Goal and method

Within the subgroups, a distinction was made between patients who did and did not receive concordant PECs. Fisher's exact tests were applied to investigate whether there was a significant difference between the different subgroups in risk of death within 28 days, risk of death within 1 year, risk of reaching the combined endpoint and risk of TLD registration by the end of the study. It was investigated whether the same trend could be observed between patients with and without concordant PECs.

6.2 Risk of death within 28 days

An overview of the number of patients that have died within 28 days (during ICU stay or not) and the results of the Fisher's exact tests (to test for difference in risk of death within 28 days between the different subgroups) have been presented in App Table 17, App Table 18, App Table 19 and App Table 20 for the age, cancer type, cancer status and surgery subgroups respectively. There were 54 patients (53 patients without concordant PECs and 1 patient with concordant PECs) whose situation after 28 days was unknown. These patients were not taken into consideration for testing possible differences between the subgroups.

It could be concluded that for the patients without concordant PECs, there is a significant difference in risk of death within 28 days between the subgroups (p-values between 0.0005 and 0.0320). The risk is larger for older patients (22.6 %) than for younger patients (14.9 %), is larger for patients with hematological cancer (32.9 %) than for patients without cancer (15.4 %) and patients with a solid tumor (17.4 %), is larger for patients with active cancer (23.7 %) than for patients without cancer (15.4 %) and patients with not active cancer (19.8 %) and is also larger for patients without surgery (20.9 %) than for patients with scheduled surgery (4.1 %) or patients with unscheduled surgery (11.9 %).

When looking at the patients with concordant PECs, it could only be concluded, that there was a significant difference in risk of concordant PECs between the surgery subgroups (p-value = 0.0210). 64.6 % of the patients without surgery and 56.8 % of the patients with unscheduled surgery already died within 28 days, this in contrast with only 20.0 % of the

patients with scheduled surgery. For the age, cancer type and cancer status subgroups, no significant difference was detected (p-values between 0.4478 and 1).

All the percentages mentioned above are expressed relative to the total number of patients in the subgroup (including the patient whose situation after 28 days was unknown).

6.3 Risk of death within 1 year

An overview of the number of patients who have died within one year and the results of the Fisher's exact tests (to test for difference in risk of death within 1 year between the different subgroups) have been presented in App Table 21, App Table 22, App Table 23 and App Table 24 for the age, cancer type, cancer status and surgery subgroups respectively. There were 241 patients (235 patients without concordant PECs and 6 patients with concordant PECs) whose situation after 1 year was unknown. These patients were not taken into consideration for testing possible differences between the subgroups.

It could be concluded that for the patients without concordant PECs, there is a significant difference in risk of death within 1 year between the subgroups (all p-values = 0.0005). The risk is larger for older patients (36.0 %) than for younger patients (25.2 %), is larger for patients with hematological cancer (51.3 %) than for patients without cancer (23.9 %) and patients with a solid tumor (37.4 %), is larger for patients with active cancer (46.2 %) than for patients without cancer (23.9 %) and patients with not active cancer (38.0 %) and is also larger for patients without surgery (32.6 %) than for patients with scheduled surgery (14.7 %) or patients with unscheduled surgery (21.4 %).

When looking at the patients with concordant PECs, it could only be concluded that there was a significant difference in risk of death within 1 year between the surgery subgroups (p-value = 0.0310). Only 60.0 % of the patients with scheduled surgery died within 1 year, this in contrast with 83.2 % for patients without surgery and 75.7 % for patients with unscheduled surgery. For the age, cancer type and cancer status subgroups, no significant difference was detected (p-values between 0.5262 and 1).

All the percentages mentioned above are expressed relative to the total number of patients in the subgroup (including the patient whose situation after 1 year is unknown).

6.4 Risk of combined endpoint

An overview of the number of patients that have reached CEP and the results of the Fisher's exact tests (to test the difference in risk of reaching CEP between the different subgroups) have been presented in App Table 25, App Table 26, App Table 27 and App Table 28 for the age, cancer type, cancer status and surgery subgroups respectively. There were 339 patients (331 patients without concordant PECs and 8 patients with concordant PECs) for who it was unknown whether they have reached the combined endpoint or not. These patients were not taken into consideration for testing possible differences between the subgroups.

It could be concluded that for the patients without concordant PECs, there is a significant difference in risk of reaching the combined endpoint between the subgroups (p-values between 0.0005 and 0.0096). The risk is larger for older patients (51.4 %) than for younger patients (40.1 %), is larger for patients with hematological cancer (64.5 %) than for patients without cancer (39.7 %) and patients with a solid tumor (49.4 %), is larger for patients with active cancer (58.1 %) than for patients without cancer (39.7 %) and patients with not active cancer (50.8 %) and is also larger for patients without surgery (47.1 %) than for patients with scheduled surgery (33.0 %) or patients with unscheduled surgery (35.8 %).

When looking at the patients with concordant PECs, it could not be concluded that there was a significant difference in risk of reaching the combined endpoint between the subgroups (p-values between 0.1164 and 0.8856).

All the percentages mentioned above are expressed relative to the total number of patients in the subgroup (including the patient for who it was unknown if they reached the combined endpoint or not).

6.5 Risk of TLD

An overview of the number of patients who registered a TLD by the end of the study and the results of the Fisher's exact tests (to test for difference in risk or TLD registration between the different subgroups) have been presented in App Table 29, App Table 30, App Table 31 and App Table 32 for the age, cancer type, cancer status and surgery subgroups respectively.

For the patients without concordant PECs, a significant difference in risk of TLD registration by the end of the study has only been observed for the age subgroups and the surgery subgroups (p-value 3.8E-05 and 0.0005 respectively). The risk of TLD registration was larger for older patients (11.1 %) than for younger patients (4.7 %) and was also larger for patients without surgery (8.3 %) than for patients with scheduled surgery (1.0 %) and for patients with unscheduled surgery (3.4 %). No significant difference in risk of TLD registration was observed for the cancer type and cancer status subgroups (p-values 0.9790 and 0.2704 respectively).

For the patients with concordant PECs, no significant difference in risk of TLD registration by the end of the study was observed for any of the subgroups (p-values between 0.3538 and 0.8604).

6.6 Remark about patients without concordant PECs

For the patients without concordant PECs, whose level of care is appropriate according in the clinicians, it could be concluded that the mortality (risk of death within 28 days, risk of death within 1 year and risk of reaching CEP) was higher for older patients, for patients with hematological cancer, for patients with active cancer and for patients without surgery. This may be an indication that more patients in those subgroups should actually have received concordant PECs and their level of care may not have been appropriate.

7 Unweighted cumulative incidence curves

7.1 Age subgroups

The cumulative incidence curves for the time from admission until receiving the 2nd PEC for the age subgroups have been presented in Figure 2. A significant difference in the cause-specific hazard rate of receiving concordant PECs has been detected (p-value log rank test = 0.001). This rate is 1.75 [1.26, 2.43] times as large for older patients as for younger patients. At the end of the study, about 13.6 % of the older patients received concordant PECs, this in comparison to 8.5 % of the younger patients (see also App Table 10).

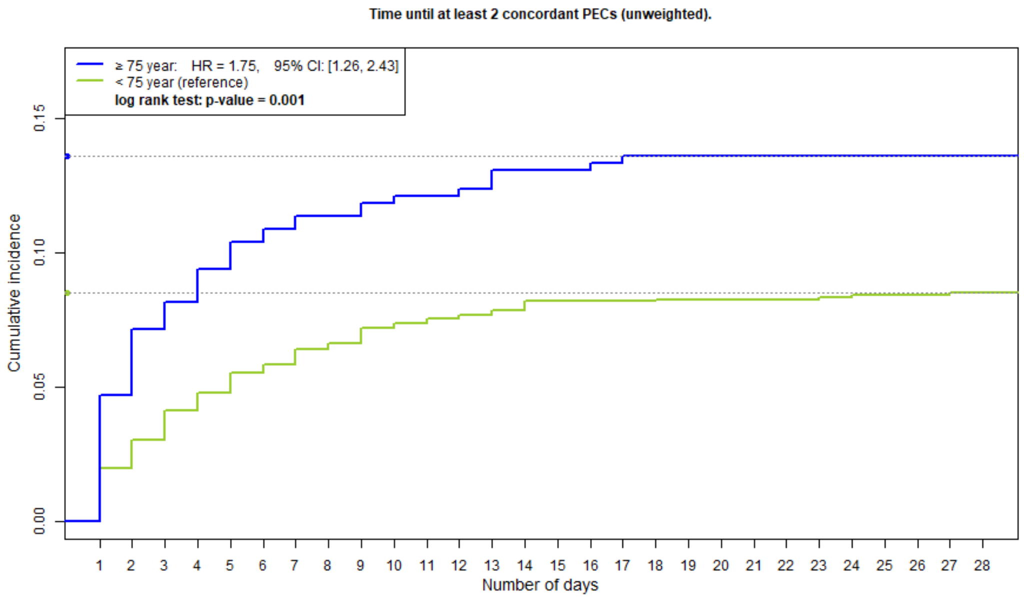

Figure 2: Comparison of age subgroups – cumulative incidence curves for time from admission until receiving of 2nd PEC.

The cumulative incidence curves for the time between receiving the 2nd PEC and TLD for the age subgroups have been presented in Figure 3. No significant difference in cause-specific hazard rate of TLD registration was observed (p-value log rank test = 0.924, HR = 1.03 [0.54, 1.96]). At the end of the study, 28.0 % of the younger patients and 27.5 % of the older patients with concordant PECs have registered a TLD (see App Table 29). It was also observed that most TLDs were registered within 14 days after receiving concordant PECs.

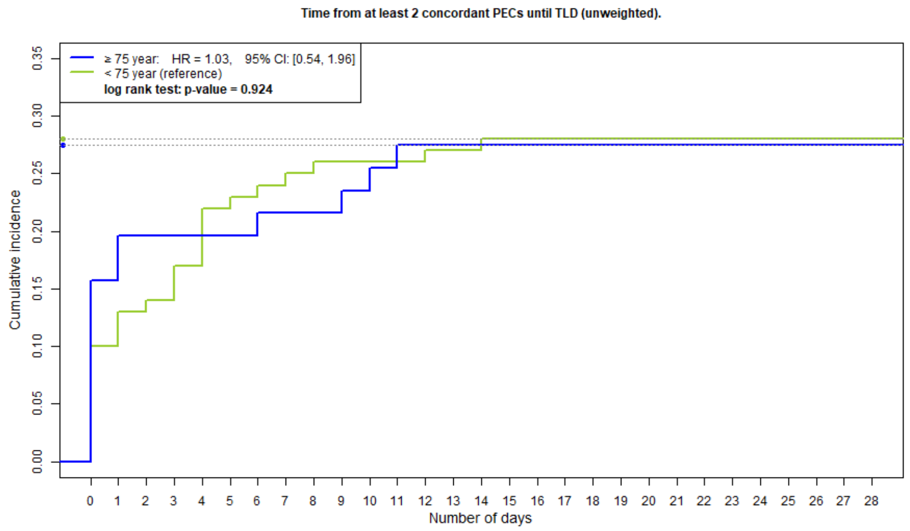

Figure 3: Comparison of age subgroups – cumulative incidence curves for time between receiving 2nd PEC and TLD.

7.2 Cancer type subgroups

The cumulative incidence curves for the time from admission until receiving the 2nd PEC for the cancer type subgroups have been presented in Figure 4. At the end of the study, 9.1 % of the patients without cancer, 13.6 % of the patients with hematological cancer and 11.4 % of the patients with a solid tumor have received concordant PECs (see also App Table 12). Because the proportional hazard assumption was not met (see App Figure 4), Aalen's additive hazard model was fitted instead of the cause-specific Cox proportional hazard model. The cause-specific hazard rate of receiving concordant PECs was significantly higher for patients with hematological cancer than for patients without cancer (p-value < 0.001). No significant difference was detected between patients with a solid tumor and patients without cancer (p-value = 0.665).

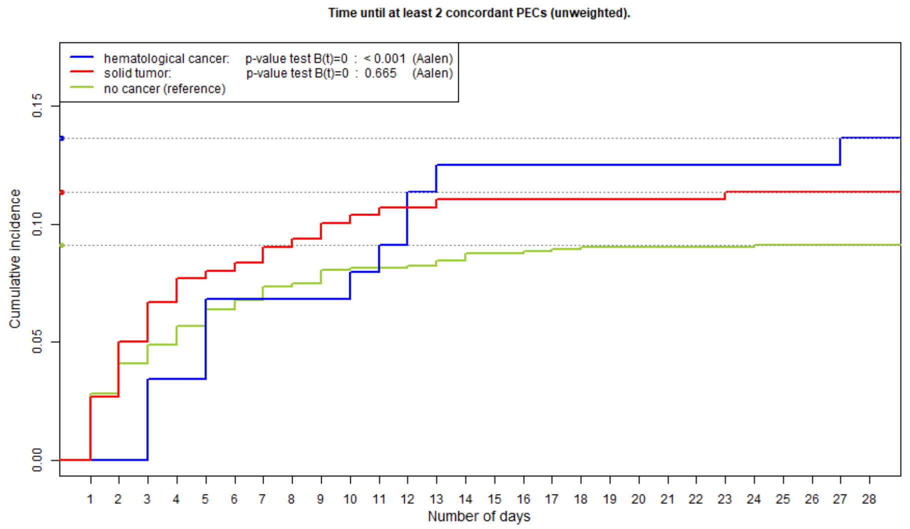

Figure 4: Comparison cancer type subgroups – cumulative incidence curves for time from admission until receiving of 2nd PEC.

The cumulative incidence curves for the time between receiving the 2nd PEC and TLD have been presented in Figure 5. By the end of the study, 29.2 % of the patients without cancer, 16.7 % of the patients with hematological cancer and 27.3 % of the patients with a solid tumor have registered a TLD (App Table 30). No significant difference in the cause-specific hazard rate in TLD registration between the three cancer type subgroups has been observed (p-value of log rank test = 0.688, HR_{hem} = 0.55 [0.13, 2.30] and HR_{solid} = 0.88 [0.42, 1.85]).

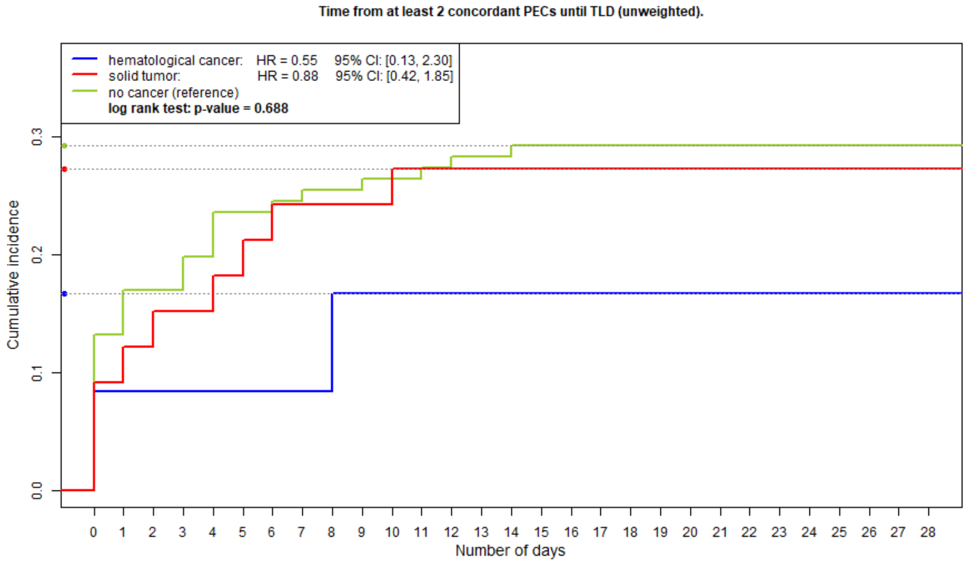

Figure 5: Comparison cancer type subgroups – cumulative incidence curves for time between receiving 2nd PEC and TLD.

7.3 Cancer status subgroups

The cumulative incidence curves for the time from admission until receiving the 2nd PEC for the cancer status subgroups have been presented in Figure 6. At the end of the study, 9.1 % of the patients without cancer and 8.1 % of the patients with not active cancer have received concordant PECs, this in contrast with 20.5 % of the patients with active cancer (see also App Table 14). A significant difference has been observed between the three subgroups in the cause-specific hazard rate of receiving concordant PECs (p-value log rank test = 7.7E-05). This rate was 2.84 [1.59, 5.07] times larger for patients with active cancer than for patients with not active cancer. No significant difference has been observed between the patients without cancer and patients with not active cancer (cause-specific hazard ratio was 1.16 [0.73, 1.83]).

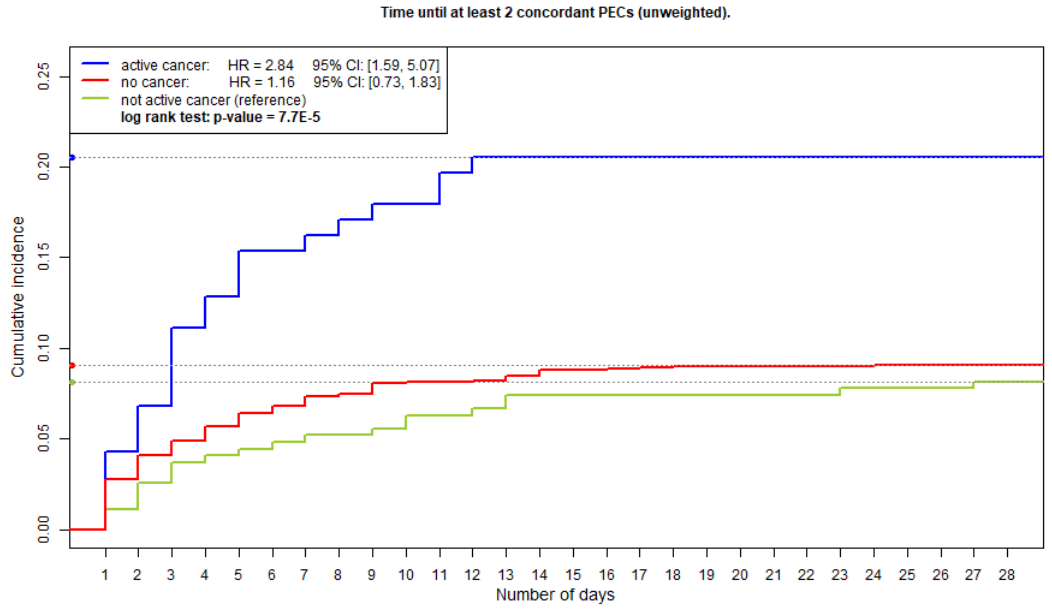

Figure 6: Comparison cancer status subgroups – cumulative incidence curves for time from admission until receiving of 2nd PEC.

The cumulative incidence curves for the time between receiving the 2nd PEC and TLD for the cancer status subgroups have been presented in Figure 7. No significant differences in cause-specific hazard rate between the three subgroups has been observed (p-value log rank test = 0.769, HR_{active} = 1.22 [0.37, 4.01] and $HR_{no\ cancer}$ = 1.40 [0.54, 3.60]). At the end of the study, 25.0 % of the patients with active cancer, 23.8 % of the patients with not active cancer and 29.2 % of the patients without cancer have registered a TLD (see App Table 31).

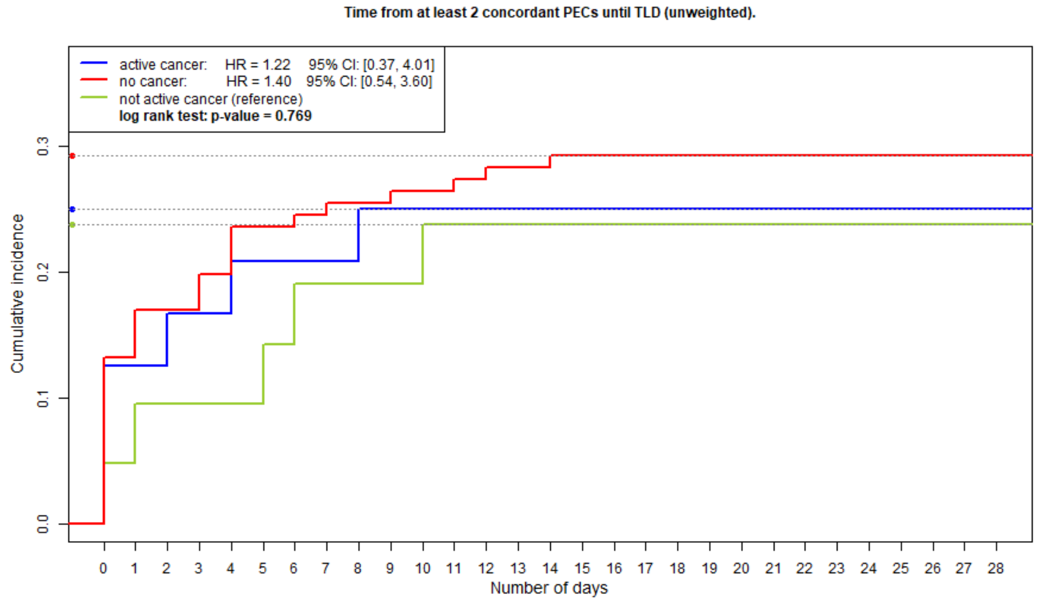

Figure 7: Comparison cancer status subgroups – cumulative incidence curves for time between receiving 2nd PEC and TLD.

7.4 Surgery subgroups

The cumulative incidence curves for the time from admission until receiving the 2nd PEC for the surgery subgroups have been presented in Figure 8. At the end of the study, 10.6 % of the patients without surgery and 10.2 % of the patients with unscheduled surgery received concordant PECs, this in contrast with only 4.8 % of the patients with scheduled surgery (see also App Table 16). No significant difference in the cause-specific hazard rate of receiving concordant PECs between the three subgroups has been detected (p-value log rank test = 0.193, $HR_{no\ surgery}$ = 1.79 [0.94, 3.43] and $HR_{unscheduled}$ = 1.80 [0.89, 3.61]).

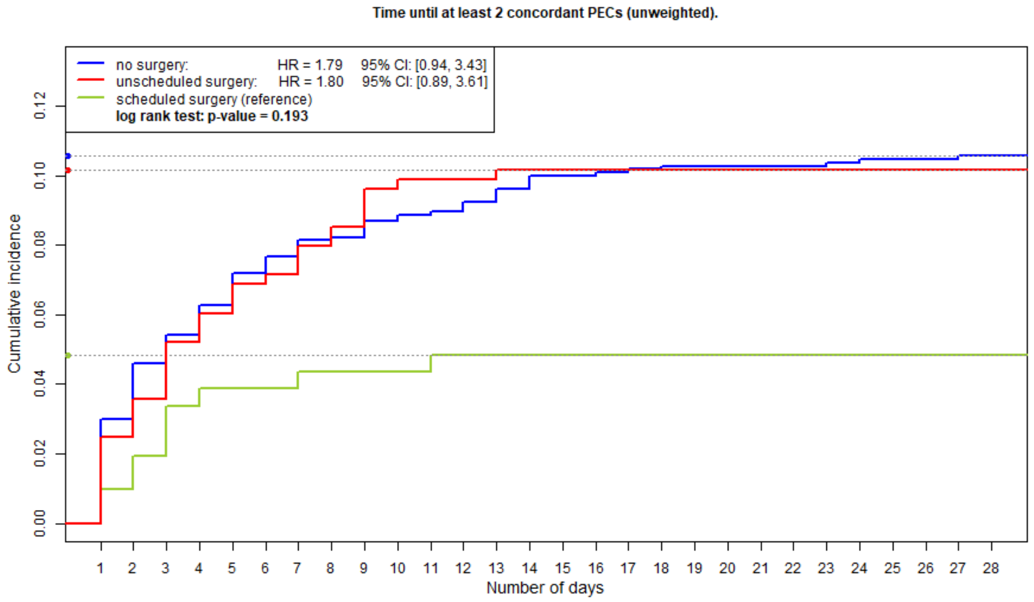

Figure 8: Comparison of surgery subgroups – cumulative incidence curves for time from admission until receiving of 2nd PEC.

The cumulative incidence curves for the time between receiving the 2nd PEC and TLD have been presented in Figure 9. First, it needs to be noticed that there is only one patient with a TLD registration in the reference group (patients with scheduled surgery). This is why the 95 % confidence intervals of the cause-specific hazard ratios are very wide. Due to this issue, the focus lies on the visual interpretation of the cumulative incidence curves. The steepness of the cumulative incidence curves for patients without surgery and patients with unscheduled surgery seems similar which may be an indication that the rate of TLD registration may be similar as well. At the end of the study, only 10.0 % of the patients with scheduled surgery had a TLD registration, this in contrast with 27.9 % for patients without surgery and 32.4 % for patients with unscheduled surgery (see App Table 32).

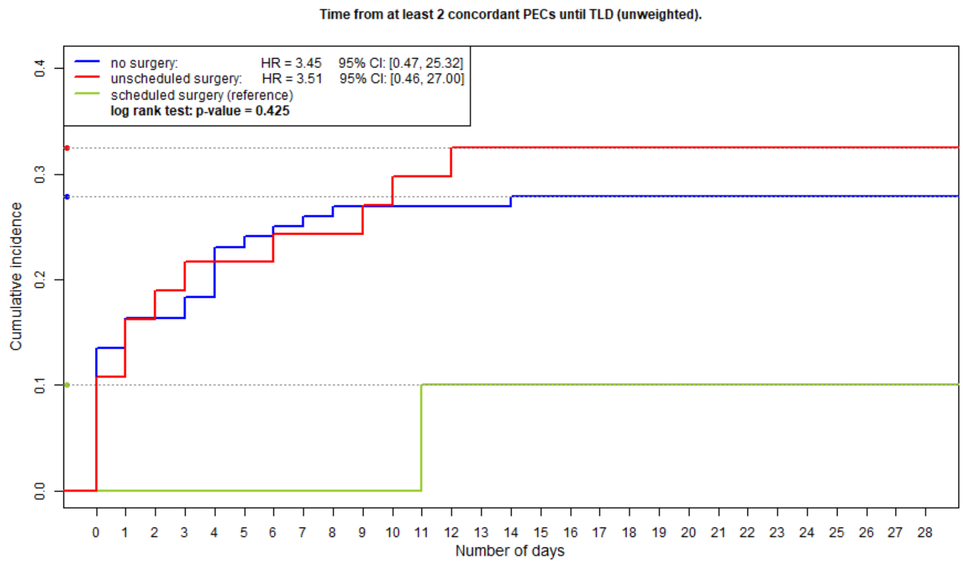

Figure 9: Comparison of surgery subgroups – cumulative incidence curves for time between receiving 2nd PEC and TLD.

7.5 First indication of discrimination?

An overview of the observed differences between the subgroups in the proportion of patients with concordant PECs, the proportion of patients with a TLD registration, the cause-specific hazard rate of receiving concordant PECs and the cause-specific hazard rate of TLD registrations (which have been discussed in previous sections) is shown in Table 3.

Although there is a significant difference between some subgroups in the proportion of patients with concordant PECS or in the cause-specific hazard rate of receiving those concordant PECs, there was never a significant difference observed in the proportion of patients with a TLD registration or in the cause-specific hazard rate of those TLD registrations. Therefore, it can be concluded that the observations do not point towards discrimination (trend A was observed, but not trend B – see section 4.1).

Table 3: Overview of differences between subgroups (↑ and ↓ mean "significantly more" and "significantly less" respectively).

	Proportion of patients with concordant PECs (1*)	Cause-specific hazard rate of receiving concordant PECs (2*)	Proportion of patients with a TLD registrations (3*)	Cause-specific hazard rate of TLD registrations (4*)
**Age subgroups**	**≥75 year old ↑**<br>p = 0.0038	**≥75 year old ↑**<br>$HR_{old/young}$ = 1.75 [1.26, 2.43]	**no significant difference**<br>p = 0.8606	**no significant difference**<br>$HR_{old/young}$ = 1.03 [0.54, 1.96]
**Cancer type subgroups**	**no significant difference**<br>no cancer - solid tumor: p = 0.2726<br>no cancer - hematological cancer: p = 0.2209<br>solid tumor - hematological cancer: p = 0.6967	**hematological cancer ↑ (relative to patients without cancer)**<br>hematological cancer - no cancer: $p < 0.001$<br>solid tumor - no cancer: p = 0.665	**no significant difference**<br>p = 0.4908	**no significant difference**<br>$HR_{hem/no\ cancer}$ = 0.55 [0.13, 2.30]<br>$HR_{sol/no\ cance}$r = 0.88 [0.42, 1.85]
**Cancer status subgroups**	**active ↑ (relative to not active and no cancer)**<br>no cancer - active: p = 0.0002<br>active - not active: p = 0.0010<br>no cancer - not active: p = 0.7075	**active ↑ (relative to not active)**<br>$HR_{active/not\ active}$ = 2.84 [1.59, 5.07]	**no significant difference**<br>p = 0.6872	**no significant difference**<br>$HR_{active/not\ active}$ = 1.22 [0.37, 4.01]<br>$HR_{no\ cancer/not\ active}$ = 1.40 [0.54, 3.60]
**Surgery subgroups**	**scheduled surgery ↓ (relative to unscheduled surgery and no surgery)**<br>no surgery - scheduled: p = 0.0151<br>scheduled - unscheduled: p = 0.0384<br>no surgery - unscheduled: p = 0.9092	**no significant difference**<br>$HR_{no\ surg/sched}$ = 1.79 [0.94, 3.43]<br>$HR_{unsched/sched}$ =1.80 [0.89, 3.61]	**no significant difference**<br>p = 0.3538	**no significant difference**<br>$HR_{no\ surg/sched}$ = 3.45 [0.47, 25.32]<br>$HR_{unsched/sched}$ =3.51 [0.46, 27.00]

(1*) p-values pairwise comparisons - Fisher's exact test (see univariate description - appendix A.2.2)
(2*) Hazard ratios (see cumulative incidence curves for time from admission until 2nd PEC - section 7.1 to 7.4)
(3*) p-values Fisher's exact test (see risk of TLD registration by end of the study - appendix A.3.4)
(4*) Hazard ratios (see cumulative incidence curves for time from 2nd PEC until TLD registration - section 7.1 to 7.4)

7.6 Difference in mortality rate between subgroups

7.6.1 Goal and method

Next, cumulative incidence curves for the time between receiving the 2nd PEC and death or CEP for the different subgroups were obtained. The time-between-events can vary from 0 to 365 days. However, it has been observed that most patients who died, died within the first 30 days. In order to obtain cumulative incidence curves, where the change in cumulative incidence is clearly shown especially within those first 30 days, it was decided to partially categorise the time between receiving the 2nd PEC and death or CEP. Patients with a time-between-events of 31 – 50 days, 51 – 100 days, 101 – 200 days, 201 – 300 days and 301 – 365 days were considered together. No categorization was applied if the time between the two events was between 0 and 30 days. The cumulative percentage was calculated relative to the number of patients with concordant PECs in that subgroup.

As there were no competing events for dying or for reaching the combined endpoint, an ordinary Cox proportional hazard model was used (instead of a cause-specific model) to compare hazard rates between the different subgroups. As there is no information available about the cause of death of the patients (whether they died because there was a unofficial treatment limitation decision or whether they died due to their underlying disease trajectory), possible differences in mortality rate between different subgroups cannot be considered as an indication of discrimination.

7.6.2 Age subgroups

The cumulative incidence curves for the time between receiving the 2nd PEC and death or CEP for the age subgroups have been presented in Figure 10. After one year, 88.6 % of the younger patients and 85.5 % of the older patients with concordant PECs have reached the combined endpoint (see App Table 25). No significant difference in mortality rate between the two subgroups has been observed (p-value log rank test = 0.924, HR = 1.02 [0.72, 1.45]).

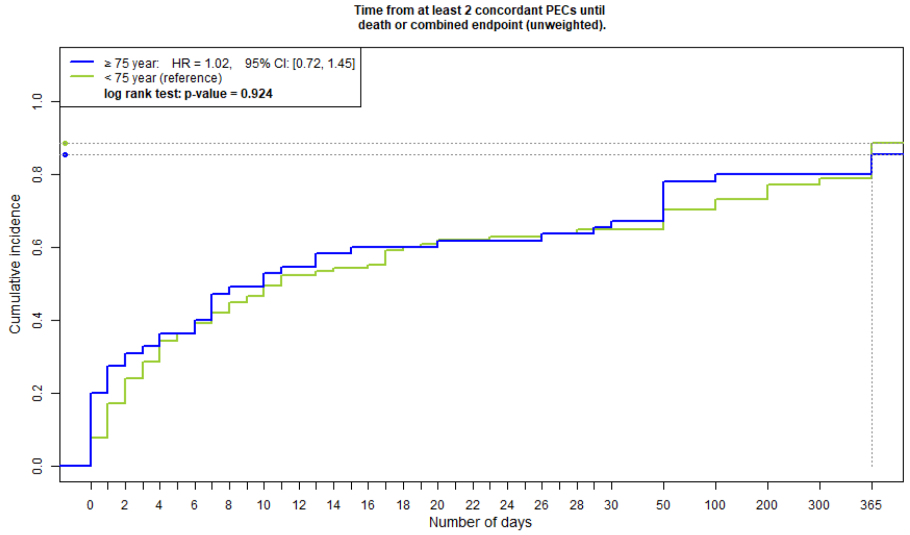

Figure 10: Comparison of age subgroups – cumulative incidence curves for time between receiving 2nd PEC and death or CEP.

7.6.3 Cancer type subgroups

The cumulative incidence curves for the time between receiving the 2nd PEC and death or CEP for the cancer type subgroups have been presented in Figure 11. After one year, 86.8 % of the patients without cancer, 91.7 % of the patients with hematological cancer and 88.2 % of the patients with a solid tumor have reached CEP (see App Table 26). No significant difference in mortality rate between the three subgroups has been observed (p-value log rank test = 0.356, HR_{hem} = 1.45 [0.78, 2.73] and HR_{solid} = 0.88 [0.58, 1.32]). It is however remarkable that 83.3 % of the patients with hematological cancer had already died within 10 days after receiving the 2nd PEC.

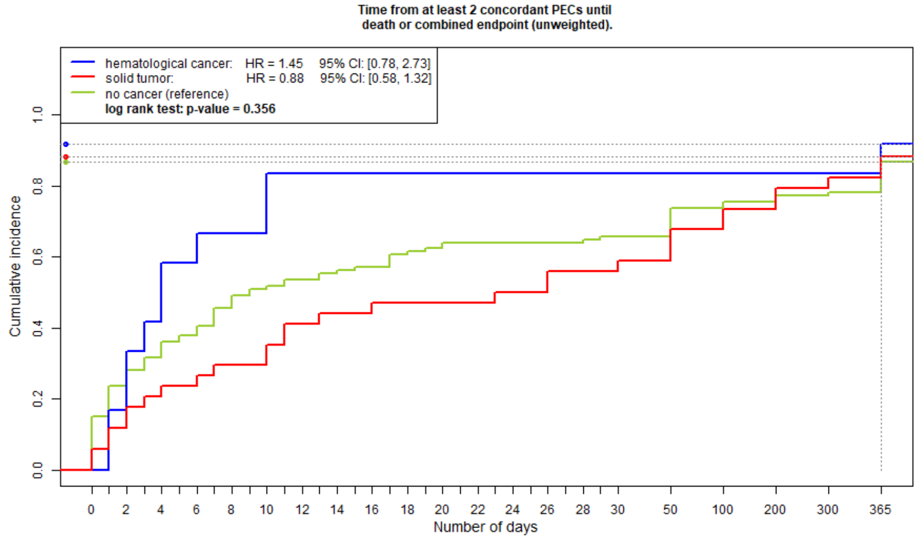

Figure 11: Comparison cancer type subgroups – cumulative incidence curves for time between receiving 2nd PEC death or CEP.

7.6.4 Cancer status subgroups

The cumulative incidence curves for the time between receiving the 2nd PEC and death or CEP for the cancer status subgroups have been presented in Figure 12. After one year, 95.8 % of the patients with active cancer, 81.8 % of the patients with not active cancer and 86.8 % of the patients without cancer have reached the combined endpoint (see App Table 27). Again, no significant difference in mortality rate between the three subgroups has been observed (p-value log rank test = 0.852, HR_{active} = 1.19 [0.64, 2.21] and $HR_{no\ cancer}$ =1.12 [0.68, 1.86]).

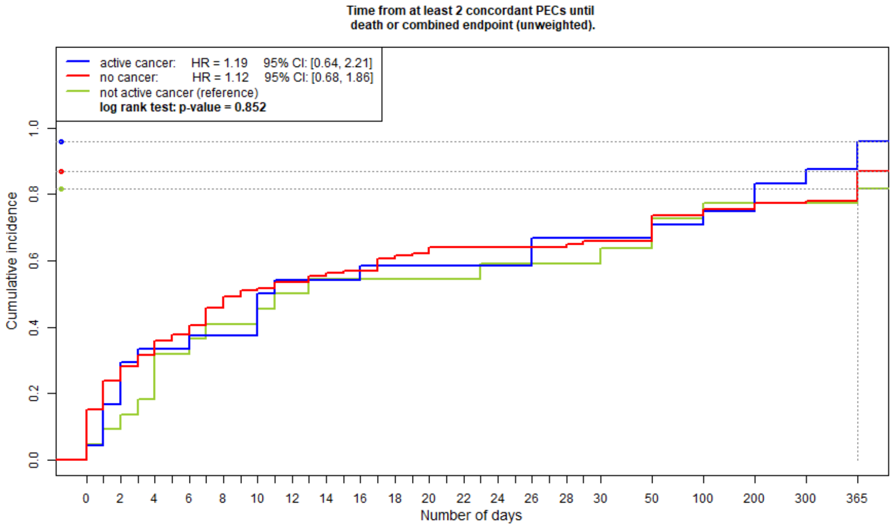

Figure 12: Comparison cancer status subgroups – cumulative incidence curves for time between receiving 2nd PEC death/CEP.

7.6.5 Surgery subgroups

The cumulative incidence curves for the time between receiving the 2nd PEC and death or CEP for the surgery subgroups have been presented in Figure 13. After one year, 89.4 % of the patients without surgery, 86.5 % of the patients with unscheduled surgery and 70.0 % of the patients with scheduled surgery have reached the combined endpoint (see App Table 28). A significant difference in mortality rate between the three subgroups has been detected (p-value log rank test = 0.044). The mortality rate of patients without surgery and patients with scheduled surgery is respectively 2.44 [1.13, 5.26] and 1.92 [0.85, 4.36] times as large as the mortality rate of patients with a scheduled surgery. This may be an indication that doctors are more reluctant to give up on patients who had a scheduled surgery and that they try to keep those patients alive as long as possible.

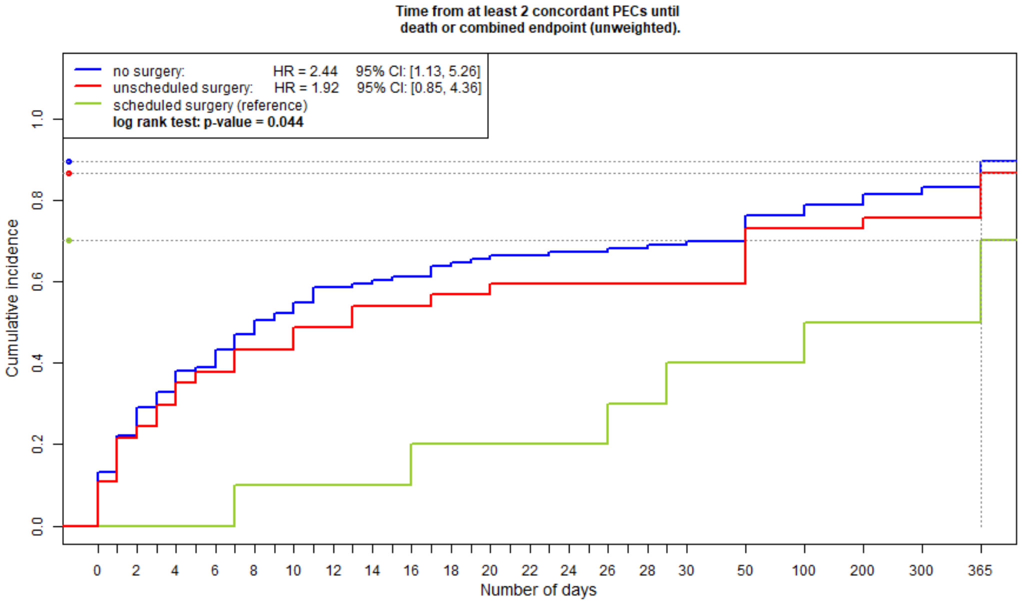

Figure 13: Comparison of surgery subgroups – cumulative incidence curves for time between receiving 2nd PEC death or CEP.

8 Inverse propensity score weighting

8.1 Goal and method

As was explained in section 3.2, inverse propensity score weighting was applied in order to correct for systematic differences in background characteristics between the groups (confounding). The following steps need to be followed in order to obtain the weights for each patient for the age subgroups:

1. Find the variables that are related to the outcome variable CEP.
2. Find out which of those variables are also related with age.
3. Estimate the propensity score.
4. Calculate the weights.

These steps will be further explained in following sections.

8.2 Variables related with CEP

The variable CEP was converted to a binary variable with value 1 if the patient was dead, not at home or had a poor QOL and value 0 if the patient was still alive, at home and had a good QOL after one year. The 339 patients for who the situation after one year was unknown, were not taken into consideration.

The first step towards obtaining the weight for each patient, was to look for the variables that are related with the binary variable CEP. This is done by executing a forward, backward and both-way stepwise logistic regression (significance level set to 0.10) with CEP as the outcome variable and patient, ICU and hospital characteristics as predictors. Only the variables that contain information about the patient known at the moment of admission at the ICU have been considered. Five of these variables contained 125 missing values and were therefore not included. All stepwise procedures lead to the same model, which contains 16 variables. An overview of these variables, the coefficient estimates and the p-values indicating the significance is presented in Table 4.

Table 4: Overview of variables related with CEP, the coefficient estimates and p-values.

Patient characteristics			
**Variable**	**Estimate β**	**SE**	**p-value**
- alcohol problems	0.54	0.23	0.0165
- age (< 75 year old or ≥ 75 year old)	0.36	0.16	0.0205
- head trauma as admission reason	1.32	0.42	0.0009
- liver failure as admission reason	0.43	0.25	0.0873
- neurologic reason for admission	0.85	0.25	0.0004
- number of comorbidities	0.40	0.12	0.0010
- liver cirrhosis as comorbidity	-0.77	0.35	0.0260
- whether the patient is competent			1.4E-09
competent	-0.71	0.32	
not competent	0.20	0.32	
- whether the patient has a DNR before admission			0.0067
no CPR	1.17	0.55	
withholding	1.45	0.64	
unknown	0.12	0.41	
- surgery (no surgery, scheduled surgery or unscheduled surgery			1.1E-05
scheduled	-0.78	0.21	
unscheduled	-0.63	0.17	
- cancer status (no cancer active cancer or not active cancer)			0.0263
no cancer	-0.84	0.33	
not active	-0.59	0.34	
- ECOG performance status			9.6E-13
full functional	-1.31	0.36	
functional but not able to work	-0.40	0.37	
limited functionality	0.06	0.39	
symptomatic	-0.91	0.36	
unknown	0.72	0.54	

ICU and hospital characteristics			
**Variable**	**Estimate β**	**SE**	**p-value**
- total number of beds in the ICU	-0.02	0.01	0.0001
- patient-nurse ratio	0.20	0.09	0.0210
- patient-junior physician ratio	0.08	0.03	0.0207
- total number of beds in the hospital			0.0009
>750	0.21	0.38	
250-500	0.18	0.39	
500-750	-0.52	0.37	

8.3 Variables related with age

The variable age is converted to a binary variable with the value 1 for patients younger than 75 years old and the variable 0 for patients that are 75 years or older. The next step towards obtaining the weight for each patient was to find out which of the variables related with CEP (see Table 4) and all possible two-way interactions between those variables, are also related with the binary variable age. This is done by applying a both-ways stepwise multinomial logistic regression (significance level set to 0.20). This resulted in a model that contains 17 variables and two-way interactions. Although the gender of the patient, the geographical region of the hospital and the ethical climate of the ICU didn't seem to be significantly related with CEP, it does seem important to balance out their effect as well. Therefore, they are also included. An overview of the selected variables and interactions, their coefficient estimates and p-values of significance are presented in Table 5.

Table 5: Overview of variables related with CEP and age, the coefficient estimates and p-values.

Main effects			
**Patient characteristics**			
**Variable**	**Estimate β**	**SE**	**p-value**
- alcohol problems	1.80	0.33	6.9E-11
- head trauma as admission reason	-2.07	0.75	0.0259
- number of comorbidities	-1.25	0.52	0.0174
- liver cirrhosis as comorbidity	13.00	0.38	2.5E-06
- whether the patient has a DNR before admission			0.0120
no CPR	-0.26	0.32	
withholding	-1.05	0.35	
unknown	0.42	0.35	
- surgery			0.0264
scheduled	-0.16	0.28	
unscheduled	0.07	0.21	
- cancer status			0.0013
no cancer	-2.03	0.62	
not active	-1.16	0.71	
- ECOG performance status			9.6E-05
full functional	0.55	0.51	
functional but not able to work	-0.75	0.54	
limited functionality	-0.76	0.54	
symptomatic	-0.30	0.52	
unknown	-0.30	0.56	
**ICU and hospital characteristics**			
**Variable**	**Estimate β**	**SE**	**p-value**
- total number of beds in the hospital			0.0446
>750	0.27	0.27	
250-500	-0.22	0.30	
500-750	0.08	0.30	
**Manually added variables**			
**Variable**	**Estimate β**	**SE**	**p-value**
- gender	0.16	0.13	0.2107
- geographical region			0.7640
Northern Europe	-0.25	0.26	
Southern Europe	-0.25	0.32	
Western Europe/USA	-0.20	0.23	
- ethical climate			0.0970
good	0.09	0.22	
poor	-0.33	0.17	

Interaction effects					
**Variable**			**Estimate β**	**SE**	**p-value**
- comorbidities	*	ECOG performance status			0.0012
comorbidities	*	full functional	-0.24	0.04	
comorbidities	*	funtional but not able to work	0.61	0.41	
comorbidities	*	limited functionality	0.73	0.41	
comorbidities	*	symptomatic	0.60	0.40	
comorbidities	*	unknown	0.90	0.51	
- comorbidities	*	surgery			0.0064
comorbidities	*	scheduled	-0.48	0.25	
comorbidities	*	inscheduled	-0.60	0.21	
- surgery	*	liver cirrhosis			0.0012
scheduled	*	liver cirrhosis	7.04	/ (*1)	
unscheduled	*	liver cirrhosis	35.80	/ (*1)	
- liver cirrhosis	*	ECOG performance status			0.0048
liver cirrhosis	*	full functional	3.77	1.7E-06	
liver cirrhosis	*	funtional but not able to work	-6.68	0.50	
liver cirrhosis	*	limited functionality	-23.65	0.41	
liver cirrhosis	*	symptomatic	6.63	5.4E-07	
liver cirrhosis	*	unknown	4.73	8.4E-07	
- head trauma	*	total beds in hospital			0.0231
head trauma	*	>750	1.91	0.99	
head trauma	*	250-500	24.29	/ (*2)	
head trauma	*	500-750	0.14	0.94	
- liver cirrhosis	*	total beds in hospital			0.0983
liver cirrhosis	*	>750	9.86	0.73	
liver cirrhosis	*	250-500	-6.83	0.50	
liver cirrhosis	*	500-750	9.97	0.81	
- cancer status	*	comorbidities			0.0378
no cancer	*	comorbidities	0.90	0.41	
not active	*	comorbidities	0.49	0.47	
- surgery	*	head trauma			0.2971
scheduled	*	head trauma	0.00	0.00	
unscheduled	*	head trauma	1.27	0.83	

(*1): no older patients with liver cirrhosis and a scheduled or unscheduled surgery

(*2): no older patients were admitted in a hospital with 250-500 beds due to a head trauma

8.4 Propensity scores and weights for age

The next step was to use the multinomial logistic regression model to predict the probability that a patient is younger than 75 years old, given his set of covariates. All variables presented in Table 5 were used as predictors in this model. The weight for a patient is the inverse of the probability to belong to their own age group (inverse of the propensity score):

Weight for a patient younger than 75 years: $$w_i = \frac{1}{P\left(< 75\,year \mid x_i\right)}$$

Weight for a patient of 75 years or older: $$w_j = \frac{1}{P\left(\geq 75\,year \mid x_j\right)} = \frac{1}{1 - P\left(< 75\,year \mid x_j\right)}$$

When using inverse propensity score weighting, there is always a risk that some observations have extremely high weights which can greatly influence the results. According to literature [19], a possible ad hoc solution lies in replacing all weights that are larger than the 95th percentile by the weight of the 95th percentile. This process is called trimming of the weights.

8.5 Propensity scores and weights for cancer type, cancer status and surgery

In the previous section it was explained how the propensity scores and weights for the age subgroups were obtained. A similar method wa used to calculate the weights when the cancer type, cancer status and surgery subgroups are considered. The main difference is that the patients are now divided into three groups instead of two. The three groups are referred to as group A, group B and group C to keep the explanation general.

Multinomial logistic regression models were built in a similar way as explained in section 8.4. The final models were used to estimate three propensity scores for each patient (estimated probability to belong to group A, group B and group C, given his set of covariates). The single weight for each patient could then be calculated as follows:

Weight for a patient in group A: $$w_i = \frac{1}{PS_A(x_i)} = \frac{1}{P(group\ A \mid x_i)}$$

Weight for a patient in group B: $$w_j = \frac{1}{PS_B(x_j)} = \frac{1}{P(group\ B \mid x_j)}$$

Weight for a patient in group C: $$w_k = \frac{1}{PS_C(x_k)} = \frac{1}{P(group\ C \mid x_k)}$$

Trimming of the weights at the 95th percentile was again applied to avoid extremely large weights that may have a big influence on the results.

9 Weighted cumulative incidence curves

9.1 Calculation of cumulative incidence

In section 8 it was explained how the weights for age, cancer status, cancer type and surgery were obtained for each patient. An overview of the ranges of the weights has been presented in App Table 34. The next step is to use these weights to construct the weighted cumulative incidence curves. The incidence at time t is now calculated by dividing the sum of the weights of the patients experiencing the event at time t by the sum of the weights of all patients in the group. Checking for indications of discrimination is done in a similar way as with the unweighted cumulative incidence curves (see section 4.2).

9.2 Age subgroups

The weighted cumulative incidence curves for the time from admission until receiving the 2nd PEC for the age subgroups have been presented in Figure 14. If every young patient would be replaced by an older patient with the same characteristics (same values for the predictors mentioned in Table 5), then 13.4 % of the patients would have received concordant PECs by the end of the study. Similarly, if every older patient would be replaced by a younger patients with the same characteristics, then 8.9 % of the patients would have received concordant PECs. The cause-specific hazard rate of receiving concordant PECs is 1.65 [1.17, 2.32] times larger for older patients than for younger patients (p-value robust log rank test = 0.010).

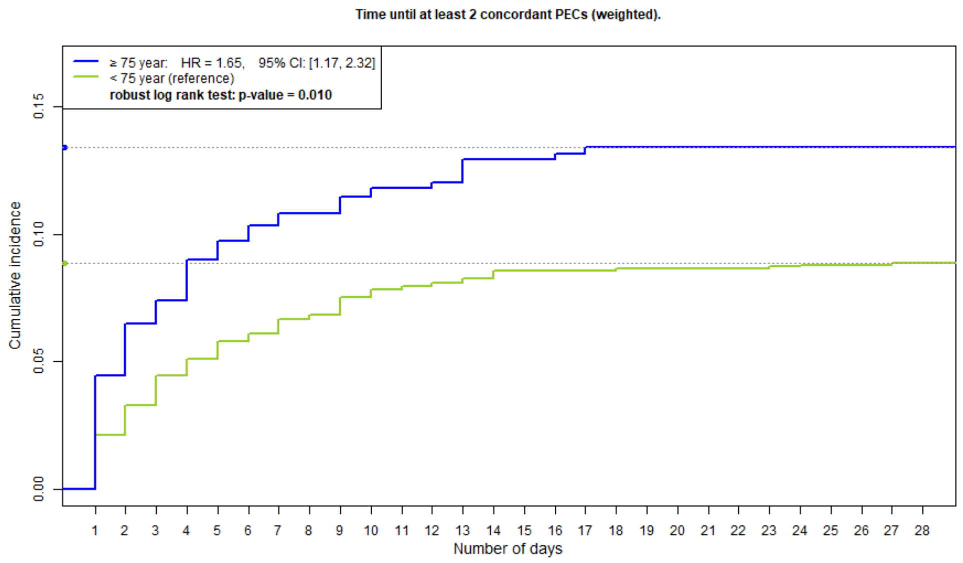

Figure 14: Comparison age subgroups – weighted cumulative incidence curves for time from admission until receiving 2nd PEC.

The weighted cumulative incidence curves for the time between receiving the 2nd PEC and TLD have been presented in Figure 15. If every young patient would be replaced by an older patient with the same characteristics, then 28.0 % of the patients would have had a TLD registration by the end of the study. Similarly, if every older patient would be replaced by a younger patients with the same characteristics, then 29.2 % of the patients would have received a TLD registration. No significant difference in cause-specific hazard rate of TLD registration between the two age groups has been observed (p-value robust log rank test = 0.958, HR = 0.98 [0.51, 1.91]).

These results coincide with the results that were found based on the unweighted cumulative incidence curves.

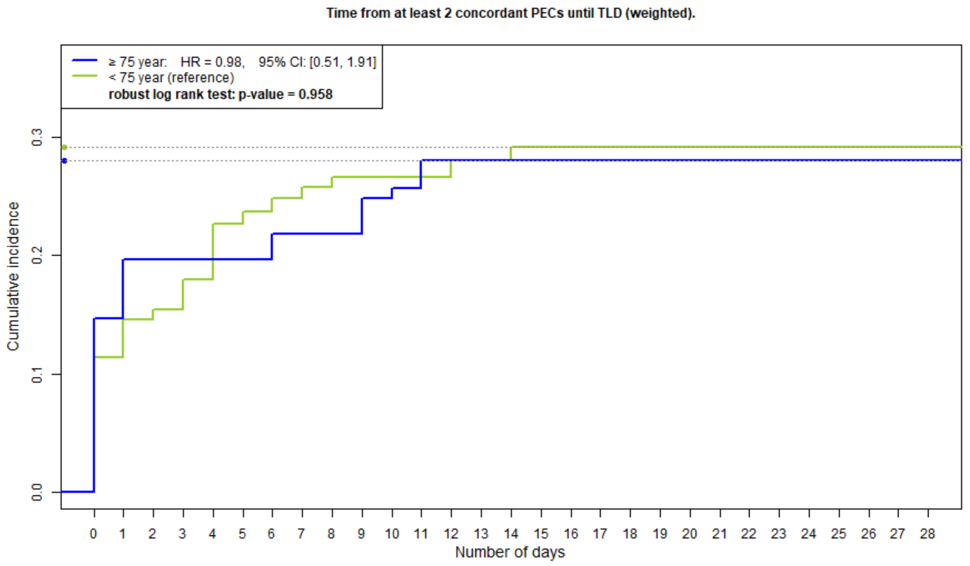

Figure 15: Comparison age subgroups – weighted cumulative incidence curves for time between receiving 2nd PEC and TLD.

9.3 Cancer type subgroups

The weighted cumulative incidence curves for the time from admission until receiving the 2nd PEC for the cancer type subgroups have been presented in Figure 16. If all patients had hematological cancer, 14.6 % would have received concordant PECs, this in contrast with only 9.2 % if no one had cancer and 11.6 % if all patients had a solid tumor. As the proportional hazard assumption was not met (see App Figure 16), Aalen's additive model was fitted instead. It could be concluded that the rate of receiving concordant PECs is significantly higher for patients with hematological cancer than for patients without cancer (p-value < 0.001).

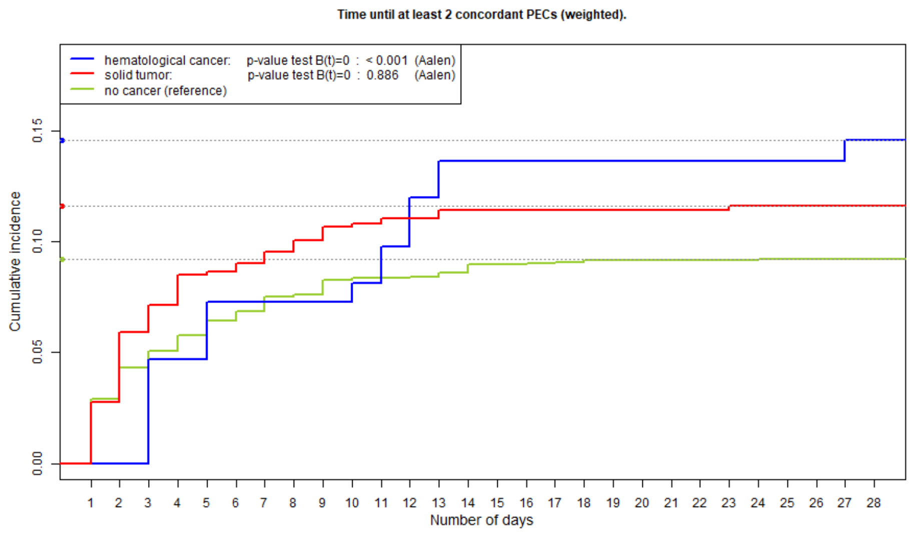

Figure 16: Comparison cancer type subgroups – weighted cumulative incidence curves for time from admission until receiving of 2nd PEC.

The weighted cumulative incidence curves for the time between receiving the 2nd PEC and TLD for the cancer type subgroups have been presented in Figure 17. If none of the patients had cancer, 31.1 % of the patients would have registered a TLD by the end of the study, 18.1 % if all patients had hematological cancer and 25.2 % if all patients had a solid tumor. No significant difference in the cause-specific hazard rate in TLD registration between the three subgroups was observed (p-value robust log rank test = 0.517, HR_{hem} = 0.53 [0.13, 2.21] and HR_{solid} = 0.74 [0.31, 1.74]).

These results also coincide with the results that were found based on the unweighted cumulative incidence curves.

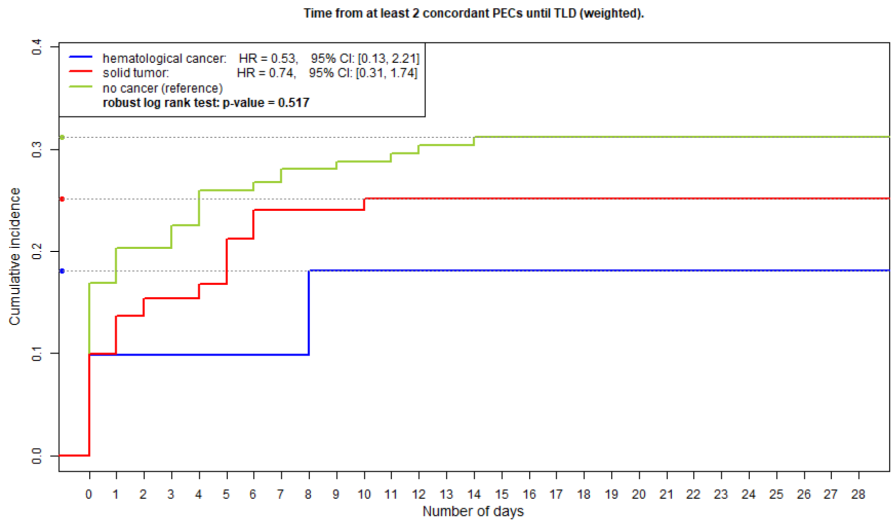

Figure 17: Comparison cancer type subgroups – weighted cumulative incidence curves for time between receiving 2nd PEC and TLD.

9.4 Cancer status subgroups

The weighted cumulative incidence curves for the time from admission until receiving the 2nd PEC for the cancer status subgroups have been presented in Figure 18. If all patients had active cancer, 20.4 % of them would have received concordant PECs by the end of the study. This in contrast with 8.9 % if none of the patients had cancer and 9.2 % if all patients had not active cancer. As the proportional hazard assumption was not met (see App Figure 19), Aalen's additive model was fitted instead. No significant difference in rate if receiving concordant PECs was detected (p-values = 0.088 and 0.234). However, when visually analysing the cumulative incidence curves, it appears that patients with active cancer receive concordant PECs more rapidly than patients without cancer or patients with not active cancer (which was also concluded based on the unweighted cumulative incidence curves).

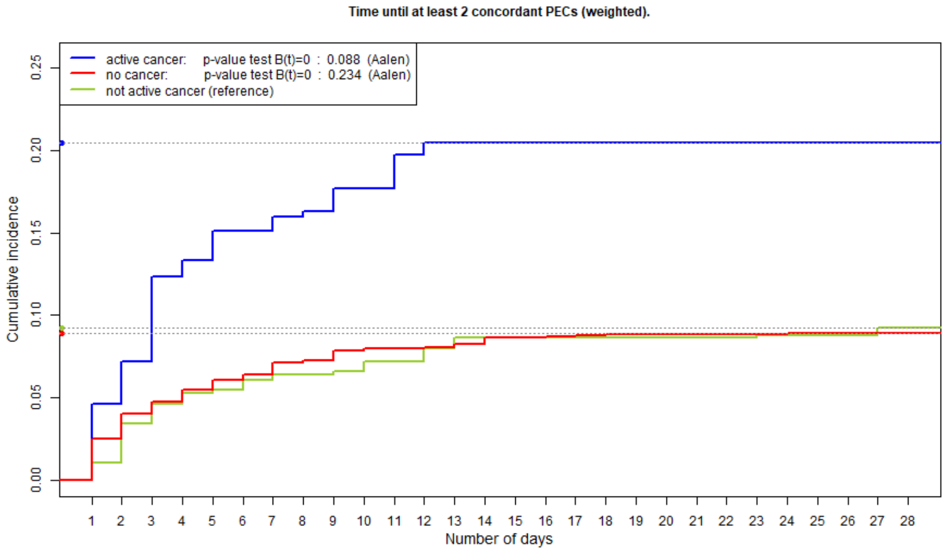

Figure 18: Comparison of cancer status subgroups – weighted cumulative incidence curves for time from admission until receiving of 2nd PEC.

The weighted cumulative incidence curves for the time between receiving the 2nd PEC and TLD has been presented in Figure 19. At the end of the study, 30.4 % would have registered a TLD if none of the patients had cancer, this in comparison with 28.7 % if everyone had active cancer and 23.4 % if everyone had not active cancer. No significant difference in the cause-specific hazard rate of TLD registration between the three subgroups has been observed (p-value robust log rank test = 0.718, HR_{active} = 1.36 [0.37, 4.96] and $HR_{no\ cancer}$ = 1.47 [0.53, 4.10]).

These results coincide with the results that were found based on the unweighted cumulative incidence curves.

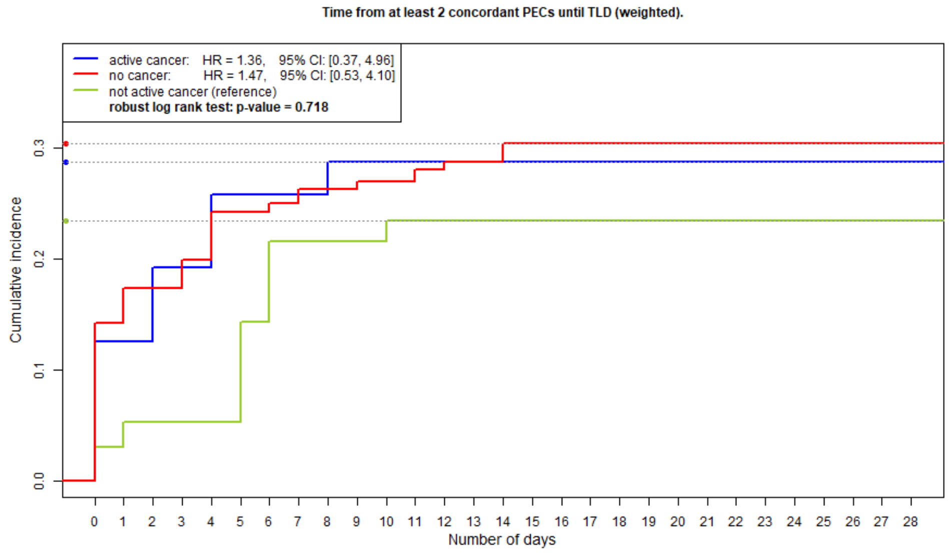

Figure 19: Comparison of cancer status subgroups – weighted cumulative incidence curves for time between receiving 2nd PEC and TLD.

9.5 Surgery subgroups

The weighted cumulative incidence curves for the time from admission until receiving the 2nd PEC for the surgery subgroups have been presented in Figure 20. At the end of the study, 6.6 % of the patients would have received concordant PECs if all patients had a scheduled surgery, this in comparison with 9.8 % if all patients had an unscheduled surgery and 9.2 % if none of the patients had a surgery. No significant difference in the cause-specific hazard rate of receiving concordant PECs between the three subgroups has been observed (p-value robust log rank test = 0.815, $HR_{no\ surgery}$ = 1.11 [0.53, 2.34] and $HR_{unscheduled}$ = 1.25 [0.56, 2.83]).

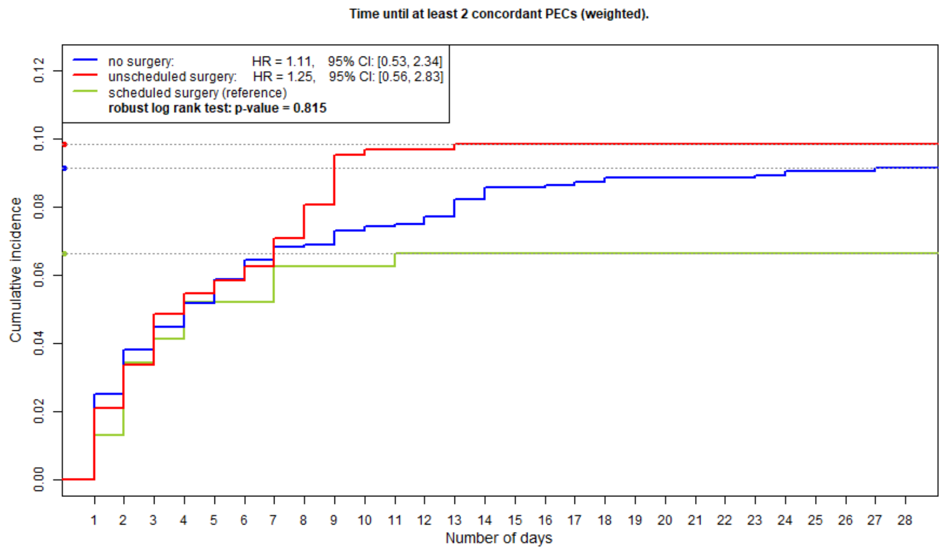

Figure 20: Comparison surgery subgroups – weighted cumulative incidence curves for time from admission until receiving of 2nd PEC.

The weighted cumulative incidence curves for the time between receiving the 2nd PEC and TLD have been presented in Figure 21. If all patients had a scheduled surgery, only 15.9 % of the patients would have registered a TLD by the end of the study. This in contrast with 25.2 % if none of the patients had surgery and 34.7 % if all patients had unscheduled surgery. No significant difference in cause-specific hazard rate of TLD registration between the three subgroups has been observed (p-value robust log rank test 0.371, $HR_{no\ surgery}$ = 1.96 [0.40, 9.52], $HR_{unscheduled}$ = 2.61 [0.48, 14.10]).

These results coincide with the results that were found based on the unweighted cumulative incidence curves.

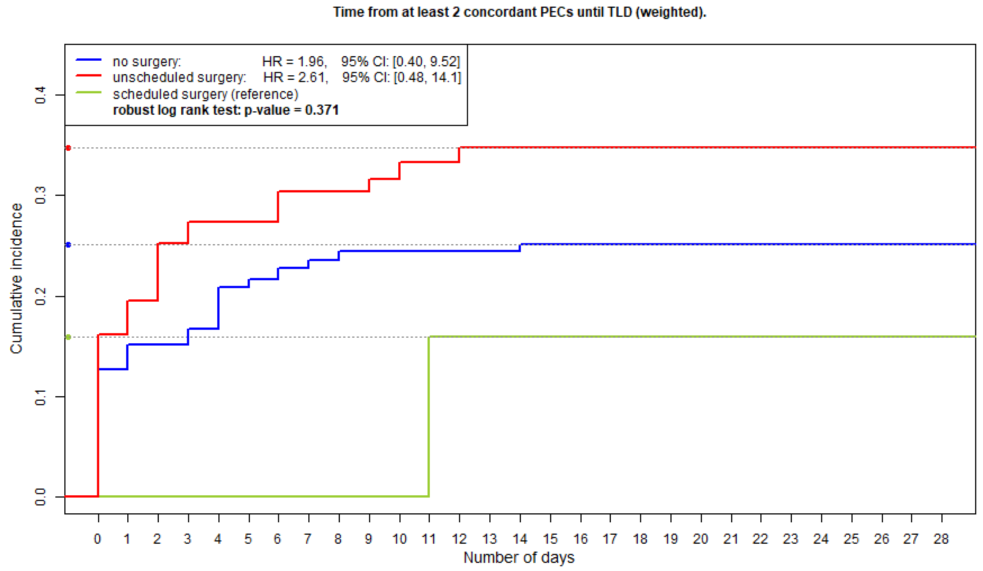

Figure 21: Comparison of surgery subgroups – weighted cumulative incidence curves for time between receiving 2nd PEC and TLD.

9.6 First indication of discrimination?

For the age and cancer type, a significant difference was detected in the cause-specific hazard rate of receiving concordant PECs, but no significant difference was observed in the cause-specific hazard rate of TLD registration. For the cancer status and surgery subgroups, no significant difference in cause-specific hazard rate of receiving PECs and of TLD registration was detected. Therefore, it can be concluded that the observations do not point towards discrimination (trend A was observed, but not trend B – see section 4.1).

9.7 Difference in mortality rate between subgroups

9.7.1 Age subgroups

The weighted cumulative incidence curves for the time between receiving the 2nd PEC and death or CEP for the age subgroups have been presented in Figure 22. If every young patient would have been replaced by an older patient with the same characteristics (same values for the predictors mentioned in Table 5), then 85.3 % of the patients would have reached CEP. Similarly, if every older patient would have been replaced by a younger patient with the same characteristics, then 88.8 % of the patients would have reached CEP. No significant difference in the mortality rate between the two age groups has been detected (p-value robust log rank test = 0.984 and HR = 1.00 [0.68, 1.47]). These results coincide with the results that were found based on the unweighted cumulative incidence curves.

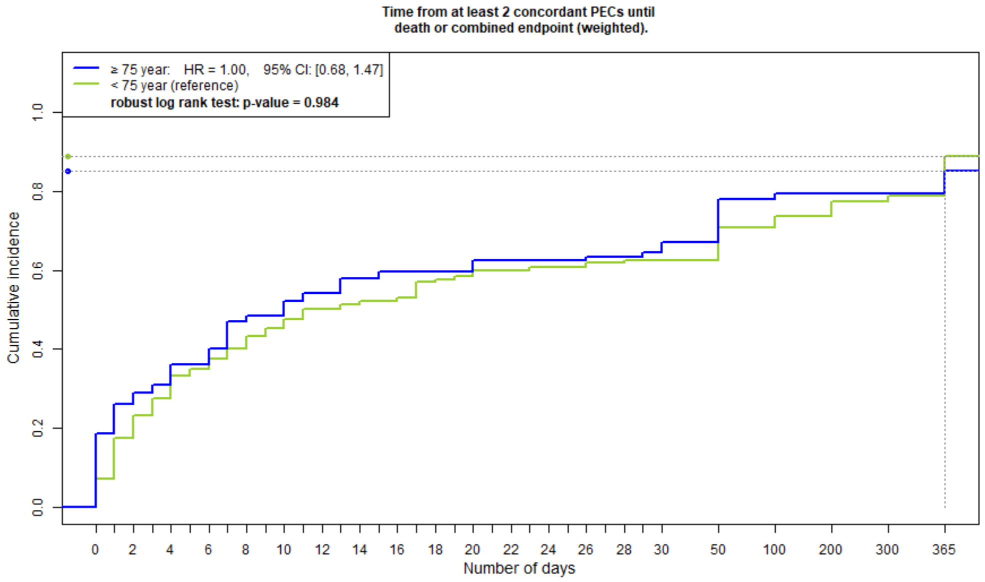

Figure 22: Comparison age subgroups – weighted cumulative incidence curves for time between receiving 2nd PEC and death or CEP.

9.7.2 Cancer type subgroups

The weighted cumulative incidence curves for the time between receiving the 2nd PEC and death or CEP for the cancer type subgroups have been presented in Figure 23. No significant difference in mortality rate between the three subgroups has been detected (p-value robust log rank test = 0.576, HR_{hem} = 1.20 [0.54, 2.68] and HR_{solid} = 0.82 [0.53, 1.27].

A remarkable result is that, if all patients had hematological cancer, 77.7 % of the patients would have already died within 10 days after receiving the 2nd PEC. This in contrast with only 51.0 % if none of the patients had cancer and 36.9 % if all patients had a solid tumor. After one year however, it appears that about 88 % of the patients would have reached CEP, no matter which subgroup is considered. These results coincide with the results that were found based on the unweighted cumulative incidence curves.

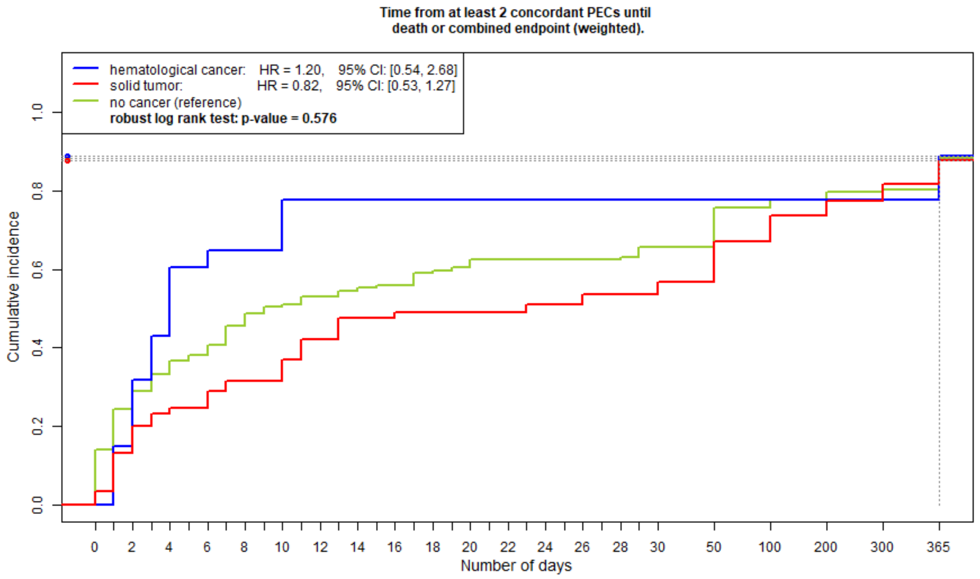

Figure 23: Comparison of cancer type subgroups – weighted cumulative incidence curves for time between receiving 2nd PEC death or CEP.

9.7.3 Cancer status subgroups

The weighted cumulative incidence curves for the time between receiving the 2nd PEC and death or CEP for the cancer type subgroups have been presented in Figure 24. At the end of the study, 88.3 % of the patients would have reached the combined endpoint if no one had cancer, 93.7 % if all patients had active cancer and 81.4 % if all patients had not active cancer. No significant difference in the mortality rate between the three subgroups has been detected (p-value robust log rank test = 0.858, HR_{active} = 1.19 [0.58, 2.42] and $HR_{no\ cancer}$ = 1.19 [0.64, 2.23]). These results coincide with the results that were found based on the unweighted cumulative incidence curves.

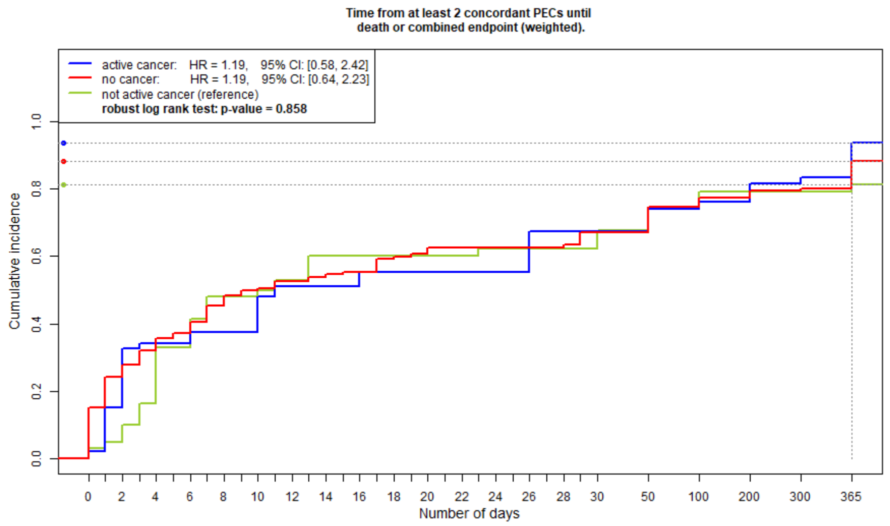

Figure 24: Comparison of cancer status subgroups – weighted cumulative incidence curves for time between receiving 2nd PEC death or CEP.

9.7.4 Surgery subgroups

The weighted cumulative incidence curves for the time between receiving the 2nd PEC and death or CEP for the surgery subgroups have been presented in Figure 25. If all patients had a scheduled surgery, only 62.6 % of the patients would have reached the combined endpoint after one year. This in contrast with 87.8 % if none of the patients had surgery and 90.9 % if all patients had an unscheduled surgery. The mortality rate for patients without surgery and patients with unscheduled surgery is respectively 2.63 [1.24, 5.57] and 2.79 [1.25, 6.22] times as large as the mortality rate of patients with a scheduled surgery. These results coincide with the results that were found based on the unweighted cumulative incidence curves.

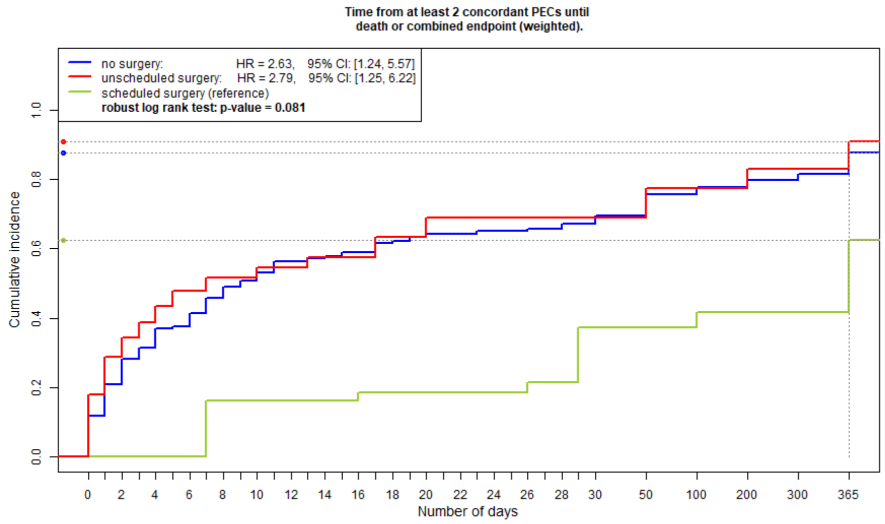

Figure 25: Comparison of surgery subgroups – weighted cumulative incidence curves for time between receiving 2nd PEC death or CEP.

10 Conclusion

Life supporting therapy should only be provided to patients at an intensive care unit (ICU) if the patients and their relatives are well informed about the treatment and associated risks and if the treatment intensity is proportional to the expected result. In this thesis, the focus was laid on possible overtreatment. A measure of overtreatment was the number of perceptions of excessive care (PEC) that patients received from clinicians. D.D. Benoit et al. (2018) showed that having at least two independent PECs (concordant PECs) is predictive of the patients' one-year outcome.

The main goal of this thesis was to study whether there was an indication of discrimination of subgroups of patients by clinicians. The following subgroups were studied: age subgroups (< 75 year old, ≥ 75 year old), cancer type subgroups (no cancer, hematological cancer, solid tumor), cancer status subgroups (no cancer, active cancer, not active cancer) and surgery subgroups (no surgery, scheduled surgery, unscheduled surgery). There is a hint of discrimination concerning the level of care if 1) there is a significant difference between subgroups in the proportion of patients with concordant PECs or in the rate of receiving those concordant PECs and 2) if there is a significant difference between subgroups in the proportion of patients with a treatment limitation decision (TLD) registrations or in the rate of TLD registration.

Univariate analysis showed that the proportion of patients who received concordant PECs is significantly higher in the subgroup of older patients and in the subgroups of patients with active cancer and that the proportion is significantly lower in the subgroup of patients with scheduled surgery in comparison to the other subgroups.

When studying the mortality (risk of death within 28 days, risk of death within 1 year and risk of reaching CEP) of patients with concordant PECs, no significant difference could be detected between the age, cancer type and cancer status subgroups. For the surgery subgroups, the risk of death within 28 days and death within 1 year was lower for patients with a scheduled surgery. When looking at the mortality of patients without concordant PECs (whose clinicians think the level of care is appropriate), it appeared that the mortality is significantly higher for older patients, for patients with hematological cancer, for patients with active cancer and for patients without surgery. This may be an indication that more patients in those subgroups should have received concordant PECs and the level of care may not have been appropriate.

Cumulative incidence curves were constructed for all subgroups for 1) the time from admission until receiving the 2nd PEC and 2) the time from receiving the 2nd PEC until treatment limitation decision (TLD) registration. By fitting cause-specific hazard models, hazard rates of different subgroups could be compared. Cumulative incidence curves for the time from receiving the 2nd PEC until death or combined endpoint were constructed as well (although these do not give extra information about possible discrimination). To adjust for background characteristics, inverse propensity score weighting was applied and weighted cumulative incidence curves were constructed. The propensity score was defined as the estimated conditional probability for a patient to belong to his own subgroup given the patient's characteristics.

Following things could be concluded based on the unweighted as well as on the weighted cumulative incidence curves. No significant difference in cause-specific hazard rate of TLD registration was detected between any of the subgroups. The cause-specific hazard rate of receiving concordant PECs was significantly higher for older patients and for patients with hematological cancer in comparison with the patients in the other subgroups. No significant difference in cause-specific hazard rate of receiving concordant PECs between the surgery subgroups was detected. Based on the unweighted cumulative incidence curves, the cause-specific hazard rate of receiving concordant PECs was significantly higher for patients with active cancer than for patients with not active cancer or patients without cancer. However, this difference was not detected based on the weighted cumulative incidence curves. Overall, it could be concluded that the observations do not point towards discrimination of patients based on age, cancer type, cancer status of surgery type.

One of the biggest limitations of this study is that no definite conclusion about discrimination can be formulated. Additional information will be necessary. In the current dataset, there is, for example, no information about cause of death of patients. It is unknown whether patients have died due to an unofficial treatment limitation decision or due to their underlying disease trajectory. If this information would be available, differences in the mortality rate between subgroups may also be seen as an indication of discrimination. Besides that, additional qualitative information (i.e. by interviewing clinicians, by asking the opinions of anthropologists…) would also be useful. It should also be mentioned that the number of patients with concordant PECs is probably underestimated as patients who were admitted prior to the study period and patients who remained in the ICU after the end of the study (and could have received concordant PECs during their unobserved ICU-stay) were

excluded from the analysis [2]. Another issue in this study was the reasonable amount of missing data (i.e. 339 out of the 1641 patients for whom it is unknown whether they reached the combined endpoint or not).

Finally, as no clear discriminatory attitude of clinicians was detected in this study, it is for further research (based on the same data) not necessary to make a distinction between the different types of subgroups.

11 References

[1] J. Teno, P. Gozalo, J. Bynum , N. Leland, S. Miller, N. Morden, T. Scupp, D. Goodman en V. Mor, „Change in end-of-life care for medicare beneficiaries. Site of death, place of care, and health care transtitions in 2001 2005, and 2009.," *Jama,* nr. 309, pp. 470-477, 2013.

[2] D. D. Benoit, H. I. Jensen, J. Malmgren, V. Metaxa, A. K. Reyners, M. Darmon, K. Rusinova, D. Talmor, A. P. Meert, L. Calcelliere, L. Zubek, P. Maia, A. Michalsen, S. Vanheule, E. J. O. Kompanje, J. Decruyenaere, S. Vandenberghe, S. Vansteelandt, B. Gadeyne, B. Van den Bulcke, E. Azoulay and R. D. Piers, "Outcome in patients perceived as receiving excessive care across different ethical climates: a prospective study in 68 intensive care units in Europe and the USA," *Intensive Care Med.,* pp. 1039-1049, 28 May 2018.

[3] D. G. Kleinbaum en M. Klein, Survival Analysis - A Self-Learning Text, 3th red., New York: Springer Science+Business Media, 1996.

[4] D. Jackson, Ian R. White, Shaun Seaman, Hannah Evans, Kathy Baisley en James Carpenter, „Relaxing the independent censoring assumption in the Cox proportional hazards model using multiple imputation," *Statistics in Medicine,* nr. 33, p. 4681–4694, 2014.

[5] J. Fox and S. Weisberg, "Cox Proportional-Hazards Regression for Survival Data in R," Faculty of Social Sciences, McMaster University, 2018.

[6] A. Kassambara, "Cox Proportional-Hazards Model," Statistical tools for high-throughput data analysis, [Online]. Available: http://www.sthda.com/english/wiki/cox-proportional-hazards-model. [Accessed 15 November 2018].

[7] X. Liu, Survival analysis: models and applications, vol. 6.6.2 Schoenfeld residuals, USA: Wiley, 2012.

[8] S. Park and D. . J. Hendry, "Reassessing Schoenfeld residual tests of proportional hazards in political science event history analyse.," *American Journal of Political Science*, no. Vol. 59, No. 4, pp. 1072-1087, 2015.

[9] D. W. Hosmer en P. Royston, „Using Aalen's linear hazards model to investigate time-varying effects in the proportional hazards regression model," *The Stata Journal (2002) 2,Number 4, pp. 331–350,* nr. Number 4, pp. 331-350, 2002.

[10] S. R. Hinchliffe, “Competing Risks - What, Why, When and How?,” Department of Health Sciences, University of Leicester, 2012.

[11] H. Putter, M. Fiocco and R. Geskus, “Tutorial in biostatistics: Competing risks and multi-state models,” *Wiley InterScience,* no. 26, pp. 2389-2430, 2007.

[12] L. Tian and R. O. Olshen, “Survival Analysis: Logrank Test,” Stanford Universty.

[13] L. Tian and R. Olshen , “STAT331 - Logrank test,” [Online]. Available: https://web.stanford.edu/~lutian/coursepdf/unitweek3.pdf. [Accessed 21 January 2019].

[14] D. B. Rubin, “Estimating causal effects from large data sets using propensity scores,” *Annals of internal medicine,* no. 127 (8 Pt 2), pp. 757-763, 1997.

[15] Y. Abdia, K. B. Kulasekera, S. Datta, M. Boakye and M. Kong, “Propensity scores based methods for estimating average treatment effect and average treatment effect among treated: a comparative study,” *Biometric Journal 59,* no. 5, pp. 967-985, 2017.

[16] M. A. Hernan and J. M. Robins, “Estimating causal effects from epidemiological data,” *Community Health,* no. 60, pp. 578-586, 2006.

[17] F. Thoemmes en A. D. Ong, „A primer on inverse probability of treatment weighting and marginal structure models.,” *Sage,* nr. Vol 4., pp. 40-59, 2015.

[18] S. Greenland en J. M. Robins, „Identifiability, Exchangeability and Epidemiological Confounding,” *International Journal of Epidemiology,* nr. Vol. 15, No. 3, pp. 413-419, 1988.

[19] J. Lessler, B. K. Lee and E. A. Stuart , “Weight Trimming and Propensity Score Weighting,” 31 March 2011. [Online]. Available: https://doi.org/10.1371/journal.pone.0018174. [Accessed 2 January 2019].

A Appendix

A.1 Selection bias in average$^{(-)}$ ethical climate

App Table 1: Comparison of ICU mortality and length of stay between ethical climates [2]

	Ethical climate				
	Good	Average +	Average -	Poor	p-value
ICU mortality	n=178	n=415	n=82	n=541	
	7.90%	8.00%	22.00%	8.70%	0.001*
Median length of stay in days (IQR)	n=176	n=414	n=82	n=536	
	1.2 (0.8-3.1)	1.2 (0.8-3.1)	3.1 (1.5-11.2)	1.6 (0.9-1.6)	< 0.0001
*Pearson Chi-Square, ** Non-parametric test comparing medians					

A.2 Univariate description

A.2.1 Comparing country, hospital, ICU and patient characteristics

App Table 2: Differences in characteristics between age subgroups (part 1).

		< 75 year old (1236 patients)			≥ 75 year old (405 patients)			Test results: p-value	
**Country characteristics**		**number**	**percentage**	**median** (25th - 75th percentile)	**number**	**percentage**	**median** (25th - 75th percentile)	**Wilcoxon rank sum test**	**x^2-test / Fisher's exact test**
Number of ICU beds / 100.000 inhabitants				11.6 (6.4 - 15.9)			12.5 (6.7 - 15.9)	0.00005973	
Geographical region									0.2935
	Central Europe	146	11.8%		56	13.8%			
	Northern Europe	217	17.6%		74	18.3%			
	Southern Europe	97	7.8%		40	9.9%			
	Western Europe / USA	776	62.8%		235	58.0%			
**Hospital characteristics**		**number**	**percentage**	**median** (25th - 75th percentile)	**number**	**percentage**	**median** (25th - 75th percentile)	**Wilcoxon rank sum test**	**x^2-test / Fisher's exact test**
Hospital type									0.0063
	Public	267	21.6%		118	29.1%			
	Private	66	5.3%		28	6.9%			
	University affiliated	195	15.8%		54	13.3%			
	University	708	57.3%		205	50.6%			
Total beds in hospital									0.0383
	<250	89	7.2%		33	8.1%			
	250-499	254	20.6%		108	26.7%			
	500-749	319	25.8%		102	25.2%			
	>750	574	46.4%		162	40.0%			
**ICU characteristics**		**number**	**percentage**	**median** (25th - 75th percentile)	**number**	**percentage**	**median** (25th - 75th percentile)	**Wilcoxon rank sum test**	**x^2-test / Fisher's exact test**
Ethical climate									0.2634
	Good	249	20.1%		72	17.8%			
	Average +	531	43.0%		166	41.0%			
	Poor	456	36.9%		167	41.2%			
Number of beds per ICU				14 (10 - 24)			13 (9 - 24)	0.1547	
Percentage of population over 65 year in ICU[b]				18 (18 - 18)			18 (18 - 18)		6.0E-05
Patient to nurse ratio				2 (1.4 - 3)			2 (1.4 - 3)	0.2763	
Patient to junior physician ratio				4 (3 - 6)			4 (3 - 6)	0.9552	
Patient to senior physician ratio				7 (5 - 8)			7 (5 - 8)	0.6804	

[b]variable considered as categorical because of the limited number of unique values

App Table 3: Differences in characteristics between age subgroups (part 2).

Patient characteristics		< 75 year old (1236 patients)			≥ 75 year old (405 patients)			Test results: p-value	
		number	percentage	median (25th - 75th percentile)	number	percentage	median (25th - 75th percentile)	Wilcoxon rank sum test	X^2-test / Fisher's exact test
Age				59 (47 - 67)			80 (77 - 84)	< 2.2 E-16	
Gender (male)		749	60.6%		221	54.6%			0.03714
ECOG performance status									1.9E-06
	Grade 0 (full functional)	472	38.2%		101	24.9%			
	Grade 1 (symptomatic)	303	24.5%		101	24.9%			
	Grade 2 (functional but not able to work)	160	12.9%		80	19.8%			
	Grade 3 (limited functionality)	136	11.0%		68	16.8%			
	Grade 4 (bedridden)	58	4.7%		24	5.9%			
	Unknown	107	8.7%		31	7.7%			
Nursing home resident		50	4.0%		28	6.9%			0.0264
Moderate to severe comorbidities (number)									2.7E-05
	0	608	49.2%		182	44.9%			
	1	514	41.6%		152	37.5%			
	≥ 2	114	9.2%		71	17.5%			
Type comorbidity									
	Solid tumor	219	17.7%		85	21.0%			0.1627
	Heart failure (NYHA III or IV)	109	8.8%		83	20.5%			4.0E-10
	COPD (Gold III or IV or equivalent)	131	10.6%		59	14.6%			0.0378
	Neurological (excluding dementia)	79	6.4%		24	5.9%			0.8280
	Hematological malignancy	80	6.5%		12	3.0%			0.0111
	Liver cirrhosis (Child Pugh B or C)	77	6.2%		5	1.2%			0.0001
	Chronic renal failure requiring dialysis	39	3.2%		15	3.7%			0.7066
	Dementia (moderate or severe)	16	1.3%		23	5.7%			1.3E-06
	AIDS	14	1.1%		0	0.0%			0.0278
Abuse									
	Alcohol	169	13.7%		12	3.0%			4.1E-09
	Active smoking	264	21.4%		26	6.4%			1.3E-11
Main admission reason(s)									
	Respiratory failure	287	23.2%		103	25.4%			0.4007
	Sepsis / severe sepsis / septic shock	252	20.4%		71	17.5%			0.2367
	Heart failure / cardiogenic shock	186	15.0%		93	23.0%			0.0003
	Neurologic pathology / Stroke / ICB	141	11.4%		41	10.1%			0.5332
	Gastro-intestinal pathology / liver failure	133	10.8%		36	8.9%			0.3264
	Metabolic / renal	112	9.1%		32	7.9%			0.5385
	Multiple trauma	83	6.7%		13	3.2%			0.0129
	Head trauma	41	3.3%		17	4.2%			0.4980
Surgery within 48 hrs		403	32.6%		157	38.8%			0.0272
Surgery									0.0181
	No surgery	827	66.9%		243	60.0%			
	Scheduled surgery	142	11.5%		65	16.0%			
	Unscheduled surgery	267	21.6%		97	24.0%			
Do-not-resuscitate order before admission									0.0007
	Full code	1113	90.0%		350	86.4%			
	Unknown	61	4.9%		14	3.5%			
	No CPR	40	3.2%		21	5.2%			
	Withholding of therapy	22	1.8%		20	4.9%			
Severity of illness < 24 hrs after admission									
	Invasive mechanical ventilation	604	48.9%		180	44.4%			0.1364
	Vasopressor need	427	34.5%		156	38.5%			0.1647
	Dialysis	41	3.3%		14	3.5%			1
Written withholding / withdrawing order < 24 h		38	3.1%		29	7.2%			0.0005
Characteristics during ICU stay (non missing values)[a]		n = 1137			n = 374				
	Invasive mechanical ventilation	675	59.4%		198	52.9%			0.0338
	Duration of invasive ventilation (days)			1 (0 - 3)			1 (0 - 2)	0.01068	
	Vasopressor need	536	47.1%		180	48.1%			0.7858
	Duration of vasopressors (days)			0 (0-2)			0 (0-2)	0.9051	
	Dialysis	84	7.4%		39	10.4%			0.0791
	Duration of dialysis (days)			0 (0 - 0)			0 (0 - 0)	0.07208	

[a]Percentages are related to the number of non-missing values

App Table 4: Differences in characteristics between cancer type subgroups (part 1).

		No cancer (1254 patients)			Solid tumor (299 patients)			Hematological cancer (88 patients)			Test results: p-value	
**Country characteristics**		**number**	**percentage**	**median** (25th - 75th percentile)	**number**	**percentage**	**median** (25th - 75th percentile)	**number**	**percentage**	**median** (25th - 75th percentile)	**Kruskal-Wallis test**	**X^2-test / Fisher's exact test**
Number of ICU beds / 100.000 inhabitants				11.6 (6.4 - 15.9)			11.6 (6.4 - 15.9)			11.6 (11.6 - 15.9)	0.4883	
Geographical region												0.0005
	Central Europe	147	11.7%		50	16.7%		5	5.7%			
	Northern Europe	257	20.5%		31	10.4%		3	3.4%			
	Southern Europe	98	7.8%		29	9.7%		10	11.4%			
	Western Europe / USA	752	60.0%		189	63.2%		70	79.5%			
**Hospital characteristics**		**number**	**percentage**	**median** (25th - 75th percentile)	**number**	**percentage**	**median** (25th - 75th percentile)	**number**	**percentage**	**median** (25th - 75th percentile)	**Kruskal-Wallis test**	**X^2-test / Fisher's exact test**
Hospital type												0.0045
	Public	318	25.4%		58	19.4%		9	10.2%			
	Private	77	6.1%		14	4.7%		3	3.4%			
	University affiliated	178	14.2%		52	17.4%		19	21.6%			
	University	681	54.3%		175	58.5%		57	64.8%			
Total beds in hospital												0.0077
	<250	85	6.8%		28	9.4%		9	10.2%			
	250-499	271	21.6%		75	25.1%		16	18.2%			
	500-749	336	26.8%		54	18.1%		31	35.2%			
	>750	562	44.8%		142	47.5%		32	36.4%			
**ICU characteristics**		**number**	**percentage**	**median** (25th - 75th percentile)	**number**	**percentage**	**median** (25th - 75th percentile)	**number**	**percentage**	**median** (25th - 75th percentile)	**Kruskal-Wallis test**	**X^2-test / Fisher's exact test**
Ethical climate												1.9E-06
	Good	241	19.2%		45	15.1%		35	39.8%			
	Average +	552	44.0%		119	39.8%		26	29.5%			
	Poor	461	36.8%		135	45.2%		27	30.7%			
Number of beds per ICU				14 (10 - 24)			14 (10 - 25)			12 (8 - 14)	0.00936	
Percentage of population over 65 year in ICU[b]				18 (18 - 18)			18 (18 - 18)			18 (18 - 18)		0.0005
Patient to nurse ratio				2 (1.3 - 3)			2 (1.4 - 2)			2 (2 - 3)	0.0002594	
Patient to junior physician ratio				4 (3 - 6)			4 (3 - 6)			4 (3.75 - 6)	0.2052	
Patient to senior physician ratio				7 (4 - 8)			7 (5 - 8)			7 (6 - 8)	0.2187	

[b]variable considered as categorical because of the limited number of unique values

App Table 5: Differences in characteristics between cancer type subgroups (part 2).

Patient characteristics		No cancer (1254 patients)			Solid tumor (299 patients)			Hematological cancer (88 patients)			Test results: p-value	
		number	percentage	median (25th - 75th percentile)	number	percentage	median (25th - 75th percentile)	number	percentage	median (25th - 75th percentile)	Kruskal-Wallis test	χ^2-test / Fisher's exact test
Age (continuous)				63 (18 - 74)			68 (59 -75)			63 (53.75 - 70.25)	0.0001	
Age (categorical: 75 years or older)		308	24.6%		85	28.4%		12	13.6%			0.0179
Gender (male)		736	58.7%		187	62.5%		47	53.4%			0.5553
ECOG performance status												0.0005
	Grade 0 (full functional)	475	33.9%		83	27.8%		15	17.0%			
	Grade 1 (symptomatic)	285	22.7%		88	29.4%		31	35.2%			
	Grade 2 (functional but not able to work)	169	13.5%		50	16.7%		21	23.9%			
	Grade 3 (limited functionality)	156	12.4%		38	12.7%		10	11.4%			
	Grade 4 (bedridden)	57	4.5%		21	7.0%		4	4.5%			
	Unknown	112	8.9%		19	6.3%		7	8.0%			
Nursing home resident		69	5.5%		7	2.3%		2	2.3%			0.0380
Moderate to severe comorbidities (number)												0.0005
	0	790	63.0%		0	0.0%		0	0.0%			
	1	379	30.2%		216	72.2%		71	80.7%			
	≥ 2	85	6.8%		83	27.8%		17	19.3%			
Type comorbidity												
	Heart failure (NYHA III or IV)	166	13.2%		22	7.4%		4	4.5%			0.0020
	COPD (Gold III or IV or equivalent)	143	11.4%		42	14.0%		5	5.7%			0.0905
	Neurological (excluding dementia)	89	7.1%		13	4.3%		1	1.1%			0.0220
	Liver cirrhosis (Child Pugh B or C)	72	5.7%		9	3.0%		1	1.1%			0.0410
	Chronic renal failure requiring dialysis	46	3.7%		5	1.7%		3	3.4%			0.2204
	Dementia (moderate or severe)	32	2.6%		6	2.0%		1	1.1%			0.7886
	AIDS	13	1.0%		1	0.3%		0	0.0%			0.0005
Abuse												
	Alcohol	166	13.2%		13	4.3%		2	2.3%			0.0005
	Active smoking	241	19.2%		48	16.1%		1	1.1%			0.0005
Main admission reason(s)												
	Respiratory failure	289	23.0%		72	28.1%		29	33.0%			0.1067
	Sepsis / severe sepsis / septic shock	222	17.7%		56	18.0%		45	51.1%			2.1E-13
	Heart failure / cardiogenic shock	244	19.5%		26	8.7%		9	10.2%			1.1E-05
	Neurologic pathology / Stroke / ICB	157	12.5%		18	6.0%		7	8.0%			0.0036
	Gastro-intestinal pathology / liver failure	133	10.6%		32	10.7%		4	4.5%			0.1764
	Metabolic / renal	108	8.6%		25	8.4%		11	12.5%			0.4425
	Multiple trauma	92	7.3%		4	1.3%		0	0.0%			0.0005
	Head trauma	55	4.4%		3	1.0%		0	0.0%			0.0005
Surgery within 48 hrs		400	31.9%		152	50.8%		8	9.1%			1.0E-14
Surgery												< 2.2 E-16
	No surgery	850	67.8%		140	46.8%		80	90.9%			
	Scheduled surgery	111	8.9%		93	31.1%		3	3.4%			
	Unscheduled surgery	293	23.4%		66	22.1%		5	5.7%			
Do-not-resuscitate order before admission												0.0035
	Full code	1132	90.3%		251	83.9%		80	90.9%			
	Unknown	57	4.5%		14	4.7%		4	4.5%			
	No CPR	34	2.7%		24	8.0%		3	3.4%			
	Withholding of therapy	31	2.5%		0	3.3%		1	1.1%			
Severity of illness < 24 hrs after admission												
	Invasive mechanical ventilation	616	49.1%		141	47.2%		27	30.7%			0.0036
	Vasopressor need	443	35.3%		116	38.8%		24	27.3%			0.1330
	Dialysis	47	3.7%		7	2.3%		1	1.1%			0.2909
Written withholding / withdrawing order < 24 h		50	4.0%		17	5.7%		0	0.0%			< 2.2 E-16
Characteristics during ICU stay (non missing values)[a]		n = 1165			n = 273			n = 73				
	Invasive mechanical ventilation	684	58.7%		154	56.4%		35	47.9%			0.1721
	Duration of invasive ventilation (days)			1 (0 - 3)			1 (0 - 2)			0 (0 - 4)	0.6545	
	Vasopressor need	544	46.7%		137	50.2%		35	47.9%			0.5802
	Duration of vasopressors (days)			0 (0 - 2)			1 (0 - 2)			0 (0 - 2)	0.4679	
	Dialysis	97	8.3%		17	6.2%		9	13.3%			0.2120
	Duration of dialysis (days)			0 (0 - 0)			0 (0 - 0)			0 (0 - 0)	0.1903	

[a]Percentages are related to the number of non-missing values

App Table 6: Differences in characteristics between cancer status subgroups (part 1).

		No cancer (1254 patients)			Active cancer (117 patients)			Not active cancer (270 patients)			Test results: p-value	
**Country characteristics**		number	percentage	median (25th - 75th percentile)	number	percentage	median (25th - 75th percentile)	number	percentage	median (25th - 75th percentile)	Kruskal-Wallis test	X²-test / Fisher's exact test
Number of ICU beds / 100.000 inhabitants				11.6 (6.4 - 15.9)			13.8 (11.6- 15.9)			11.6 (6.4 - 15.9)	0.0013	
Geographical region												0.0005
	Central Europe	147	11.7%		15	12.8%		40	14.8%			
	Northern Europe	257	20.5%		7	6.0%		27	10.0%			
	Southern Europe	98	7.8%		7	6.0%		32	11.9%			
	Western Europe / USA	752	60.0%		88	75.2%		171	63.3%			
**Hospital characteristics**		number	percentage	median (25th - 75th percentile)	number	percentage	median (25th - 75th percentile)	number	percentage	median (25th - 75th percentile)	Kruskal-Wallis test	X²-test / Fisher's exact test
Hospital type												0.0020
	Public	318	25.4%		20	17.1%		47	17.4%			
	Private	77	6.1%		10	8.5%		7	2.6%			
	Universityaffiliated	178	14.2%		28	23.9%		43	15.9%			
	University	681	54.3%		59	50.4%		173	64.1%			
Total beds in hospital												0.0002
	<250	85	6.8%		20	17.1%		17	6.3%			
	250-499	271	21.6%		33	28.2%		58	21.5%			
	500-749	336	26.8%		15	12.8%		70	25.9%			
	>750	562	44.8%		49	41.9%		125	46.3%			
**ICU characteristics**		number	percentage	median (25th - 75th percentile)	number	percentage	median (25th - 75th percentile)	number	percentage	median (25th - 75th percentile)	Kruskal-Wallis test	X²-test / Fisher's exact test
Ethical climate												0.1261
	Good	241	19.2%		26	22.2%		54	20.0%			
	Average +	552	44.0%		48	41.0%		97	35.9%			
	Poor	461	36.8%		43	36.8%		119	44.1%			
Number of beds per ICU				14 (10 - 24)			12 (8 - 22)			12.5 (10 - 24.75)	0.0182	
Percentage of population over 65 year in ICU[b]				18 (18 - 18)			18 (18 - 18)			18 (18 - 18)		0.0005
Patient to nurse ratio				2 (1.3 - 3)			2 (2 - 2)			2 (1.4 - 3)	0.0409	
Patient to junior physician ratio				4 (3 - 6)			5 (3 - 7)			4 (3 - 6)	0.0855	
Patient to senior physician ratio				7 (4 - 8)			7 (6 - 8)			7 (5 - 8)	0.0825	

[b]variable considered as categorical because of the limited number of unique values

App Table 7: Differences in characteristics between cancer status subgroups (part 2).

Patient characteristics		No cancer (1254 patients)			Active cancer (117 patients)			Not active cancer (270 patients)			Test results: p-value	
		number	percentage	median (25th - 75th percentile)	number	percentage	median (25th - 75th percentile)	number	percentage	median (25th - 75th percentile)	Kruskal-Wallis test	X^2-test / Fisher's exact test
Age (continuous)				63 (50 - 74)			66 (58 - 73)			67 (57 - 75)	0.002449	
Age (categorical: 75 years or older)		308	24.6%		24	20.5%		73	27.0%			0.3849
Gender (male)		736	58.7%		63	53.8%		171	63.3%			0.1805
ECOG performance status												0.0005
	Grade 0 (full functional)	475	37.9%		20	17.1%		78	28.9%			
	Grade 1 (symptomatic)	285	22.7%		30	25.6%		89	33.0%			
	Grade 2 (functional but not able to work)	169	13.5%		26	22.2%		45	16.7%			
	Grade 3 (limited functionality)	156	12.4%		21	17.9%		27	10.0%			
	Grade 4 (bedridden)	57	4.5%		9	7.7%		16	5.9%			
	Unknown	112	8.9%		11	9.4%		15	5.6%			
Nursing home resident		69	5.5%		3	2.6%		6	2.2%			0.0340
Moderate to severe comorbidities (number)												0.0005
	0	790	63.0%		0	0.0%		0	0.0%			
	1	379	30.2%		90	76.9%		197	73.0%			
	≥ 2	85	6.8%		27	23.1%		73	23.0%			
Type comorbidity												
	Heart failure (NYHA III or IV)	166	13.2%		5	4.3%		21	7.8%			0.0005
	COPD (Gold III or IV or equivalent)	143	11.4%		12	10.3%		35	13.0%			0.6898
	Neurological (excluding dementia)	89	7.1%		7	6.0%		7	2.6%			0.0215
	Liver cirrhosis (Child Pugh B or C)	72	5.7%		0	0.0%		10	3.7%			< 2.2E-16
	Chronic renal failure requiring dialysis	46	3.7%		4	3.4%		4	1.5%			0.1629
	Dementia (moderate or severe)	32	2.6%		1	0.9%		6	2.2%			0.6687
	AIDS	13	1.0%		0	0.0%		1	0.4%			< 2.2E-16
Abuse												
	Alcohol	166	13.2%		1	0.9%		14	5.2%			0.0005
	Active smoking	241	19.2%		10	8.5%		39	14.4%			0.0048
Main admission reason(s)												
	Respiratory failure	289	23.0%		36	30.8%		65	24.1%			0.1704
	Sepsis / severe sepsis / septic shock	222	17.7%		34	29.1%		67	24.8%			0.0009
	Heart failure / cardiogenic shock	244	19.5%		11	9.4%		24	8.9%			1.2E-05
	Neurologic pathology / Stroke / ICB	157	12.5%		8	6.8%		17	6.3%			0.0020
	Gastro-intestinal pathology / liver failure	133	10.6%		10	8.5%		26	9.6%			0.7234
	Metabolic / renal	108	8.6%		14	12.0%		22	8.1%			0.4356
	Multiple trauma	92	7.3%		0	0.0%		4	1.5%			0.0005
	Head trauma	55	4.4%		1	0.9%		2	0.7%			0.0025
Surgery within 48 hrs		100	31.9%		38	32.5%		122	45.2%			0.0002
Surgery												< 2.2E-16
	No surgery	850	67.8%		79	67.5%		141	52.2%			
	Scheduled surgery	111	8.9%		19	16.2%		77	28.5%			
	Unscheduled surgery	293	23.4%		19	16.2%		52	19.3%			
Do-not-resuscitate order before admission												0.0005
	Full code	1132	90.3%		87	74.4%		224	90.4%			
	Unknown	57	4.5%		8	6.8%		10	3.7%			
	No CPR	34	2.7%		14	12.0%		13	4.8%			
	Withholding of therapy	31	2.5%		8	6.8%		3	1.1%			
Severity of illness < 24 hrs after admission												
	Invasive mechanical ventilation	616	49.1%		42	35.9%		126	46.7%			0.0217
	Vasopressor need	443	35.3%		34	29.1%		106	39.3%			0.1495
	Dialysis	47	3.7%		3	2.6%		5	1.9%			0.3053
Written withholding / withdrawing order < 24 h		50	4.0%		11	9.4%		6	2.2%			0.0080
Characteristics during ICU stay (non missing values)[a]		n = 1165			n = 93			n = 253				
	Invasive mechanical ventilation	684	58.7%		46	49.5%		143	56.5%			0.2002
	Duration of invasive ventilation (days)			1 (0 - 3)			0 (0 - 3)			1 (0 - 3)	0.5583	
	Vasopressor need	544	46.7%		42	45.2%		130	51.4%			0.3626
	Duration of vasopressors (days)			0 (0 - 2)			0 (0 - 2)			1 (0 - 2)	0.4729	
	Dialysis	97	8.3%		5	5.4%		21	8.3%			0.6702
	Duration of dialysis (days)			0 (0 - 0)			0 (0 - 0)			0 (0 - 0)	0.6219	

[a]Percentages are related to the number of non-missing values

App Table 8: Differences in characteristics between surgery subgroups (part 1).

		No surgery (1070 patients)			Scheduled surgery (207 patients)			Unscheduled surgery (364 patients)			Test results: p-value	
**Country characteristics**		**number**	**percentage**	**median (25th - 75th percentile)**	**number**	**percentage**	**median (25th - 75th percentile)**	**number**	**percentage**	**median (25th - 75th percentile)**	**Kruskal-Wallis test**	**X^2-test / Fisher's exact test**
Number of ICU beds / 100.000 inhabitants				11.6 (6.4 - 15.9)			11.6 (6.4 - 15.9)			11.6 (6.4 - 15.9)	0.3832	
Geographical region												1.0E-13
	Central Europe	95	8.9%		49	23.7%		58	15.9%			
	Northern Europe	214	20.0%		19	9.2%		58	15.9%			
	Southern Europe	71	6.6%		14	6.8%		52	14.3%			
	Western Europe / USA	690	64.5%		125	60.4%		196	53.8%			
**Hospital characteristics**		**number**	**percentage**	**median (25th - 75th percentile)**	**number**	**percentage**	**median (25th - 75th percentile)**	**number**	**percentage**	**median (25th - 75th percentile)**	**Kruskal-Wallis test**	**X^2-test / Fisher's exact test**
Hospital type												0.0005
	Public	273	25.5%		23	11.1%		89	24.5%			
	Private	74	6.9%		2	1.0%		18	4.9%			
	Universityaffiliated	185	17.3%		22	10.6%		42	11.5%			
	University	538	50.3%		160	77.3%		215	59.1%			
Total beds in hospital												8.9E-08
	<250	99	9.3%		2	1.0%		21	5.8%			
	250-499	234	21.9%		43	20.8%		85	23.4%			
	500-749	299	27.9%		35	16.9%		87	23.9%			
	>750	438	40.9%		127	61.4%		171	47.0%			
**ICU characteristics**		**number**	**percentage**	**median (25th - 75th percentile)**	**number**	**percentage**	**median (25th - 75th percentile)**	**number**	**percentage**	**median (25th - 75th percentile)**	**Kruskal-Wallis test**	**X^2-test / Fisher's exact test**
Ethical climate												< 2.2 E-16
	Good	258	24.1%		25	12.1%		38	10.4%			
	Average +	483	45.1%		56	27.1%		158	43.4%			
	Poor	329	30.7%		126	60.9%		168	46.2%			
Number of beds per ICU				13 (10 - 22)			24 (11 - 44)			14 (10 - 40)	1.378E-11	
Percentage of population over 65 year in ICU[b]				18 (18 - 18)			18 (18 - 18)			18 (18 - 18)		0.0005
Patient to nurse ratio				2 (1.4 - 3)			2 (1.4 - 3)			2 (1.375 - 2)	0.06405	
Patient to junior physician ratio				4 (3 - 6)			4 (3 - 6)			4 (3 - 6)	0.7581	
Patient to senior physician ratio				6 (5 - 8)			7 (5 - 8)			7 (5 - 8)	0.5132	

[b]variable considered as categorical because of the limited number of unique values

App Table 9: Differences in characteristics between surgery subgroups (part 2).

Patient characteristics		No surgery (1070 patients) number	No surgery percentage	No surgery median (25th - 75th percentile)	Scheduled surgery (207 patients) number	Scheduled surgery percentage	Scheduled surgery median (25th - 75th percentile)	Unscheduled surgery (364 patients) number	Unscheduled surgery percentage	Unscheduled surgery median (25th - 75th percentile)	Test results: p-value Kruskal-Wallis test	Test results: p-value X^2-test / Fisher's exact test
Age (continuous)				64 (51 - 73.75)			67 (55 - 76)			64 (51 - 75)	0.01578	
Age (categorical: 75 years or older)		243	22.7%		65	31.4%		97	26.6%			0.0181
Gender (male)		631	59.0%		128	61.8%		211	58.0%			0.6566
ECOG performance status												3.4E-08
	Grade 0 (full functional)	347	32.4%		70	33.8%		156	42.9%			
	Grade 1 (symptomatic)	236	22.1%		82	39.6%		86	23.6%			
	Grade 2 (functional but not able to work)	168	15.7%		25	12.1%		47	12.9%			
	Grade 3 (limited functionality)	152	14.2%		13	6.3%		39	10.7%			
	Grade 4 (bedridden)	62	5.8%		8	3.9%		12	3.3%			
	Unknown	105	9.8%		9	4.4%		24	6.6%			
Nursing home resident		62	5.8%		6	2.9%		10	2.7%			0.0251
Moderate to severe comorbidities (number)												3.5E-06
	0	516	48.2%		68	32.9%		206	56.6%			
	1	436	40.7%		105	50.7%		125	34.3%			
	≥ 2	118	11.0%		34	16.4%		33	9.1%			
Type comorbidity												
	Solid tumor	145	13.6%		93	44.9%		66	18.1%			< 2.2 E-16
	Heart failure (NYHA III or IV)	119	11.1%		29	14.0%		44	12.1%			0.47990
	COPD (Gold III or IV or equivalent)	134	12.5%		25	12.1%		31	8.5%			0.11550
	Neurological (excluding dementia)	81	7.6%		9	4.3%		13	3.6%			0.01179
	Hematological malignancy	83	7.8%		4	1.9%		5	1.4%			0.0005
	Liver cirrhosis (Child Pugh B or C)	57	5.3%		6	2.9%		19	5.2%			0.3322
	Chronic renal failure requiring dialysis	36	3.4%		8	3.9%		10	2.7%			0.7518
	Dementia (moderate or severe)	26	2.4%		4	1.9%		9	2.5%			0.9465
	AIDS	14	1.3%		0	0.0%		0	0.0%			< 2.2 E-16
Abuse												
	Alcohol	143	13.4%		10	4.8%		28	7.7%			0.0001
	Active smoking	195	18.2%		35	16.9%		60	16.5%			0.7187
Main admission reason(s)												
	Respiratory failure	336	31.4%		14	6.8%		40	11.0%			< 2.2 E-16
	Sepsis / severe sepsis / septic shock	256	23.9%		8	3.9%		59	16.2%			4.3E-11
	Heart failure / cardiogenic shock	148	13.8%		29	14.0%		102	28.0%			1.8E-09
	Neurologic pathology / Stroke / ICB	134	12.5%		3	1.4%		45	12.4%			0.0005
	Gastro-intestinal pathology / liver failure	93	8.7%		9	4.3%		67	18.4%			1.0E-08
	Metabolic / renal	128	12.0%		4	1.9%		12	3.3%			0.0005
	Multiple trauma	64	6.0%		0	0.0%		32	8.8%			< 2.2 E-16
	Head trauma	37	3.5%		0	0.0%		21	5.8%			< 2.2 E-16
Surgery within 48 hrs		0	0.0%		196	94.7%		364	100.0%			< 2.2 E-16
Do-not-resuscitate order before admission												0.0005
	Full code	937	87.6%		199	96.1%		327	89.8%			
	Unknown	47	4.4%		8	3.9%		20	5.5%			
	No CPR	51	4.8%		0	0.0%		10	2.7%			
	Withholding of therapy	35	3.3%		0	0.0%		7	1.9%			
Severity of illness < 24 hrs after admission												
	Invasive mechanical ventilation	409	38.2%		129	62.3%		246	67.6%			< 2.2 E-16
	Vasopressor need	305	28.5%		101	48.8%		177	48.6%			4.2E-15
	Dialysis	42	3.9%		3	1.4%		10	2.7%			0.1454
Written withholding / withdrawing order < 24 h		60	5.6%		1	0.5%		6	1.6%			0.0005
Characteristics during ICU stay (non missing values)[a]		n = 970			n = 199			n = 342				
	Invasive mechanical ventilation	485	50.0%		137	68.8%		251	73.4%			1.5E-15
	Duration of invasive ventilation (days)			0.5 (0 - 3)			1 (0 - 1)			1 (0 - 3)	0.00003336	
	Vasopressor need	399	41.1%		115	57.8%		202	59.1%			5.7E-10
	Duration of vasopressors (days)			0 (0 - 2)			1 (0 - 2)			1 (0 - 2)	0.000006911	
	Dialysis	85	8.8%		9	4.5%		29	8.5%			0.1328
	Duration of dialysis (days)			0 (0 - 0)			0 (0 - 0)			0 (0 - 0)	0.1582	

[a]Percentages are related to the number of non-missing values

A.2.2 Comparison of the number of PECs

App Table 10: Differences in number of PECs between age subgroups.

	< 75 year old (1236 patients)		≥ 75 year old (405 patients)		p-value X²-test / Fisher's exact test	Estimated difference in risk (older - younger) of concordant PECs [95 % CI]
PECs	Number	Percentage	Number	Percentage	0.0038	
0 or 1 PEC	1131	91.5%	350	86.4%		
≥ 2 PEC	105	8.5%	55	13.6%		5.1 % [1.2 % , 8.9 %]

App Table 11: Differences in number of PECs between cancer type subgroups.

	No cancer (1254 patients)		Solid tumor (299 patients)		Hematological cancer (88 patients)		p-value X²-test / Fisher's exact test
PECs	Number	Percentage	Number	Percentage	Number	Percentage	0.2206
0 or 1 PEC	1140	90.9%	265	88.6%	76	86.4%	
≥ 2 PEC	114	9.1%	34	11.4%	12	13.6%	

App Table 12: Pairwise comparisons in number of PECs between cancer type subgroups.

Group 1	Group 2	p-value X²-test / Fisher's exact test	Estimated difference in risk (group 1 - group 2) of concordant PECs [95 % CI]
No cancer	Solid tumor	0.2726	-2.3 % [-6.4 %, 1.9 %]
No cancer	Hematological cancer	0.2209	-4.5 % [-12.5 %, 3.4 %]
Solid tumor	Hematological cancer	0.6967	-2.2 % [-11.0 %, 6.5 %]

App Table 13: Differences in number of PECs between cancer status subgroups.

	No cancer (1254 patients)		Active cancer (117 patients)		Not active cancer (270 patients)		p-value X²-test / Fisher's exact test
PECs	Number	Percentage	Number	Percentage	Number	Percentage	0.0002
0 or 1 PEC	1140	90.9%	93	79.5%	248	91.9%	
≥ 2 PEC	114	9.1%	24	20.5%	22	8.1%	

App Table 14: Pairwise comparisons in number of PECs between cancer status subgroups.

Group 1	Group 2	p-value X²-test / Fisher's exact test	Estimated difference in risk (group 1 - group 2) of concordant PECs [95 % CI]
No cancer	Active cancer	0.0002	-11.4 % [-19.4 %, -3.5 %]
No cancer	Not active cancer	0.7075	1.0 % [-2.9 %, 4.8 %]
Active cancer	Not active cancer	0.0010	12.4 % [3.7 %, 21.0 %]

App Table 15: Differences in number of PECs between surgery subgroups.

	No surgery (1070 patients)		Scheduled surgery (207 patients)		Unscheduled surgery (364 patients)		p-value X²-test / Fisher's exact test
PECs	Number	Percentage	Number	Percentage	Number	Percentage	0.0376
0 or 1 PEC	957	89.4%	197	95.2%	327	89.8%	
≥ 2 PEC	113	10.6%	10	4.8%	37	10.2%	

App Table 16: Pairwise comparisons in number of PECs between surgery subgroups.

Group 1	Group 2	p-value X²-test / Fisher's	Estimated difference in risk (group 1 - group 2) of concordant PECs [95 % CI]
No surgery	Scheduled surgery	0.0151	5.8 % [2.0 %, 9.5%]
No surgery	Unscheduled surgery	0.9092	0.4 % [-3.4 %, 4.2 %]
Scheduled surgery	Unscheduled surgery	0.0384	-5.4 % [-10.0, -0.7]

A.3 Risks

A.3.1 Risk of death within 28 days

App Table 17: Comparison of age subgroups within the group of patients with and without concordant PECs – number of patients who died within 28 days.

	< 75 year old (1236 patients)		≥ 75 year old (405 patients)		p-value Fisher's exact test
	number	percentage (%)	number	percentage (%)	
**< 2 PECs**	1131	100%	350	100%	
alive after 28 days	923	81.6%	258	73.7%	0.0009
dead after 28 days	168	14.9%	79	22.6%	
lost to follow up	40	3.5%	13	3.7%	
**≥ 2 PECs**	105	100%	55	100%	
alive after 28 days	41	39.0%	22	40.0%	1
dead after 28 days	63	60.0%	33	60.0%	
lost to follow up	1	1.0%	0	0.0%	

* category: "lost to follow up" not used for test

App Table 18: Comparison of cancer type subgroups within the group of patients with and without concordant PECs – number of patients who died within 28 days.

	No cancer (1254 patients)		Solid tumor (299 patients)		Hematological cancer (88 patients)		p-value Fisher's exact test
	number	percentage (%)	number	percentage (%)	number	percentage (%)	
**< 2 PECs**	1140	100%	265	100%	76	100%	
alive after 28 days	922	80.9%	211	79.6%	48	63.2%	0.0020
dead after 28 days	176	15.4%	46	17.4%	25	32.9%	
lost to follow up	42	3.7%	8	3.0%	3	3.9%	
**≥ 2 PECs**	114	100%	34	100%	12	100%	
alive after 28 days	44	38.6%	16	47.1%	3	25.0%	0.4478
dead after 28 days	69	60.5%	18	52.9%	9	75.0%	
lost to follow up	1	0.9%	0	0.0%	0	0.0%	

* category: "lost to follow up" not used for test

App Table 19: Comparison of cancer status subgroups within the group of patients with and without concordant PECs – number of patients who died within 28 days.

	No cancer (1254 patients)		Active cancer (117 patients)		Not active cancer (270 patients)		p-value Fisher's exact test
	number	percentage (%)	number	percentage (%)	number	percentage (%)	
**< 2 PECs**	1140	100%	93	100%	248	100%	
alive after 28 days	922	80.9%	62	66.7%	197	79.4%	0.0320
dead after 28 days	176	15.4%	22	23.7%	49	19.8%	
lost to follow up	42	3.7%	9	9.7%	2	0.8%	
**≥ 2 PECs**	114	100%	24	100%	22	100%	
alive after 28 days	44	38.6%	9	37.5%	10	45.5%	0.8631
dead after 28 days	69	60.5%	15	62.5%	12	54.5%	
lost to follow up	1	0.9%	0	0.0%	0	0.0%	

* category: "lost to follow up" not used for test

App Table 20: Comparison of surgery subgroups within the group of patients with and without concordant PECs – number of patients who died within 28 days.

	No surgery (1070 patients)		Scheduled surgery (207 patients)		Unscheduled surgery (364 patients)		p-value Fisher's exact test
	number	percentage (%)	number	percentage (%)	number	percentage (%)	
**< 2 PECs**	957	100%	197	100%	327	100%	
alive after 28 days	710	74.2%	188	95.4%	283	86.5%	0.0005
dead after 28 days	200	20.9%	8	4.1%	39	11.9%	
lost to follow up	47	4.9%	1	0.5%	5	1.5%	
**≥ 2 PECs**	113	100%	10	100%	37	100%	
alive after 28 days	39	34.5%	8	80.0%	16	43.2%	0.0210
dead after 28 days	73	64.6%	2	20.0%	21	56.8%	
lost to follow up	1	0.9%	0	0.0%	0	0.0%	

* category: "lost to follow up" not used for test

A.3.2 Risk of death within 1 year

App Table 21: Comparison of age subgroups within the group of patients with and without concordant PECs – number of patients who died within 1 year.

	< 75 year old (1236 patients)		≥ 75 year old (405 patients)		p-value Fisher's exact
	number	percentage (%)	number	percentage (%)	
**< 2 PECs**	1131	100%	350	100%	
alive after 1 year	656	58.0%	179	51.1%	
dead after 1 year	285	25.2%	126	36.0%	0.0005
lost to follow up	190	16.8%	45	12.9%	
**≥ 2 PECs**	105	100%	55	100%	
alive after 1 year	17	16.2%	9	16.4%	
dead after 1 year	84	80.0%	44	80.0%	1
lost to follow up	4	3.8%	2	3.6%	

* category: "lost to follow up" not used for test

App Table 22: Comparison of cancer type subgroups within the group of patients with and without concordant PECs – number of patients who died within 1 year.

	No cancer (1254 patients)		Solid tumor (299 patients)		Hematological cancer (88 patients)		p-value Fisher's exact test
	number	percentage (%)	number	percentage (%)	number	percentage (%)	
**< 2 PECs**	1140	100%	265	100%	76	100%	
alive after 1 year	675	59.2%	131	49.4%	29	38.2%	0.0005
dead after 1 year	273	23.9%	99	37.4%	39	51.3%	
lost to follow up	192	16.8%	35	13.2%	8	10.5%	
**≥ 2 PECs**	114	100%	34	100%	12	100%	
alive after 1 year	20	17.5%	4	11.8%	2	16.7%	0.7811
dead after 1 year	89	78.1%	29	85.3%	10	83.3%	
lost to follow up	5	4.4%	1	2.9%	0	0.0%	

* category: "lost to follow up" not used for test

App Table 23: Comparison of cancer status subgroups within the group of patients with and without concordant PECs – number of patients who died within 1 year.

	No cancer (1254 patients)		Active cancer (82 patients)		Not active cancer (222 patients)		p-value Fisher's exact test
	number	percentage (%)	number	percentage (%)	number	percentage (%)	
**< 2 PECs**	1140	100%	9	100%	248	100%	
alive after 1 year	675	59.2%	31	33.3%	129	52.0%	0.0005
dead after 1 year	273	23.9%	43	46.2%	95	38.0%	
lost to follow up	192	16.8%	19	20.4%	24	9.7%	
**≥ 2 PECs**	114	100%	24	100%	22	100%	
alive after 1 year	20	17.5%	2	8.3%	4	18.2%	0.5262
dead after 1 year	89	78.1%	22	91.7%	17	77.3%	
lost to follow up	5	4.4%	0	0.0%	1	4.5%	

* category: "lost to follow up" not used for test

App Table 24: Comparison of surgery subgroups within the group of patients with and without concordant PECs – number of patients who died within 1 year.

	No surgery (1070 patients)		Scheduled surgery (207 patients)		Unscheduled surgery (364 patients)		p-value Fisher's exact test
	number	percentage (%)	number	percentage (%)	number	percentage (%)	
**< 2 PECs**	957	100%	197	100%	327	100%	
alive after 1 year	463	48.4%	154	78.2%	218	66.7%	0.0005
dead after 1 year	312	32.6%	29	14.7%	70	21.4%	
lost to follow up	182	19.0%	14	7.1%	39	11.9%	
**≥ 2 PECs**	113	100%	10	100%	37	100%	
alive after 1 year	13	11.5%	4	40.0%	9	24.3%	0.0310
dead after 1 year	94	83.2%	6	60.0%	28	75.7%	
lost to follow up	6	5.3%	0	0.0%	0	0.0%	

* category: "lost to follow up" not used for test

A.3.3 Risk of combined endpoint

App Table 25: Comparison of age subgroups within the group of patients with and without concordant PECs – number of patients who reached CEP.

	< 75 year old (1236 patients)		≥ 75 year old (405 patients)		p-value Fisher's exact test
	number	percentage (%)	number	percentage (%)	
**< 2 PECs**	1131	100%	350	100%	
**Lost to follow up**	273	24.1%	58	16.6%	
**Not reaching combined endpoint**	405	35.8%	112	32.0%	0.0096
**Reaching combined endpoint**	453	40.1%	180	51.4%	
1) dead	*285*	*25.2%*	*126*	*36.0%*	
2) not at home / bad QOL	*168*	*14.9%*	*54*	*15.4%*	
**≥ 2 PECs**	105	100%	55	100%	
**Lost to follow up**	5	4.8%	3	5.5%	
**Not reaching combined endpoint**	7	6.7%	5	9.1%	0.5455
**Reaching combined endpoint**	93	88.6%	47	85.5%	
1) dead	*84*	*80.0%*	*44*	*80.0%*	
2) not at home / bad QOL	*9*	*8.6%*	*3*	*5.5%*	

App Table 26: Comparison of cancer type subgroups within the group of patients with and without concordant PECs – number of patients who reached CEP.

	No cancer (1254 patients)		Solid tumor (299 patients)		Hematological cancer (88 patients)		p-value Fisher's exact test
	number	percentage (%)	number	percentage (%)	number	percentage (%)	
< 2 PECs	1140	100%	265	100%	76	100%	
Lost to follow up	268	23.5%	49	18.5%	14	18.4%	
Not reaching combined endpoint	419	36.8%	85	32.1%	13	17.1%	0.0005
Reaching combined endpoint	453	39.7%	131	49.4%	49	64.5%	
1) dead	*273*	*23.9%*	*99*	*37.4%*	*39*	*51.3%*	
2) not at home / bad QOL	*180*	*15.8%*	*32*	*12.1%*	*10*	*13.2%*	
≥ 2 PECs	114	100%	34	100%	12	100%	
Lost to follow up	7	6.1%	1	2.9%	0	0.0%	
Not reaching combined endpoint	8	7.0%	3	8.8%	1	8.3%	0.8856
Reaching combined endpoint	99	86.8%	30	88.2%	11	91.7%	
1) dead	*89*	*78.1%*	*29*	*85.3%*	*10*	*83.3%*	
2) not at home / bad QOL	*10*	*8.8%*	*1*	*2.9%*	*1*	*8.3%*	

App Table 27: Comparison of cancer status subgroups within the group of patients with and without concordant PECs – number of patients who reached CEP.

	No cancer (1254 patients)		Active cancer (117 patients)		Not active cancer (270 patients)		p-value Fisher's exact test
	number	percentage (%)	number	percentage (%)	number	percentage (%)	
< 2 PECs	1140	100%	93	100%	248	100%	
Lost to follow up	268	23.5%	23	24.7%	40	16.1%	
Not reaching combined endpoint	419	36.8%	16	17.2%	82	33.1%	0.0005
Reaching combined endpoint	453	39.7%	54	58.1%	126	50.8%	
1) dead	*273*	*23.9%*	*43*	*46.2%*	*95*	*38.3%*	
2) not at home / bad QOL	*180*	*15.8%*	*11*	*11.8%*	*31*	*12.5%*	
≥ 2 PECs	114	100%	24	100%	22	100%	
Lost to follow up	7	6.1%	0	0.0%	1	4.5%	
Not reaching combined endpoint	8	7.0%	1	4.2%	3	13.6%	0.4938
Reaching combined endpoint	99	86.8%	23	95.8%	18	81.8%	
1) dead	*89*	*78.1%*	*22*	*91.7%*	*17*	*77.3%*	
2) not at home / bad QOL	*10*	*8.8%*	*1*	*4.2%*	*1*	*4.5%*	

App Table 28: Comparison of surgery subgroups within the group of patients with and without concordant PECs – number of patients who reached CEP.

	No surgery (1070 patients)		Scheduled surgery (207 patients)		Unscheduled surgery (364 patients)		p-value Fisher's exact test
	number	percentage (%)	number	percentage (%)	number	percentage (%)	
< 2 PECs	957	100%	197	100%	327	100%	
Lost to follow up	243	25.4%	25	12.7%	63	19.3%	
Not reaching combined endpoint	263	27.5%	107	54.3%	147	45.0%	0.0005
Reaching combined endpoint	451	47.1%	65	33.0%	117	35.8%	
1) dead	*312*	*32.6%*	*29*	*14.7%*	*70*	*21.4%*	
2) not at home / bad QOL	*139*	*14.5%*	*36*	*18.3%*	*47*	*14.4%*	
≥ 2 PECs	113	100%	10	100%	37	100%	
Lost to follow up	6	5.3%	1	10.0%	1	2.7%	
Not reaching combined endpoint	6	5.3%	2	20.0%	4	10.8%	0.1164
Reaching combined endpoint	101	89.4%	7	70.0%	32	86.5%	
1) dead	*94*	*83.2%*	*6*	*60.0%*	*28*	*75.7%*	
2) not at home / bad QOL	*7*	*6.2%*	*1*	*10.0%*	*4*	*10.8%*	

A.3.4 Risk of TLD

App Table 29: Comparison of age subgroups within the group of patients with and without concordant PECs – number of patients with TLD.

	< 75 year old (1236 patients)		≥ 75 year old (405 patients)		p-value Fisher's exact test
	number	percentage (%)	number	percentage (%)	
**< 2 PECs**	1131	100%	350	100%	
no TLD	1078	95.3%	311	88.9%	3.8E-05
TLD	53	4.7%	39	11.1%	
**≥ 2 PECs**	105	100%	55	100%	
no TLD	72	68.6%	37	67.3%	0.8604
TLD	33	31.4%	18	32.7%	

App Table 30: Comparison of cancer type subgroups within the group of patients with and without concordant PECs – number of patients with TLD.

	No cancer (1254 patients)		Solid tumor (299 patients)		Hematological cancer (88 patients)		p-value Fisher's exact test
	number	percentage (%)	number	percentage (%)	number	percentage (%)	
**< 2 PECs**	1140	100%	265	100%	76	100%	
no TLD	1069	93.8%	248	93.6%	72	94.7%	0.9790
TLD	71	6.2%	17	6.4%	4	5.3%	
**≥ 2 PECs**	114	100%	34	100%	12	100%	
no TLD	75	65.8%	24	70.6%	10	83.3%	0.4908
TLD	39	34.2%	10	29.4%	2	16.7%	

App Table 31: Comparison of cancer status subgroups within the group of patients with and without concordant PECs – number of patients with TLD.

	No cancer (1254 patients)		Active cancer (117 patients)		Not active cancer (270 patients)		p-value Fisher's exact test
	number	percentage (%)	number	percentage (%)	number	percentage (%)	
**< 2 PECs**	1140	100%	93	100%	248	100%	
no TLD	1069	93.8%	84	90.3%	236	95.2%	0.2704
TLD	71	6.2%	9	9.7%	12	4.8%	
**≥ 2 PECs**	114	100%	24	100%	22	100%	
no TLD	75	65.8%	18	75.0%	16	72.7%	0.6872
TLD	39	34.2%	6	25.0%	6	27.3%	

App Table 32: Comparison of surgery subgroups within the group of patients with and without concordant PECs – number of patients with TLD.

	No surgery (1070 patients)		Scheduled surgery (207 patients)		Unscheduled surgery (364 patients)		p-value Fisher's exact test
	number	percentage (%)	number	percentage (%)	number	percentage (%)	
**< 2 PECs**	957	100%	197	100%	327	100%	
no TLD	878	91.7%	195	99.0%	316	96.6%	0.0005
TLD	79	8.3%	2	1.0%	11	3.4%	
**≥ 2 PECs**	113	100%	10	100%	37	100%	
no TLD	75	66.4%	9	90.0%	25	67.6%	0.3538
TLD	38	33.6%	1	10.0%	12	32.4%	

A.4 Unweighted cumulative incidence curves – Schoenfeld residuals

A.4.1 Summary

All hazard ratios mentioned in sections 7 and 9 were obtained via the (cause-specific) Cox proportional hazard models that were fitted to the data. The proportional hazard assumptions have been checked again by plotting the Schoenfeld residuals in function of the time (see App Figure 1 to App Figure 12 for the unweighted cases and App Figure 13 to App Figure 24 for the weighted cases). For the unweighted model to estimate the cause-specific hazard rate or receiving concordant PECs for the cancer type subgroups and for the weighted models to estimate the cause-specific hazard rate or receiving concordant PECs for the cancer type and cancer status subgroups, the Schoenfeld residuals did vary in function of the time and the proportional hazard assumption didn't hold (p-values were 0.0066, 0.0105 and 0.0156 respectively). In all other cases the proportional hazard assumption did hold (p-values between 0.0502 and 0.9292).

A.4.2 Age subgroups

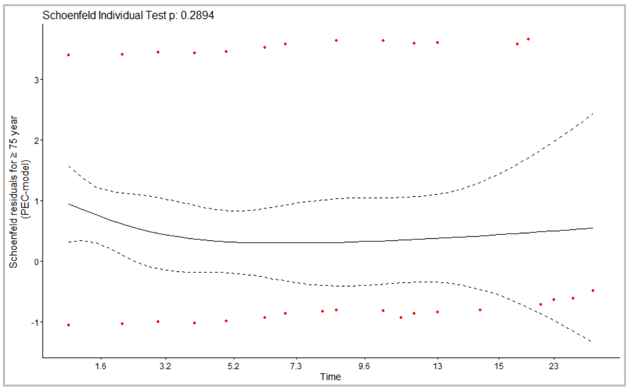

App Figure 1: Schoenfeld residuals for age-coefficient for time from admission until receiving 2nd PEC.

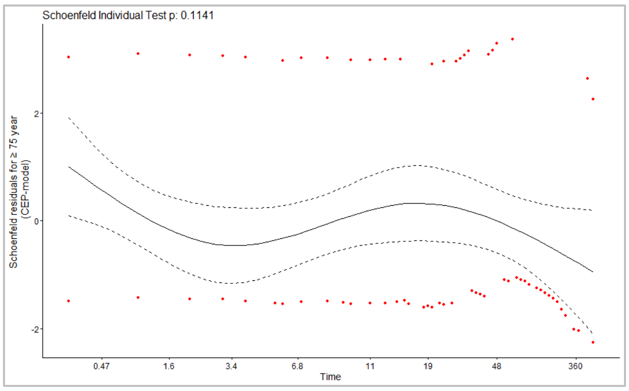

App Figure 2: Schoenfeld residuals for age-coefficient for time between receiving 2nd PEC and death or CEP.

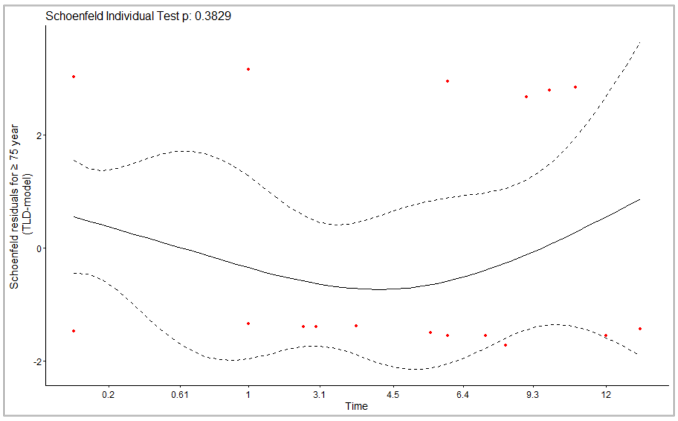

App Figure 3: Schoenfeld residuals for age-coefficient for time between receiving 2nd PEC and TLD.

A.4.3 Cancer type subgroups

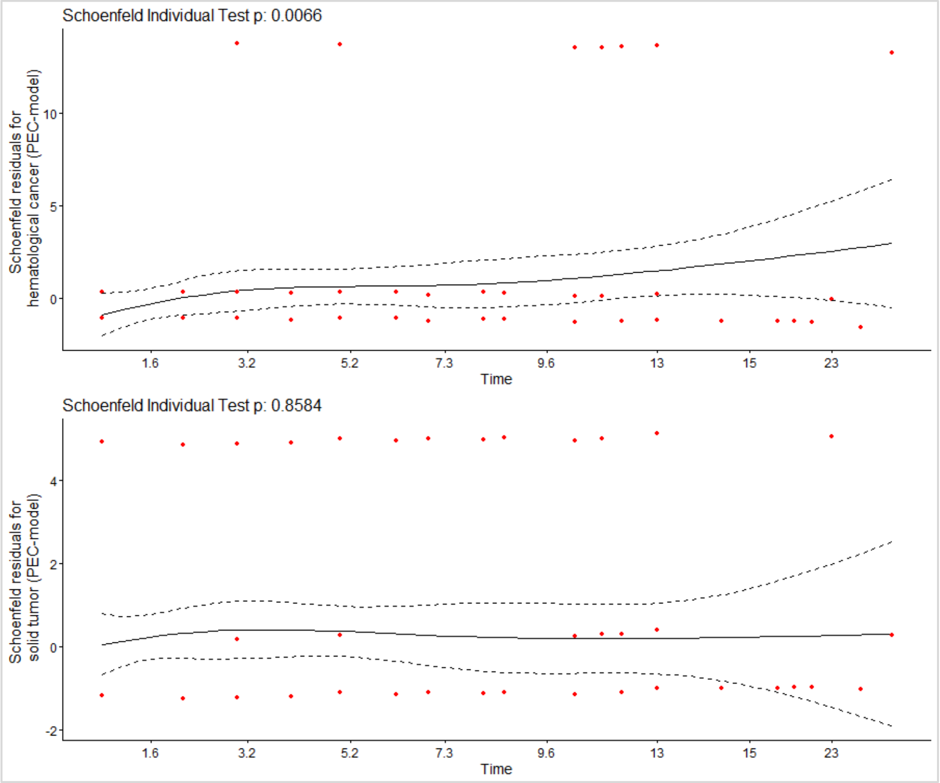

App Figure 4: Schoenfeld residuals for cancer type coefficients for time from admission until receiving 2nd PEC.

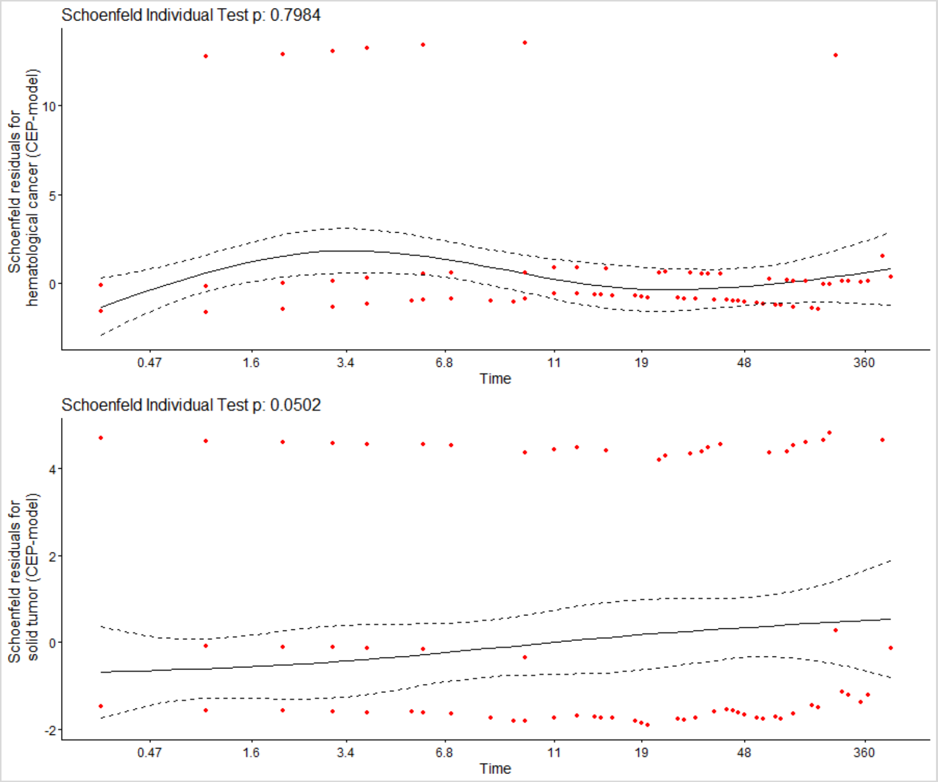

App Figure 5: Schoenfeld residuals for cancer type coefficients for time between receiving 2nd PEC and death or CEP.

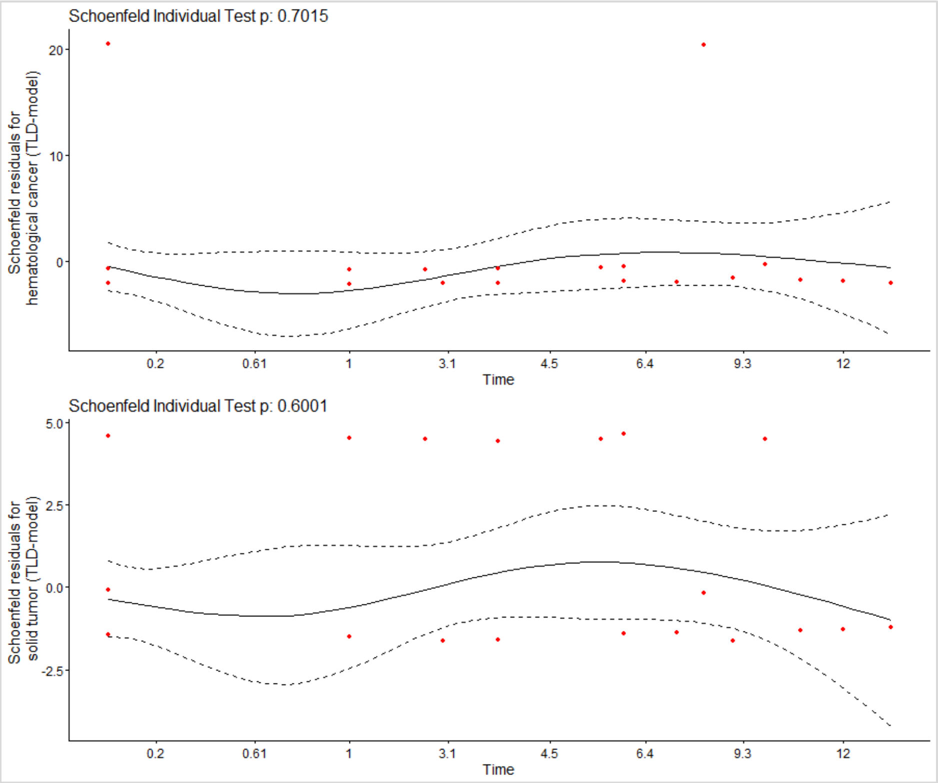

App Figure 6: Schoenfeld residuals for cancer type coefficients for time between receiving 2nd PEC and TLD.

A.4.4 Cancer status subgroups

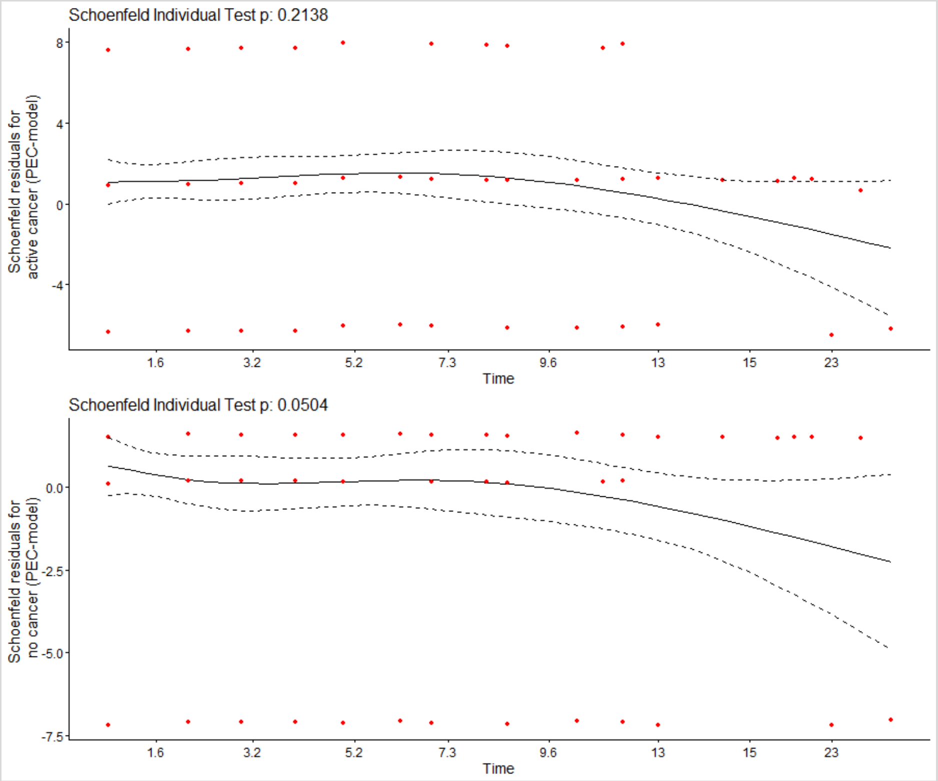

App Figure 7: Schoenfeld residuals for cancer status coefficients for time from admission until receiving 2nd PEC.

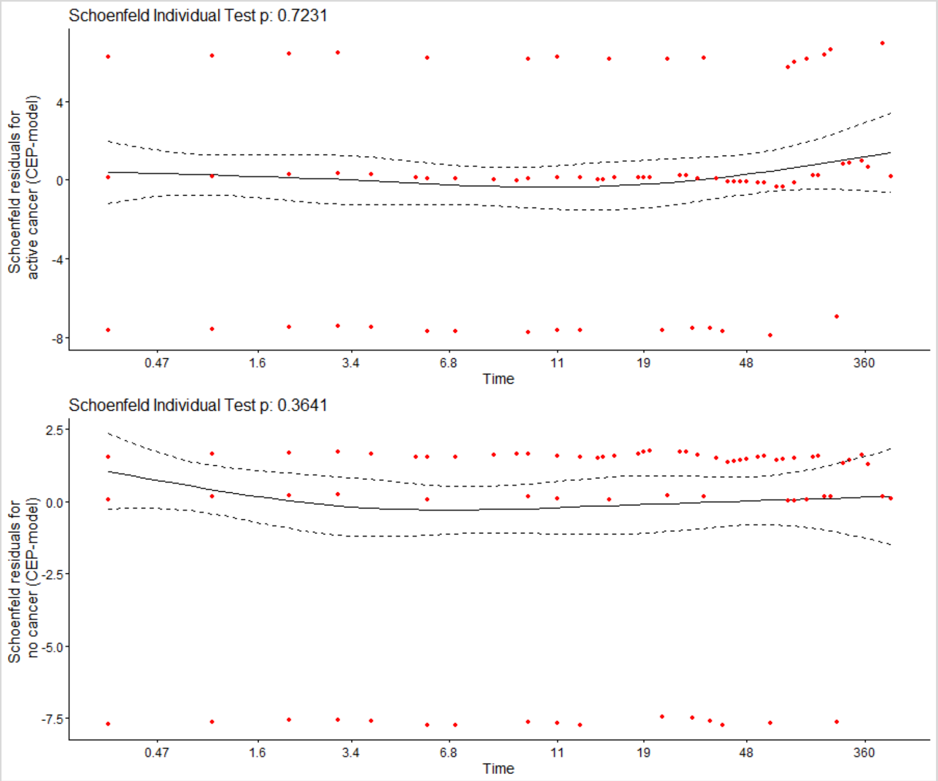

App Figure 8: Schoenfeld residuals for cancer status coefficients for time between receiving 2nd PEC and death or CEP.

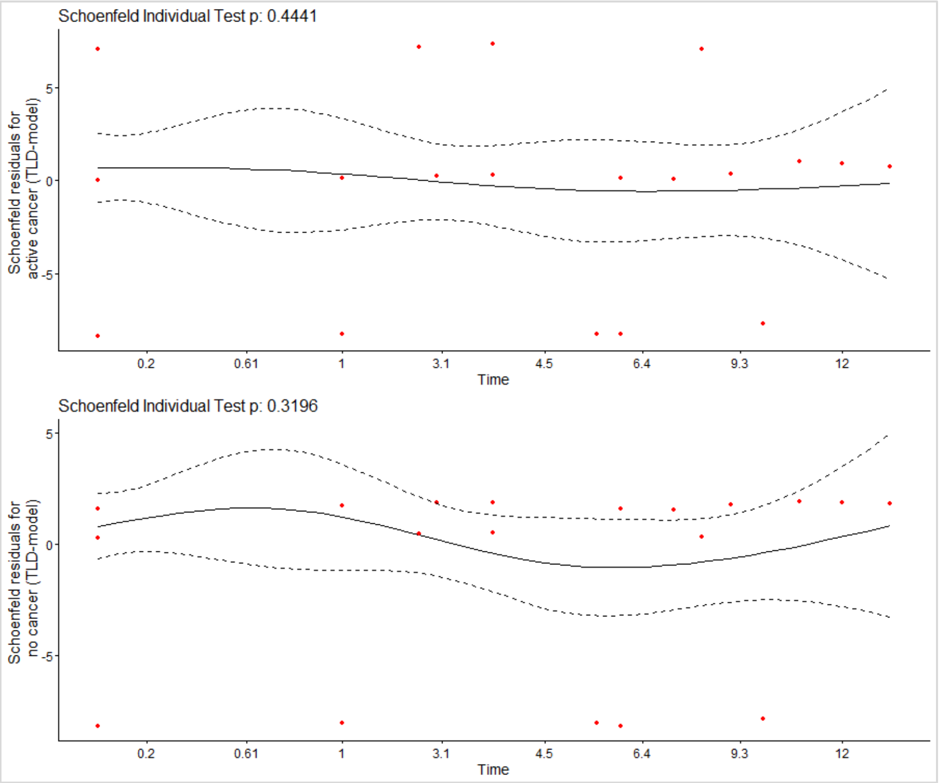

App Figure 9: Schoenfeld residuals for cancer status coefficients for time between receiving 2nd PEC and TLD.

A.4.5 Surgery subgroups

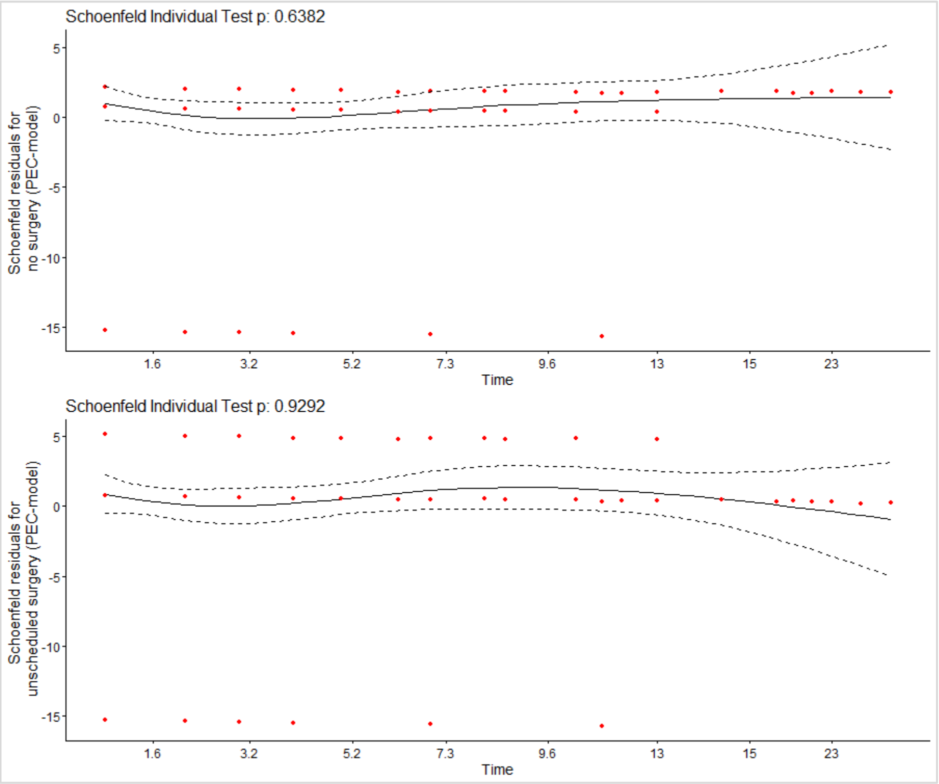

App Figure 10: Schoenfeld residuals for surgery coefficients for time from admission until receiving 2nd PEC.

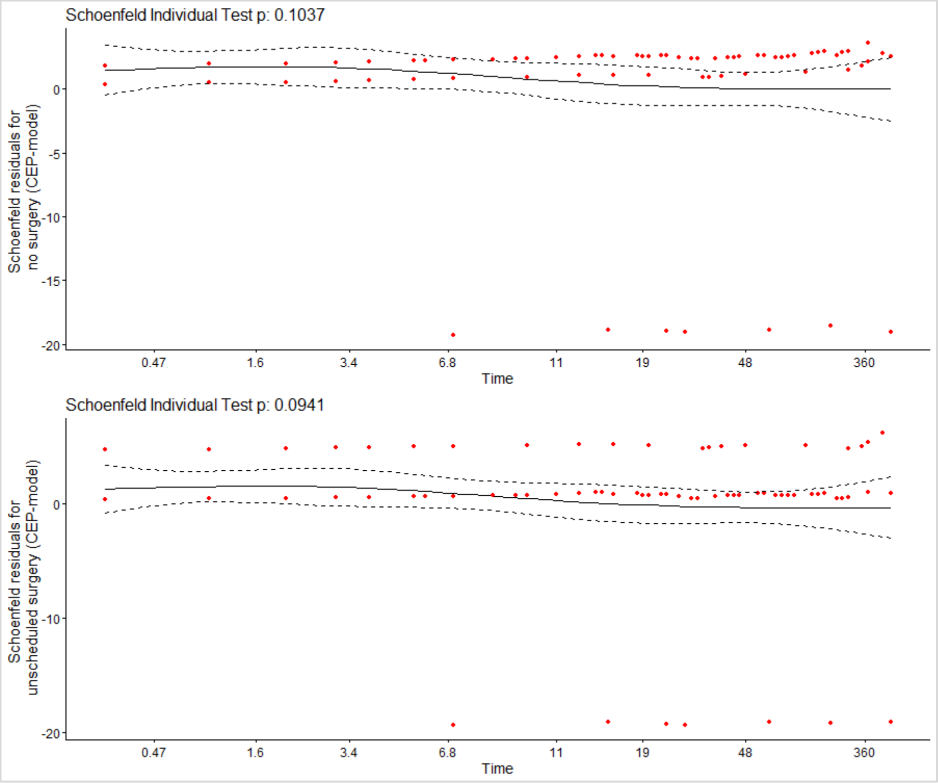

App Figure 11: Schoenfeld residuals for surgery coefficients for time between receiving 2nd PEC and death or CEP.

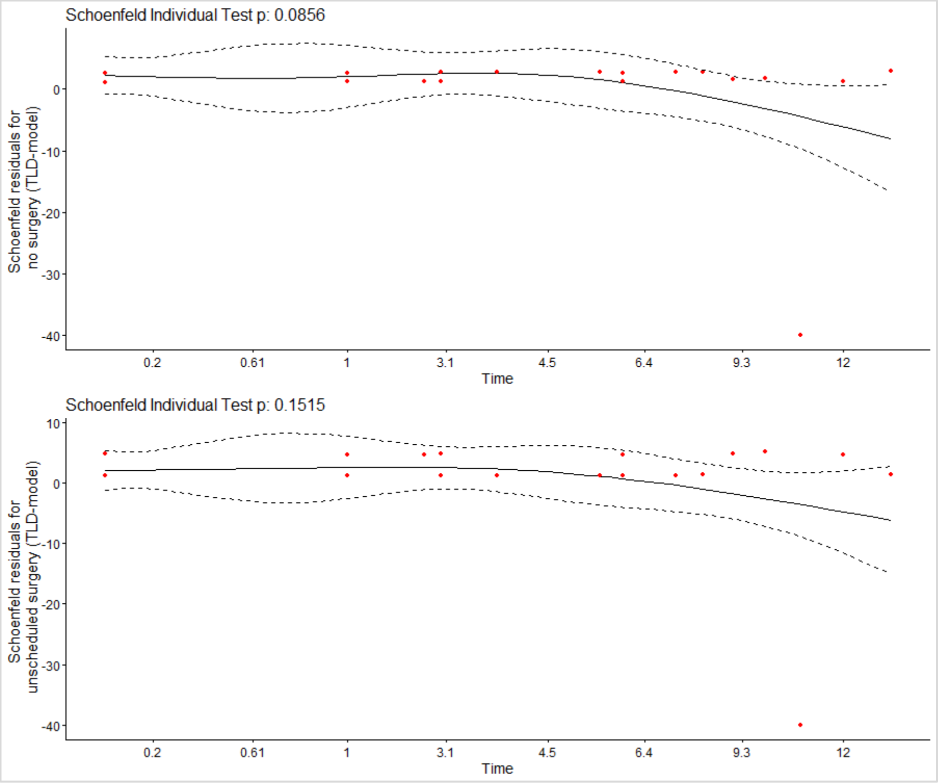

App Figure 12: Schoenfeld residuals for surgery coefficients for time between receiving 2nd PEC and TLD.

A.5 Weighting

A.5.1 Goodness-of-fit tests for multinomial regression models

As was explained in section 8, multinomial logistic regression models have been built to estimate the propensity scores for all patients. The goodness of fit of these models has been assesed by the "Le Cessie-van Houwelingen-Copas-Hosmer" test. The resulting p-values of the different tests have been presented in App Table 33. No evidence for a lack of fit for any of the models has been detected.

App Table 33: p-values Le Cessie-van Houwelingen-Copas-Hosmer goodness-of-fit test.

Subgroup	p-value
Age subgoups	0.264
Cancer type subgoups	
hematological cancer	0.962
solid cancer	0.172
no cancer	0.054
Cancer status subgoups	
active cancer	0.052
not active cancer	0.161
no cancer	0.360
Surgery subgoups	
No surgery	0.059
Scheduled surgery	0.113
Unscheduled surgery	0.345

A.5.2 Range of weights

App Table 34: Minimum, 25th percentile, median, mean, 75th percentile and maximum value of different types of weights.

	Minimum	25th percentile	Median	Mean	75th percentile	Maximum
Weights for age	1.00	1.17	1.36	1.81	1.89	5.03
Weights for cancer type	1.00	1.02	1.08	1.07	1.71	5.76
Weights for cancer status	1.00	1.02	1.09	1.77	1.70	6.26
Weights for surgery type	1.00	1.18	1.48	2.29	2.49	7.98

A.6 Weighted cumulative incidence curves – Schoenfeld residuals

A.6.1 Age subgroups

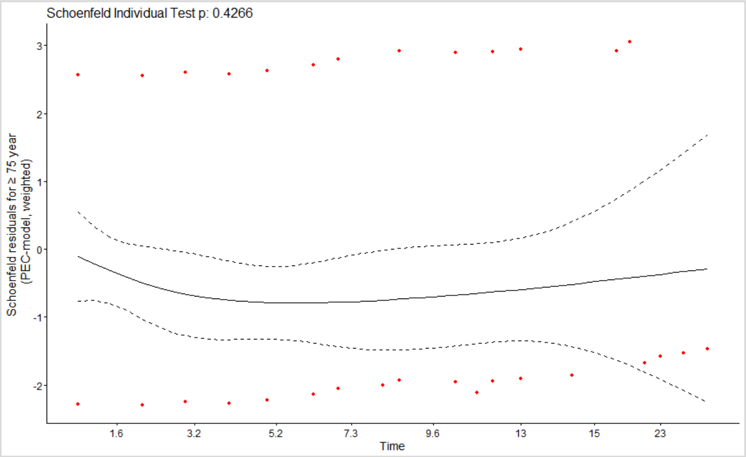

App Figure 13: Schoenfeld residuals for age-coefficient for time from admission until receiving 2nd PEC – weighted.

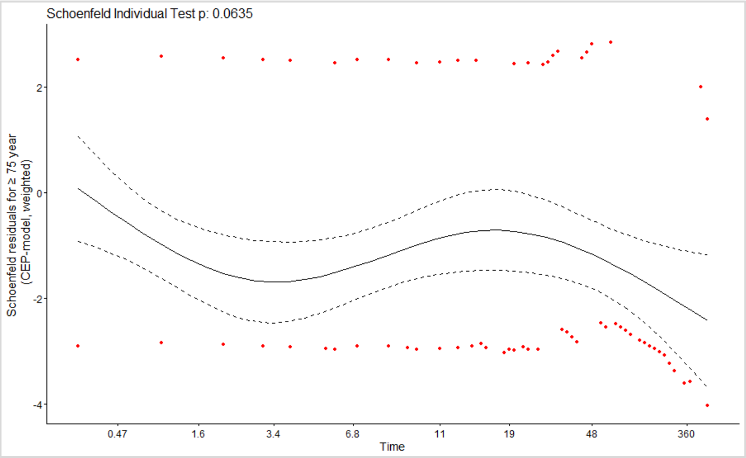

App Figure 14: Schoenfeld residuals for age-coefficient for time between receiving 2nd PEC and death or CEP – weighted.

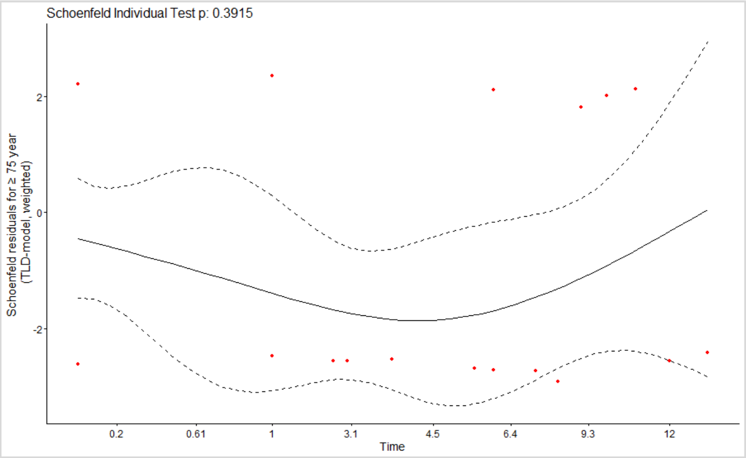

App Figure 15: Schoenfeld residuals for age-coefficient for time between receiving 2nd PEC and TLD – weighted.

A.6.2 Cancer type subgroups

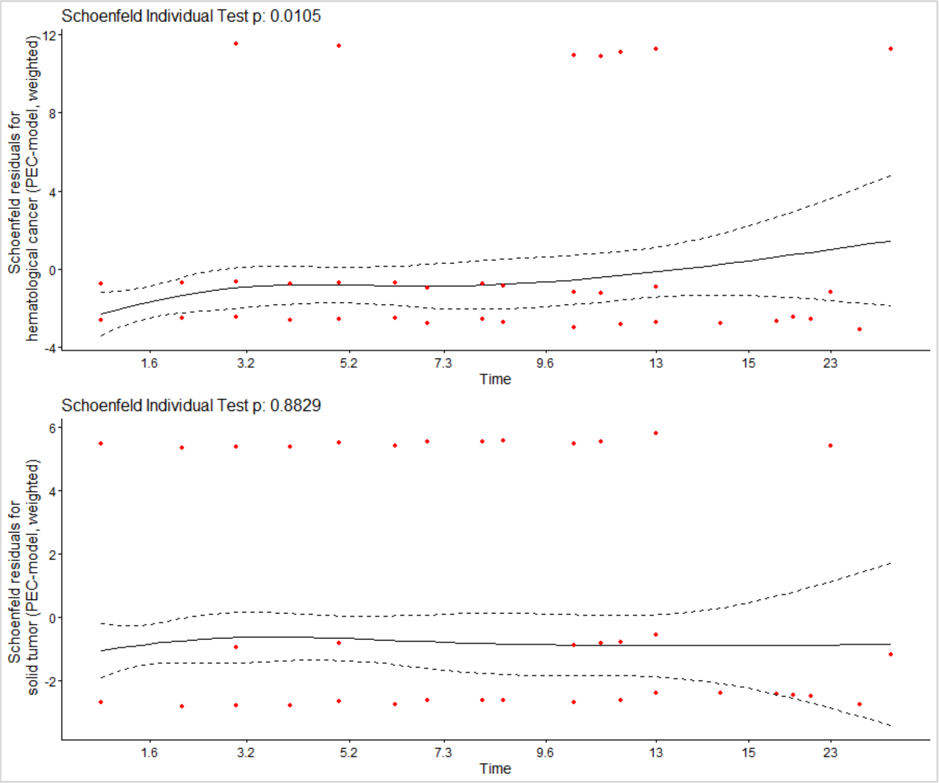

App Figure 16: Schoenfeld residuals for cancer type coefficients for time from admission until receiving 2nd PEC – weighted.

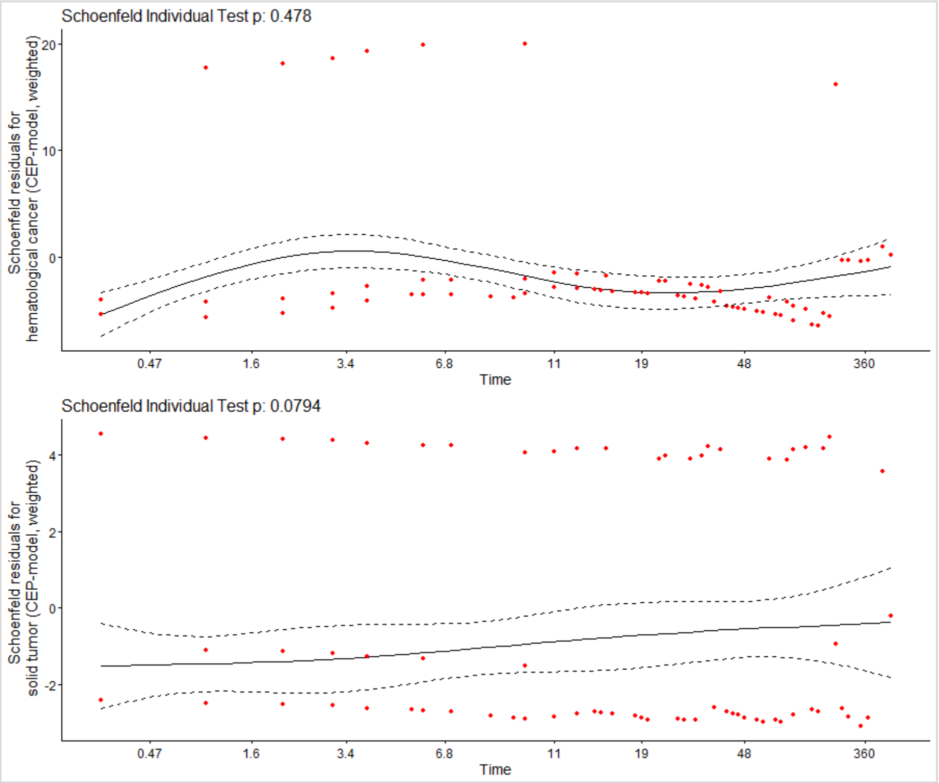

App Figure 17: Schoenfeld residuals for cancer type coefficients for time between receiving 2nd PEC and death or CEP – weighted.

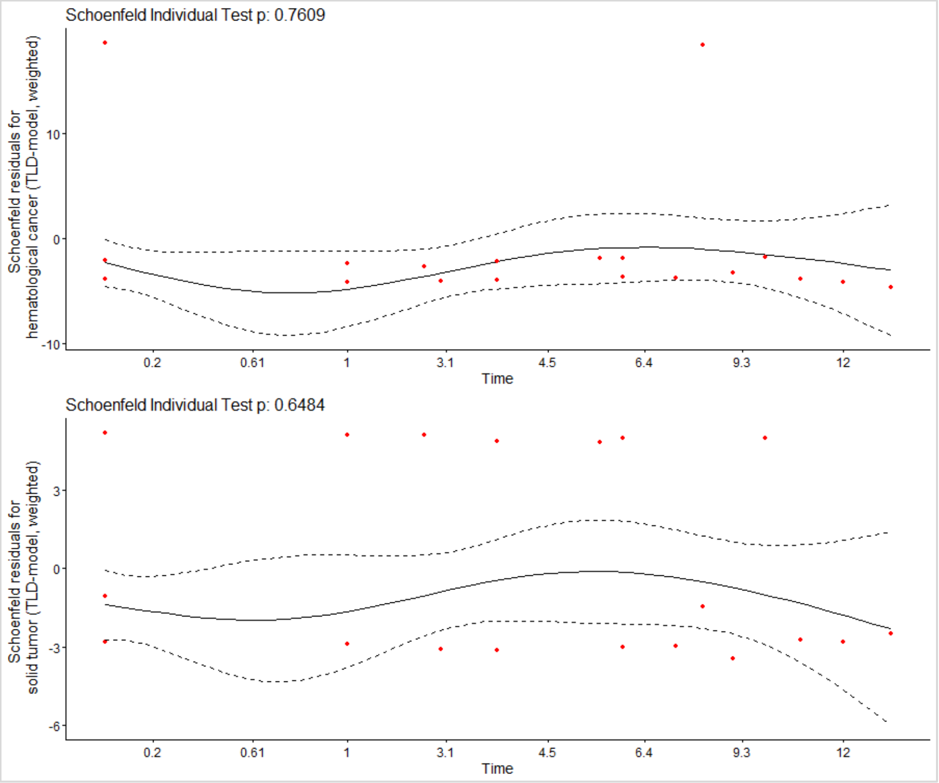

App Figure 18: Schoenfeld residuals for cancer type coefficients for time between receiving 2nd PEC and TLD – weighted.

A.6.3 Cancer status subgroups

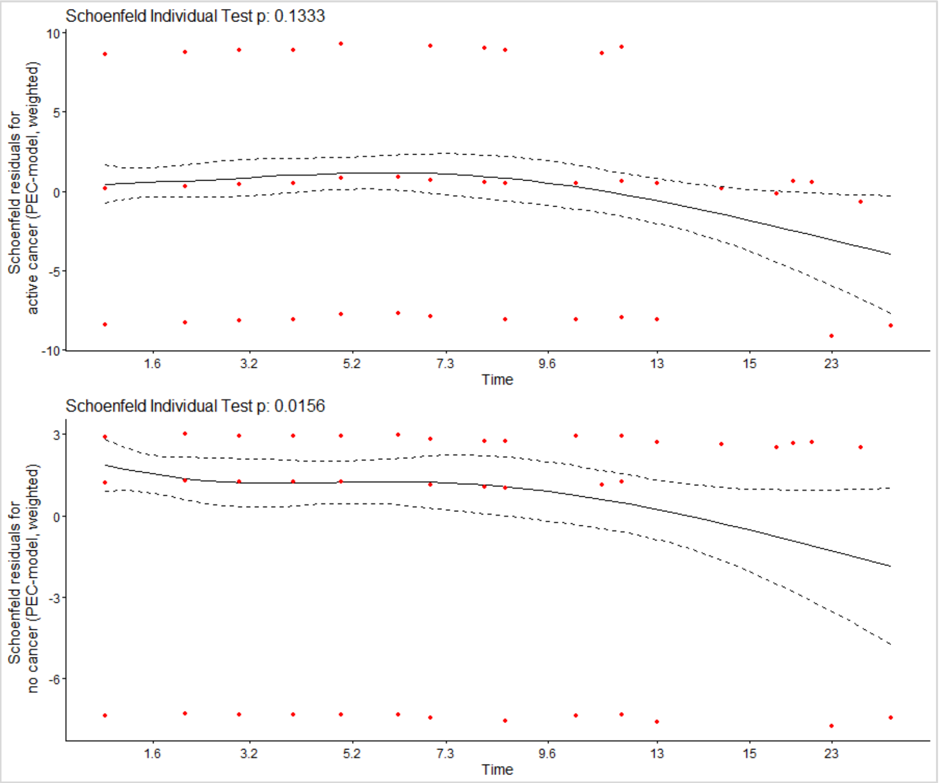

App Figure 19: Schoenfeld residuals for cancer status coefficients for time from admission until receiving 2nd PEC – weighted.

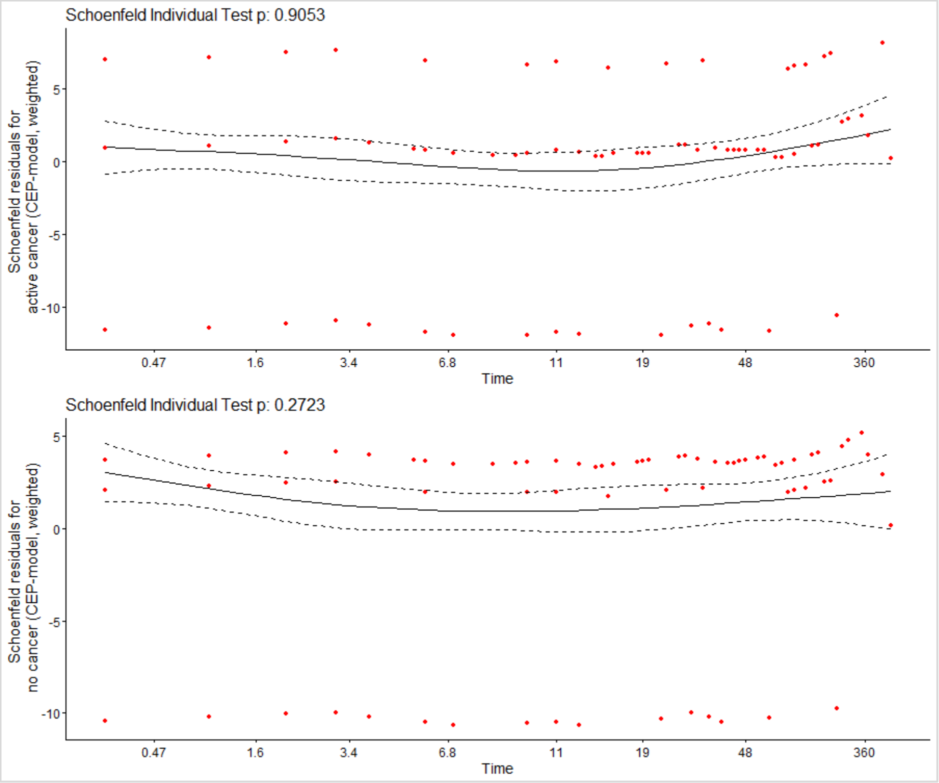

App Figure 20: Schoenfeld residuals for cancer status coefficients for time between receiving 2nd PEC and death or CEP – weighted.

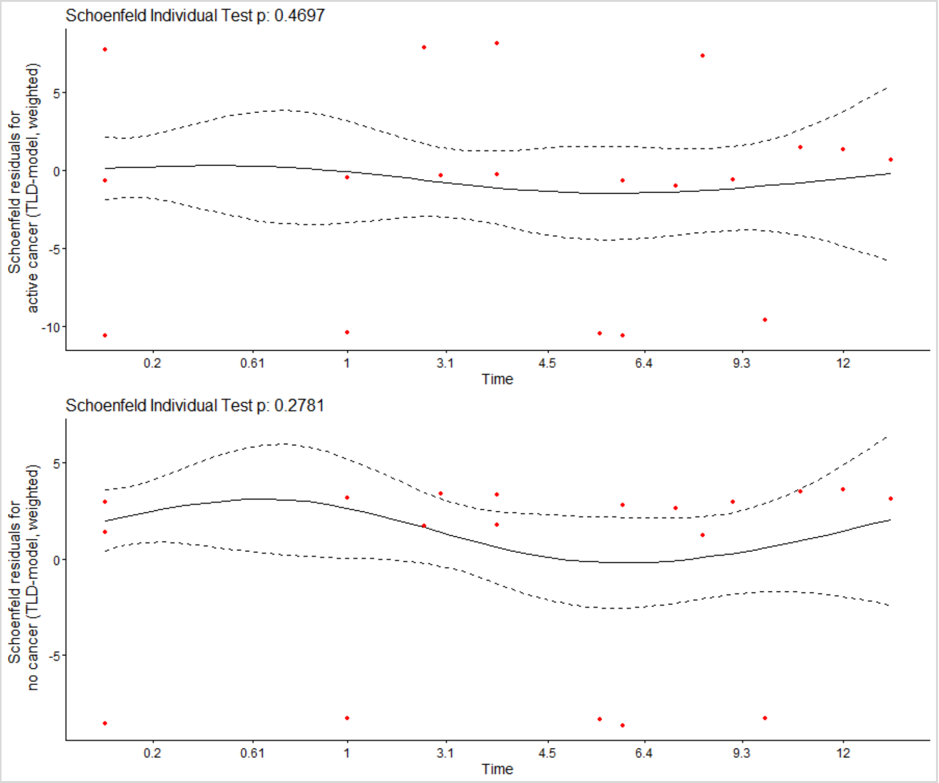

App Figure 21: Schoenfeld residuals for cancer status coefficients for time between receiving 2nd PEC and TLD – weighted.

A.6.4 Surgery subgroups

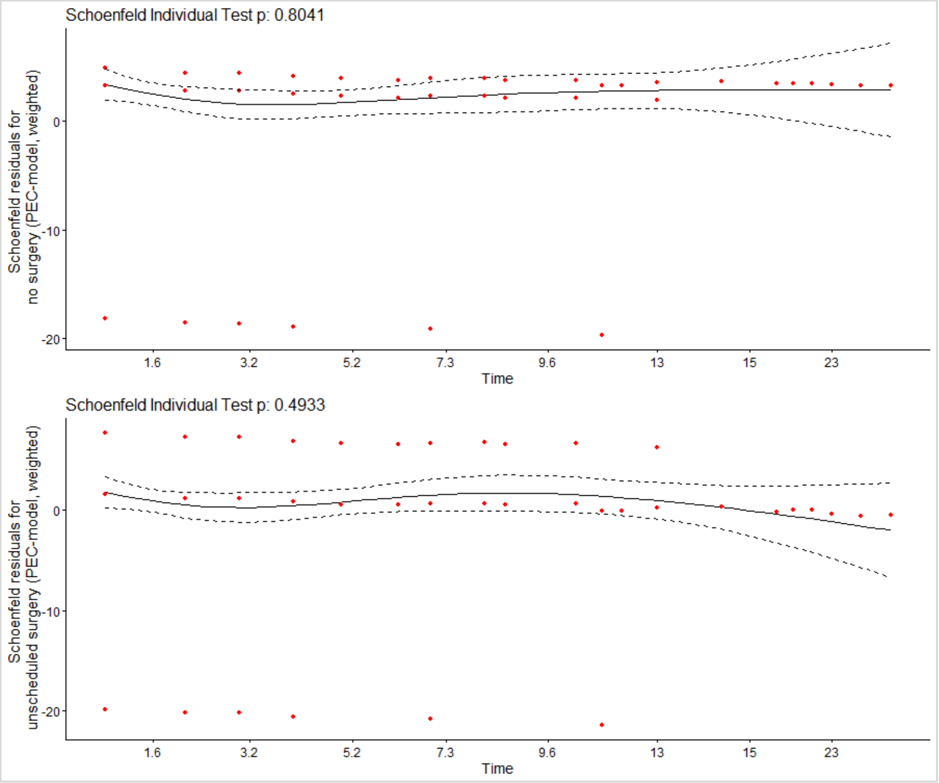

App Figure 22: Schoenfeld residuals for surgery coefficients for time from admission until receiving 2nd PEC – weighted.

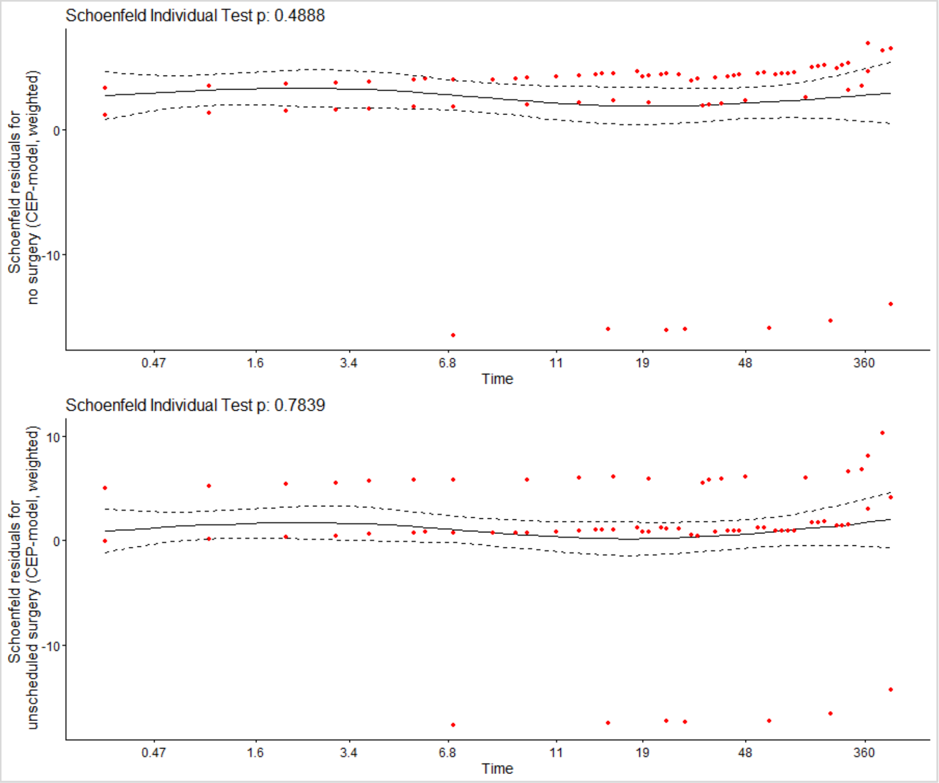

App Figure 23: Schoenfeld residuals for surgery coefficients for time between receiving 2nd PEC and death or CEP – weighted.

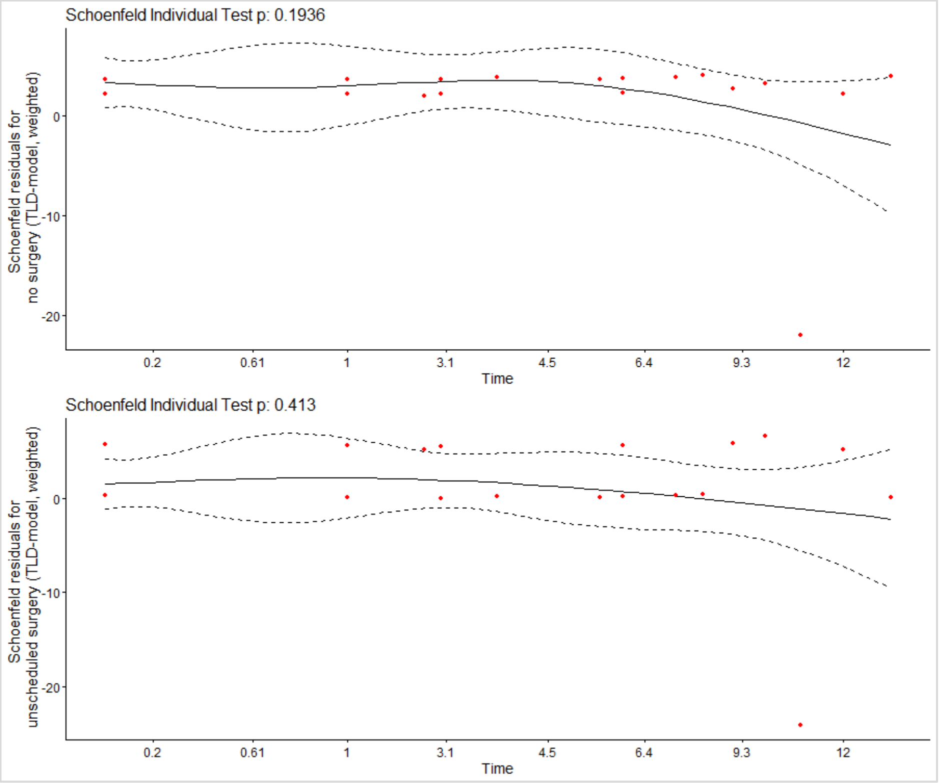

App Figure 24: Schoenfeld residuals for surgery coefficients for time between receiving 2nd PEC and TLD – weighted.
