# Supplementary material for: Outcomes of ICU patients with and without perceptions of excessive care: a comparison between cancer and non-cancer patients
Source: Ann Intensive Care. 2021 Jul 31;11:120. doi: 10.1186/s13613-021-00895-5 (PMC8325749; doi:10.1186/s13613-021-00895-5)
Supplement: Supplementary file 3 — Additional file 3: Fig. S1. Flowchart study unweighted results: number of ICU’s, clinicians, perceptions and patients. PEC: Perceptions of Excessive Care. Combined endpoint: death, poor quality of life or not being at home. [file 13613_2021_895_MOESM3_ESM.docx]

Figure S 1 Flowchart study unweighted results: number of ICU’s, clinicians, perceptions and patients

15 countries

1 country did not participate

1 country unprepared

Phase I

Phase II

13 countries
68 ICUs

2993 clinicians
(63% of 4747)

Excluding Average (-) ethical climate

2690 clinicians

Phase III

 1641 patients admitted during the study period
25025 perceptions by 2293 clinicians (85.2%)

PEC in 334 patients (20.3%) by 728 clinicians (27%)
160 patients with concordant PECs (9.8%)

270 patients with controlled cancer

0 PEC: 216 (80.0%)
1 PEC: 32 (11.9%)
≥ 2 PEC: 22 (8.1%)

117 patients with uncontrolled cancer

0 PEC: 79 (67.5%)
1 PEC: 14 (12.0%)
≥ 2 PEC: 24 (20.5%)

1254 patients without cancer

0 PEC: 1012 (80.7%)
1 PEC: 128 (10.2%)
≥ 2 PEC: 114 (9.1%)

Phase IV

Combined endpoint

< 2 PEC:
58.1%

Combined endpoint

≥ 2 PEC:
95.8%

Combined endpoint

< 2 PEC:
50.8%

Combined endpoint

≥ 2 PEC:
81.8%

Combined endpoint

< 2 PEC:
39.7%

Combined endpoint

≥ 2 PEC:
86.8%

23 patients
lost to follow up

< 2 PEC: 70
≥ 2 PEC: 24

41 patients
lost to follow up

< 2 PEC: 208
≥ 2 PEC: 21

275 patients
lost to follow up

< 2 PEC: 872
≥ 2 PEC: 107
