# Supplementary material for: Outcomes of ICU patients with and without perceptions of excessive care: a comparison between cancer and non-cancer patients
Source: Ann Intensive Care. 2021 Jul 31;11:120. doi: 10.1186/s13613-021-00895-5 (PMC8325749; doi:10.1186/s13613-021-00895-5)

Figure S2

**a** Time from ICU admission until at least 2 PECs during ICU stay (unweighted). **b** Time from ICU admission until death (unweighted). c Time from ICU admission until TLD during ICU stay (unweighted). *TLD* treatment limitation decision, *PEC* perception of excessive care

**a**


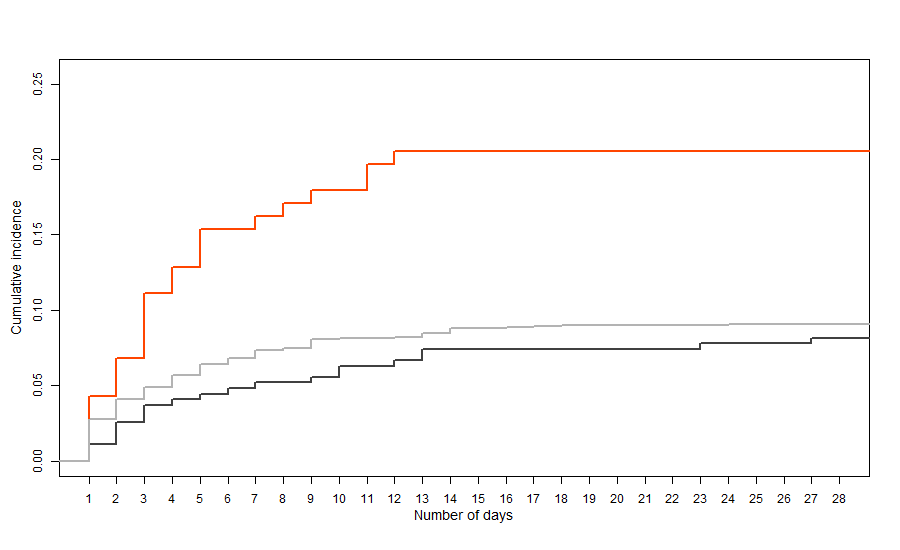


**b**
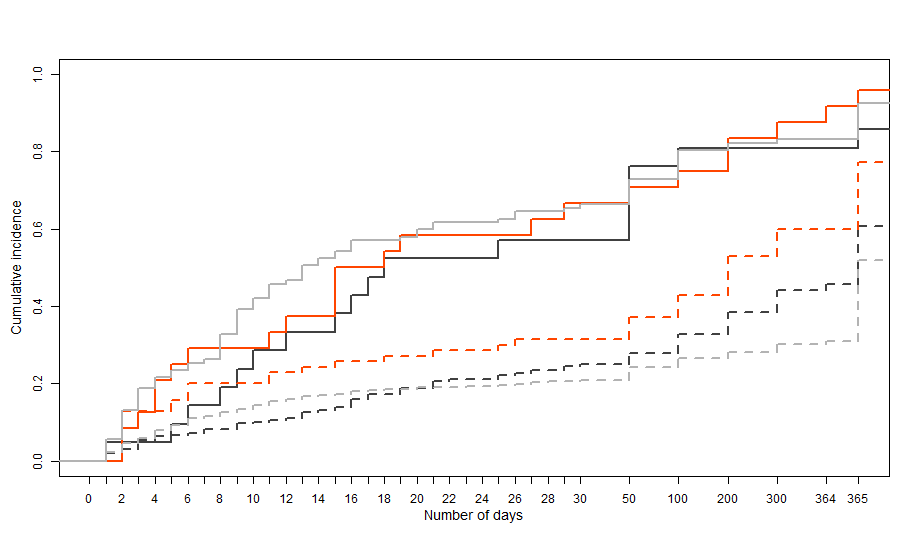


**c**
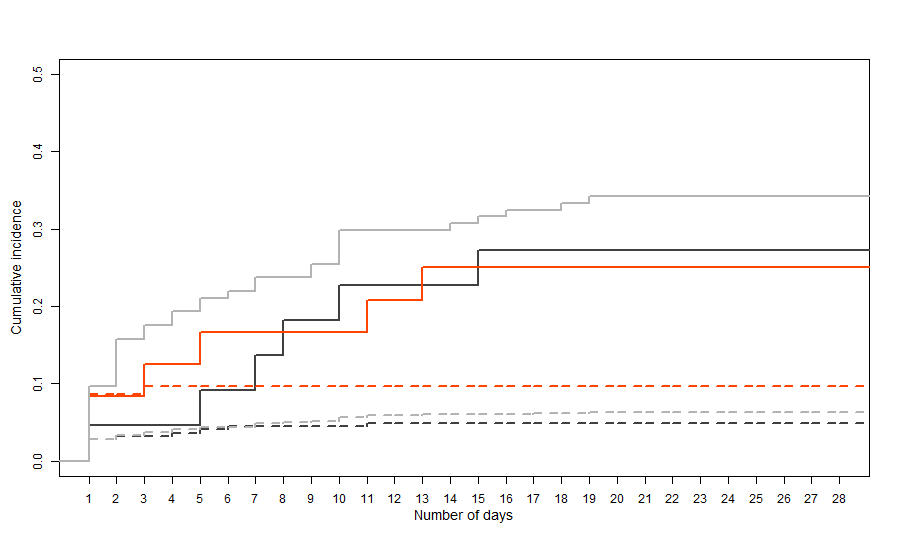

Supplement: Supplementary file 4 — Additional file 4: Fig. S2. a Time from ICU admission until at least 2 PECs during ICU stay (unweighted). b Time from ICU admission until death (unweighted). C Time from ICU admission until TLD during ICU stay (unweighted). TLD: treatment limitation decision, PEC: Perceptions of Excessive Care. [file 13613_2021_895_MOESM4_ESM.docx]
