# Supplementary material for: Outcomes of ICU patients with and without perceptions of excessive care: a comparison between cancer and non-cancer patients
Source: Ann Intensive Care. 2021 Jul 31;11:120. doi: 10.1186/s13613-021-00895-5 (PMC8325749; doi:10.1186/s13613-021-00895-5)
Supplement: Supplementary file 5 — Additional file 5: Table S3. Mortality and TLDs across subgroups (unweighted results) [file 13613_2021_895_MOESM5_ESM.docx]

Table S3 Mortality and TLDs across subgroups (unweighted results)

|  | **Uncontrolled cancer (n=117)** | **Controlled cancer**  **(n = 270)** | **Without cancer (n=1254)** | **p-value** |
| --- | --- | --- | --- | --- |
|  |  |  |  |  |
| **28 day mortality** |  |  |  |  |
| < 2 PECS | 23.7% | 19.8% | 15.4% | 0.03 |
| ≥ 2 PECs | 62.5% | 54.5% | 60.5% | 0.86 |
|  |  |  |  |  |
| **Treatment limitation decisions** |  |  |  |  |
| < 2 PECS | 9.7% | 4.8% | 6,2% | 0.27 |
| ≥ 2 PECs | 25% | 27,3% | 34,2% | 0.69 |
|  |  |  |  |  |
| **1 year mortality** |  |  |  |  |
| < 2 PECS | 46.2% | 38% | 23.9% | <0.001 |
| ≥ 2 PECs | 91.7% | 77.3% | 78.1% | 0.53 |
|  |  |  |  |  |
| **Combined endpoint** |  |  |  |  |
| < 2 PECS | 58.1% | 50.8% | 39.7% | 0.001 |
| ≥ 2 PECs | 95.8% | 81.8% | 86.8% | 0.49 |

Patients who were lost to follow up are not included in this table. *PEC*, perception of excessive care, *TLD*, treatment limitation decision.
